# Programmable Liposome Organization via DNA Origami Templates

**DOI:** 10.1021/jacs.5c05196

**Published:** 2025-07-02

**Authors:** Zhao Zhang, Zhaomeng Feng, Xiaowei Zhao, Zhiheng Yu, Edwin R. Chapman

**Affiliations:** † Howard Hughes Medical Institute, Department of Neuroscience, 5228 University of Wisconsin−Madison, Madison, Wisconsin 53705, United States; ‡ School of pharmacy, University of Wisconsin−Madison, Madison, Wisconsin 53705, United States; § Howard Hughes Medical Institute, 91336 CryoEM Shared Resource, Janelia Research Campus, 19700 Helix Drive, Ashburn, Virginia 20147, United States

## Abstract

Liposomes are essential vehicles for membrane protein reconstitution and drug delivery, making them vital tools in both in vivo and in vitro studies. However, the lack of robust techniques for the precise arrangement of these synthetic vesicles limits their potential applications. Here, we present a modular polymerization platform based on square DNA origami to template the formation and organization of liposomes. By programming the sequence, number, position, chirality, and flexibility of sticky ends on each square, we assemble uniformly sized liposomes into diverse two-dimensional (2D) arrays, as well as finite lattices and rings. Additionally, we demonstrate stepwise assembly and targeted disassembly, enabling dynamic structural control. These complex liposome architectures represent a significant advancement in the fields of biotechnology, nanotechnology, and bottom-up biology.

## Graphical abstract

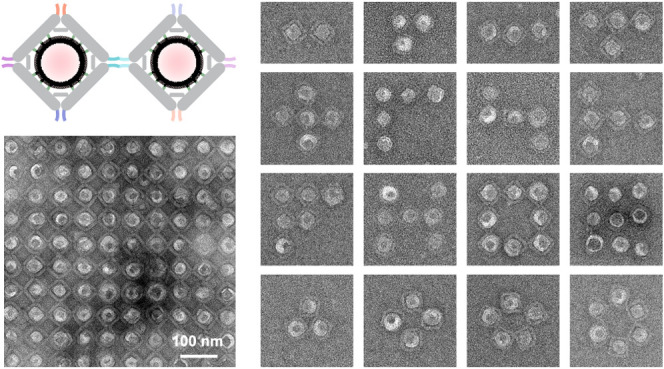

## Introduction

Spatiotemporally organized, membrane-bound compartments form the structural and functional foundation of eukaryotic cells. Mimicking and reconstructing these systems in vitro provide an invaluable platform to study lipid bilayers and membrane proteins in a controlled environment, free from the complexities of living cells. This approach is also an essential step toward a major scientific goal: building synthetic cells from the bottom up.^1,2^

Liposomes are artificial vesicles consisting of a lipid bilayer enclosing an aqueous “core”. They serve as powerful mimics of cellular organelles, and are widely used as model architectures for studying membrane biophysics and biochemistry, as well as for applications in drug delivery and chemical reaction compartmentalization.^3,4^ Advances in DNA nanotechnology have enabled precise control over the size, shape, and interparticle distance of small unilamellar vesicles (SUVs).^5,6^ However, higher-order liposome organization remains largely confined to one-dimensional (1D) strings or three-dimensional (3D) clusters.^7,8^ Given that the spatial positioning and arrangement of organelles are crucial for intracellular communication and transport, further progress in engineering complex liposome assemblies could significantly expand applications,^9^ such as facilitating cryogenic electron microscopy visualization,^10^ implementing proto-cellular communication,^11^ and generating protein arrays for biosensing.^12^

In this work, we develop a programmable polymerization system using square-shaped DNA origami to template the assembly and spatial arrangement of liposomes. We successfully design and create a variety of two-dimensional (2D) arrays, square lattices of up to nine units, and finite-size rings. Additionally, we demonstrate stepwise and reversible assembly, offering dynamic control over liposome organization. These membrane architectures, with high complexity and programmability, contribute a long-awaited asset to model membrane systems, bottom-up synthetic biology, and biotechnology.

## Results and Discussion

### DNA Origami Design

A suitable DNA origami structure^13^ for this study should fulfill two key functions: templating liposome formation and serving as a monomer to create larger assemblies. Although various DNA origami polymerization systems exist, none feature a central aperture large enough to accommodate vesicle formation.^14,15^ One exception is a modular nanocage capable of hosting a liposome; however, its polymerization is primarily restricted to a single direction due to its circular shape and the location of its interaction sites.^7^

Inspired by recent work by Li et al. on 2D assemblies using square DNA origami (SDO),^16,17^ we modified the design for interfacing with liposomes. Our system introduces several key changes: (1) employing a longer scaffold strand (8064-nt) to generate a square structure with an inner side length of ∼40 nm, matching the diameter of a synaptic vesicle; (2) rearranging staple strands to allocate inner handles for cholesterol functionalization; (3) breaking symmetry in select sticky ends (SEs) to enable chirality control during monomer binding. Two pairs of SEs with poly thymine (T) spacers extend from each vertex as needed (Figure 1c). Each vertex can be independently programmed with SEs of specific sequence, chirality, and flexibility, allowing for the creation of diverse polymerization products. Figure S1 provides the blueprint of the SDO we utilized.

**Figure 1:**
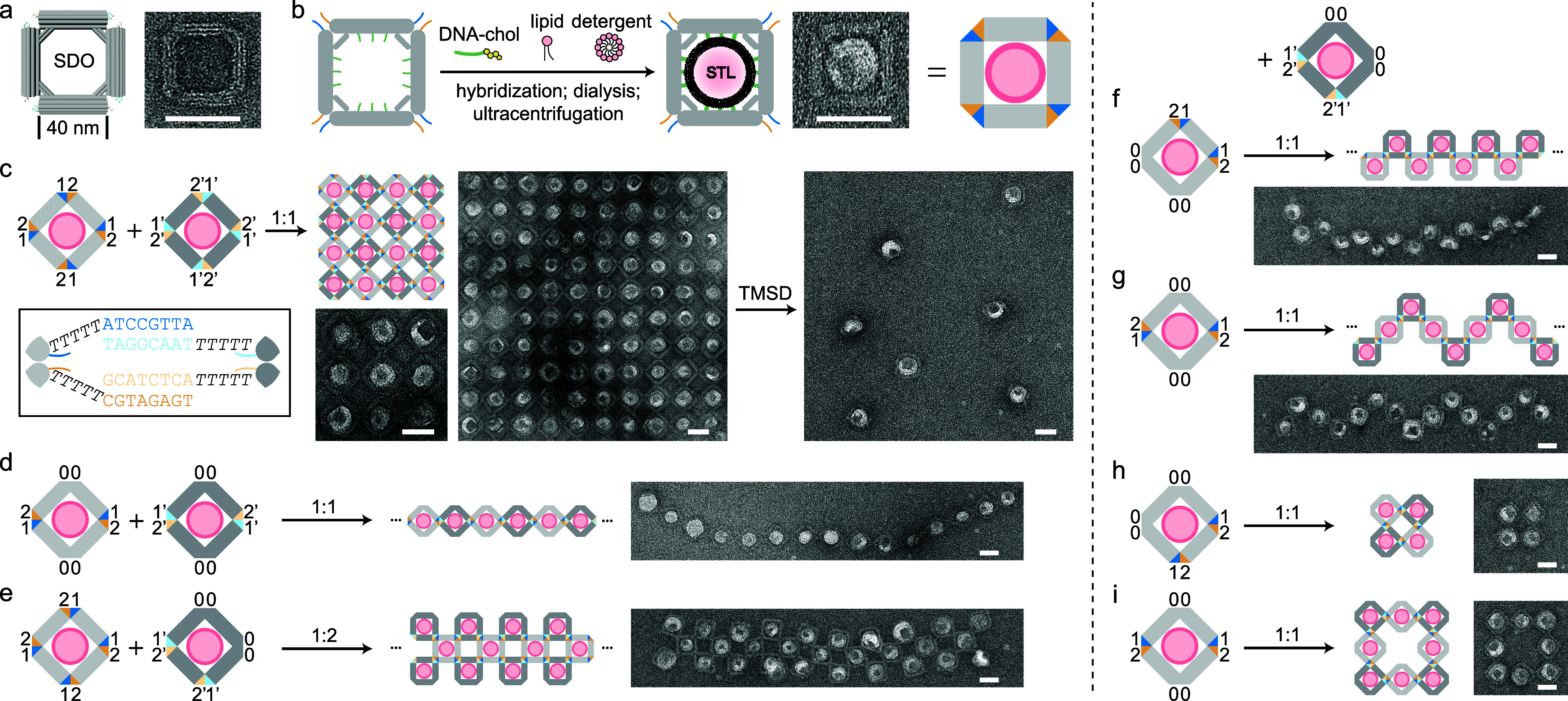
Liposome arrays templated by square DNA origami (SDO). (a) A 3D model and TEM image of SDO. (b) Schematics and a TEM image depicting the formation of an SDO-templated liposome (STL). (c) 2D liposome lattices formed by two STL variants. Sticky ends (SEs) are color-coded and numbered in the cartoon model. SE sequences and the T5 spacer are shown in the inset. A cropped TEM image presents a 10 × 10 liposome lattice, which can be disassembled via toehold-mediated strand displacement (TMSD). (d, e) Examples of diverse liposome arrays. (f–i) Four different STL units interacting with the same unit separately, generating distinct liposome patterns, including two additional arrays and two square-shaped oligomers. Scale bars: 50 nm.

Experimental procedures are detailed in the Methods. Negative-stain transmission electron microscopy (TEM) confirmed successful SDO formation (Figure 1a). Importantly, two SDO variants with complementary SEs at all four vertices assembled into large-scale 2D lattices within a 3 h annealing process (Figure S2), laying the groundwork for liposome organization.

### Liposome Arrays

SDO-templated liposomes (STLs) were prepared using an established protocol with minor modifications.^18^ Briefly, thirty-one cholesterol-modified single-stranded DNA (ssDNA) were hybridized to complementary handles on the SDO in the presence of lipids and detergent, followed by dialysis to remove the detergent (Figure 1b). During this process, liposomes assembled on the inner face of the SDO through cholesterol incorporation,^19^ with their size confined by the origami frame. Dialyzed samples were purified via isopycnic centrifugation, and a total of 12 fractions were collected. Fractions 3 to 6, counting from top to bottom, contained monodispersed STLs with decreasing liposome size (Figure S3). Unless specified otherwise, fraction 5 was used for all subsequent experiments. Both SDO and STL polymers were characterized by TEM.

While DNA nanostructures have been used to organize liposomes into 1D chains and 3D clusters, the self-assembly of 2D liposome lattices has remained elusive. Our STL system addresses this gap by enabling polymerization in both x- and *y*-directions. TEM images confirmed that individual STL units assembled into micrometer-scale square lattices, marking the successful generation of a regular 2D SUV array (Figures 1c and S4). Liposome occupancy rate exceeded 98%, and the integrity of the liposomal membranes was validated by cryo-electron microscopy (Figure S5). Furthermore, by introducing displacing strands, the lattice could be disassembled into STL monomers via toehold-mediated strand displacement (TMSD),^20^ mimicking the clustering and release of synaptic vesicles at the synapse.^21^

The strong binding efficiency between STL monomers prompted us to explore additional strategies for array formation. For example, two STL units with complementary SEs on a pair of opposite vertices were polymerized in a head-to-tail manner, forming 1D strings of up to 20 units, with a median degree of polymerization (DP) of 7 (Figures 1d and S6, see Table S1 for DP quantification by TEM). In another reaction, a three-layer ribbon was assembled, where one central layer of STLs with SEs on all four vertices was sandwiched between two layers of STLs with SEs on only two neighboring vertices (Figures 1e and S7). In this configuration, the precise chirality of SEs ensures correct binding orientation.

To further demonstrate the versatility of SE programming, we designed a series of copolymers that share a common monomer with SEs on two neighboring vertices. Variations in the chirality and position of the SEs on the second monomer led to distinct assembly products, including two additional types of liposome arrays and two square-shaped liposome oligomers (Figures 1f–i and S8–11). The yields of correctly assembled tetramer and octamer, as quantified by TEM, are 38 and 23%, respectively (Table S2), motivating further exploration of finite liposome lattices. Notably, STL oligomerization proceeded efficiently even when incubated at room temperature or 4 °C (Figure S12), conditions more compatible with potential applications involving proteins.

### Finite Lattices of Liposomes

The goal of bottom-up biology is constructing synthetic systems from molecular building blocks that can replicate or even surpass cellular functions.^22^ While the open-ended 1D and 2D liposome lattices described above resemble cellular structures such as synaptic vesicle chains^23^ and secretory vesicle clusters,^24^ it is crucial to achieve precise control over the finite assembly of liposome oligomers.^25^ Unlike previous studies limited to liposome dimer formation,^8,26^ STL enables the assembly of complex patterns far beyond dimers (Figure S13), by independently programming the sequence, position, and chirality of SEs on three STL units.

We first used a series of STL monomer (LM1) with chiral SEs (se1/2) positioned on varying numbers (N) of vertices, reacting them with a common STL monomer (LM2), which featured complementary SEs (se2′/1′) on one vertex and an additional set of SEs (se3′) on the opposite vertex. When combined in a 1-to-*N* ratio, LM1 and LM2 assembled into distinct liposome structures, including a cross-shaped 5-mer, a T-shaped tetramer, and two types of trimers (Figures 2a–d and S14–17; see Table S2 for a summary of assembly yields quantified by gel and TEM). To further extend these assemblies, we introduced a third STL monomer (LM3) carrying complementary SEs (se3) on one vertex. LM3 specifically bound to the se3′ site on LM2, appending additional layers to the liposomal structures. TEM images confirmed the formation of cross-shaped 9-mers, T-shaped 7-mers, as well as L-shaped and linear 5-mers (Figures 2a-d and S18–21).

**Figure 2:**
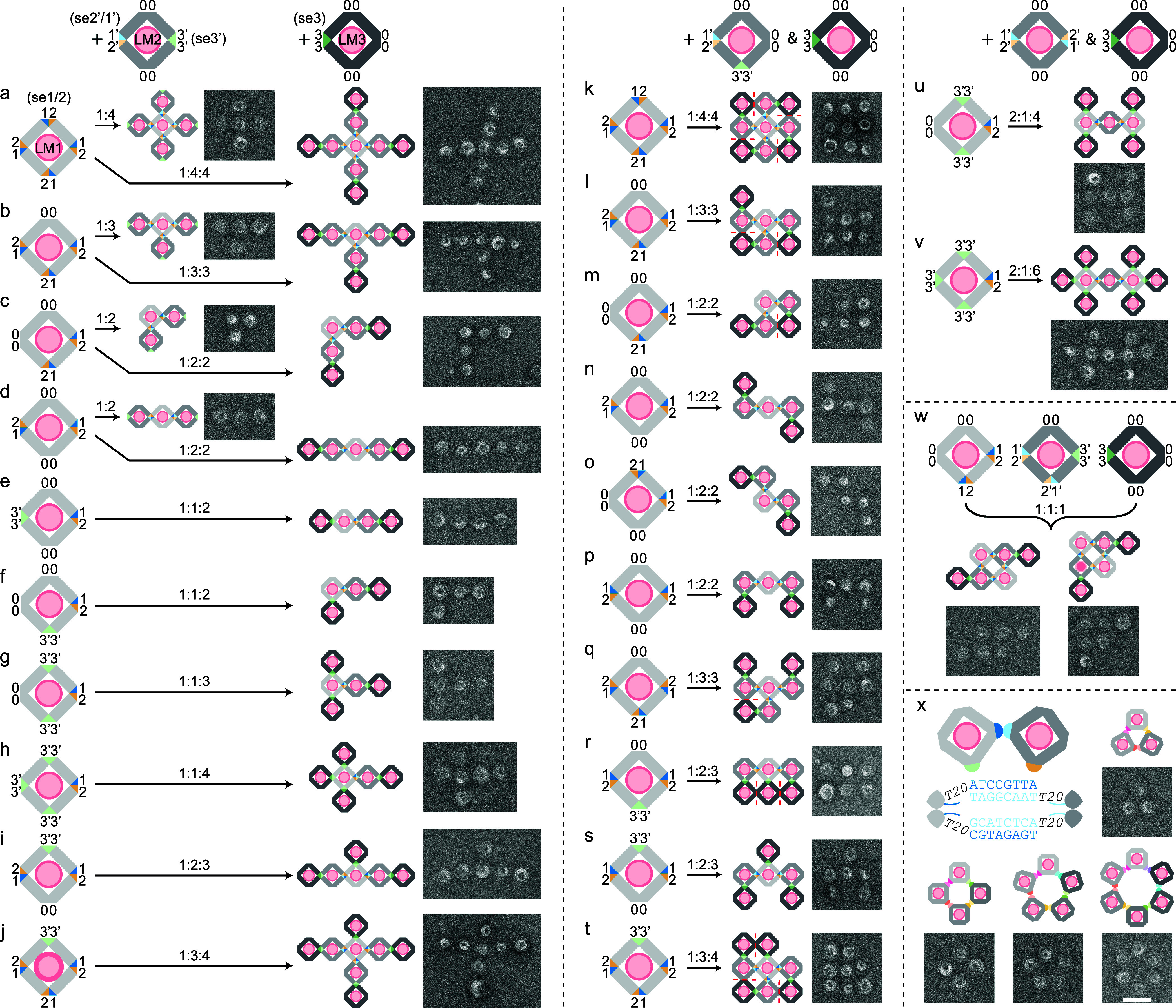
Finite liposome lattices and rings. The sequence (se1/2 or se3), number (one to four), position, and chirality (se1/2 or se2/1) of sticky ends on each STL are programmed independently. (a–v) Assembly of three units (LM1-LM3): LM1 with varying numbers of se1/2 and se3′; LM3 with one se3; LM2 contains one se2′/1′ and one opposite se3′ (a–j), or one se2′/1′ and one neighboring se3′ (k–t), or two se2′/1′ (u–v). (w) Three units forming two distinct 6-mer products in the same reaction. (x) Extending the spacers to T20 and encoding STL units with up to 6 SE sequences generates trimer, tetramer, 5-mer, and 6-mer heterorings. A cartoon model and a cropped TEM image are shown for each construct. Scale bar: 100 nm.

Next, we incorporated se3′ into LM1, enabling it to also bind to LM3 through its se3 site. By adjusting the position and number of se3′ sites, we obtained a range of new configurations. For instance, modifying LM1 to contain one set of se1/2 and one, two, or three sets of se3′ and mixing it with LM2 and LM3 resulted in structures such as a linear or L-shaped tetramer, a T-shaped 5-mer, or a cross-shaped 6-mer (Figures 2e–h and S22–25). Additionally, introducing a single se3′ site to LM1 containing two or three sets of se1/2 led to the formation of a T-shaped 6-mer and a cross-shaped 8-mer through interactions with LM2 and LM3 (Figures 2i,j and S26–27).

Following this design principle, we repositioned the se3′ site on LM2 from the opposite vertex to the neighboring vertex of se2′/1′, allowing it to interact with LM1 and LM3 in a new manner. This simple adjustment yielded a distinct set of oligomers, including a square-shaped 9-mer, a boot-shaped 7-mer, and two types of 5-mers (Figures 2k–n and S28–31). Moreover, since the modified LM2 featured an asymmetric SE pattern, simply reversing the chirality of one of LM1’s SEs (e.g., from se1/2 to se2/1) resulted in two additional 5-mers and one 7-mer configurations (Figures 2o–q and S32–34). Further structural diversity was achieved by introducing se3′ to LM1, producing new 6-mers and 8-mers with LM2 containing one se2′/1′ site (Figures 2r–t and S34–36) or 7-mers and 9-mers with LM2 containing two opposite se2′/1′ sites (Figures 2u–v and S37–38).

Finally, we introduced LM3, along with extra binding sites on LM2, into a reaction in which a square-shaped tetramer had formed (Figure 1h). Interestingly, TEM images revealed the coexistence of two distinct 6-mer products (Figures 2w and S39). This variation arose because the interactions between LM1 and LM2 remained valid even when one monomer was flipped—a phenomenon not observable in the symmetric tetramer core but clearly evident when a third monomer was attached. Unlike previous systems with well-defined assemblies, this ability to generate multiple products introduces potential for multifunctionality and regulatory control, akin to their cellular counterparts.

### Liposome Rings with Flexible Spacers

All the designs discussed above incorporated a T5 spacer between the origami body and the SE region, providing rigidity that maintained a 180-degree angle for monomer interaction. To increase assembly flexibility, we extended the spacers from T5 to T20,^17^ allowing the SEs to move more freely. By integrating three sets of chiral T20 SEs into two neighboring vertices of three monomers, we successfully constructed a heterotrimer ring with a 120/60-degree connection angle (Figures 2x and S40). Further expansion of this approach enabled the formation of tetramer, 5-mer, and 6-mer rings by using four, five, or six sets of T20 SEs, respectively (Figures 2x and S41–43). This ability to fine-tune polymer flexibility opens the door to more diverse and complex assembly products, such as 2D tessellations^27^ and even 3D architectures.^28^

### Dynamic Oligomerization of Liposomes

Reconstructing and spatially organizing membranous compartments is a fundamental step in bottom-up biology. However, to truly replicate cellular functions such as vesicle trafficking and recycling, liposome ensembles must be capable of assembling and disassembling in response to specific stimuli.^29^ We have already demonstrated that the 2D liposome lattice can break down into individual vesicles (Figure 1c). Here, we present two additional examples of controlled liposome oligomerization.

In the first example, a T-shaped liposome heterotetramer was assembled stepwise. The central monomer contained three distinct SEs on separate vertices. By sequentially adding three STLs with complementary SEs on a single vertex, a dimer, trimer, and ultimately a tetramer was formed (Figures 3a and S44). The second example utilized TMSD to achieve targeted disassembly of a heterotetramer. Since the central monomer’s three SEs had distinct sequences, introducing specific complementary displacing strands selectively disrupted the interactions, leading to unique disassembly products (Figures 3b and S45). These results demonstrate that STL oligomerization can be precisely controlled and reversed, offering a significant step toward mimicking cellular processes like synaptic vesicle pool maintenance^30^ and the Golgi apparatus’s role as a vesicle hub.^31^

**Figure 3:**
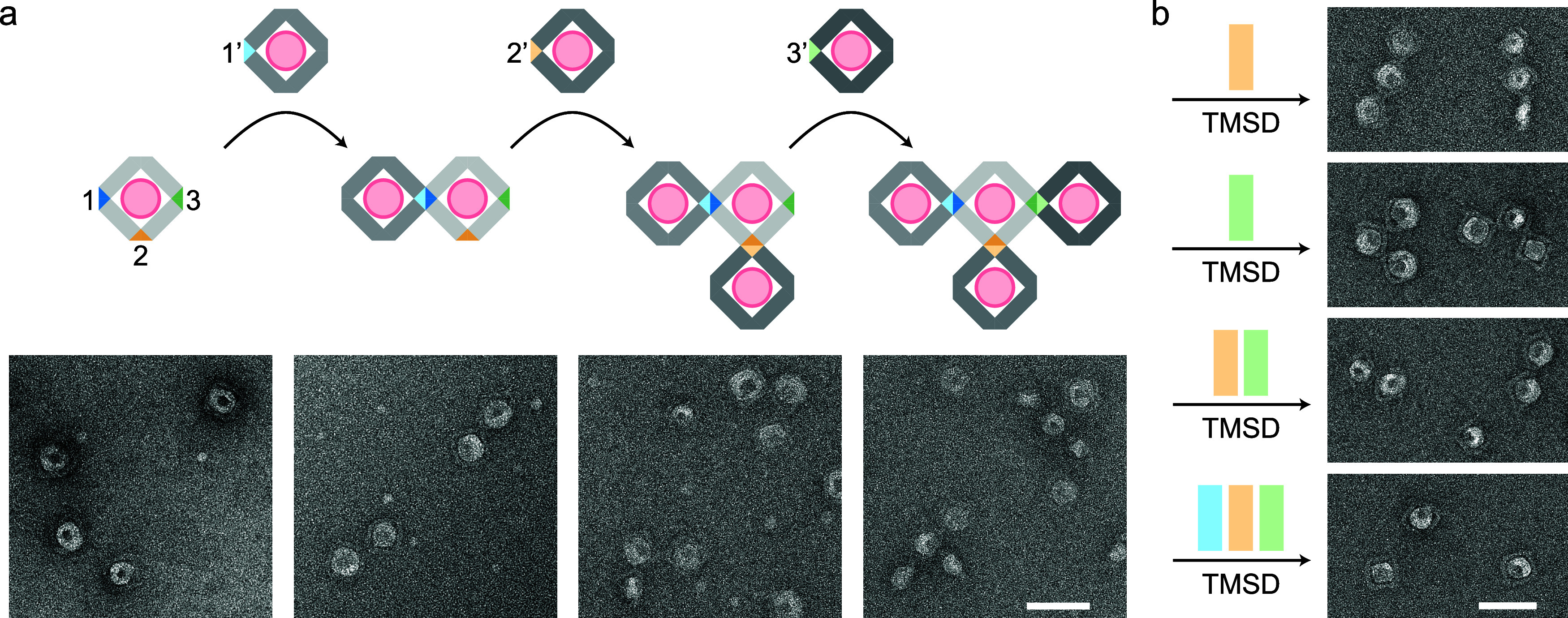
Dynamic control of liposome oligomerization. (a) Stepwise assembly of a heterotetramer with three different sticky ends (1, 2, or 3). (b) Targeted disassembly of a preformed heterotetramer using TMSD. A specific set of displacing strands disrupts selective interactions, leading to unique products. A cropped TEM image is shown for each product. Scale bars: 100 nm.

## Conclusions

Compartmentalization is one of the most defining characteristics of cells. As a result, reconstructing membrane-bounded organelles in vitro is essential for studying cellular behaviors in controllable environments, understanding the origin of life, and designing vehicles for cell interaction.^32,33^ While artificial vesicles, known as liposomes, hold great promise for applications such as protein reconstitution and drug delivery,^34^ the lack of spatial and dynamic control over their higher-order assembly limits their functionality compared to natural cellular structures.

In this work, we established a liposome patterning platform based on DNA-templated liposome formation and square-shaped DNA origami polymerization (see Figure S46 for gel analysis of origami polymers). By fine-tuning the SE properties on each vertex of the square, including sequence, position, chirality, and flexibility, we arranged liposomes into micrometer-scale arrays, as well as a variety of finite lattices and rings ranging from dimers to 9-mers. Furthermore, we demonstrated stepwise assembly and targeted disassembly, providing dynamic control over liposome oligomerization.

The complexity and programmability of liposome organization achieved here (Figure S47) represent a major step forward in advancing model membranes, organelle mimics, and nanoreactor networks, broadening the scope of synthetic biology and nanotechnology. Prospective applications of the liposome array system include addressable (proteo)­liposome arrays for electron microscopy visualization of protein–protein or protein–membrane interactions, nanoreactor networks for chemical and biochemical synthesis, in vitro assays to study SNARE-mediated membrane fusion with defined stoichiometry, bottom-up construction of synapse-like connections and organelle contacts, among others (see the sections titled “Potential Applications of STL Arrays” and “Limitations and Future Improvements of STL Arrays” in the Supporting Information for more details).

## Experimental Methods

### DNA Origami Design and Preparation

The SDO structure was revised from a previous design^16^ using cadnano (cadnano.org). Origami scaffold strands (8,064 nt) were produced using and M13-derived bacteriophages.^35^ DNA origami was assembled by mixing 8064-nt scaffold strands (50 nM) with a selected pool of staple strands (300 nM each, IDT) in a *folding buffer* (5 mM Tris-HCl, 12.5 mM MgCl_2_, 1 mM EDTA, pH 8.0). The mixture was annealed in a PCR thermocycler (Bio-Rad) through a gradual cooling process from 80 to 24 °C over 36 h. The assembled products were purified by rate-zonal centrifugation in glycerol gradients, as previously described.^36^ Origami concentration was determined by Nanodrop (Thermo Fisher Scientific).

### Origami-Templated Liposome Preparation

STL preparation was adapted from a previously established protocol.^18^ In brief, SDO with thirty-one ssDNA handles on the inner face of its sides was functionalized with cholesterol-modified oligonucleotides (antihandle) via DNA hybridization. Additionally, four Cy5-modified antihandles were hybridized onto a distinct set of handles on each origami. The antihandle to handle ratio was maintained at 2:1. The mixture was incubated at 37 °C for 1 h in the presence of 1% octyl β-d-glucopyranoside (OG).

A 1 μmol lipid mixture was prepared in chloroform with the following composition: 74.8% 1,2-dioleoyl-*sn*-glycero-3-phosphocholine (DOPC), 20% 1,2-dioleoyl-*sn*-glycero-3-phospho-l-serine (DOPS), 5% 1,2-dioleoyl-*sn*-glycero-3-phosphoethanolamine-*N*-[methoxy­(polyethylene glycol)-2000] (PEG2k-PE), and 0.2% 1,2-dioleoyl-*sn*-glycero-3-phosphoethanolamine-*N*-(lissamine rhodamine B sulfonyl) (Rhod-PE). The lipid solution was dried under nitrogen flow for 10 min, followed by vacuum drying for 1 h. Dried lipids were resuspended in 100 μL *hydration buffer* (25 mM HEPES, 100 mM KCl, pH 7.4) and vortexed for 10 min.

60 nmol rehydrated lipids were combined with 1.2 pmol cholesterol-labeled origami, and the solution was diluted to 120 μL with *hydration buffer* containing 1% OG. The mixture was gently shaken for 30 min at room temperature before being transferred into a dialysis cassette (7 kDa molecular weight cutoff). After dialysis overnight against 2 L of *hydration buffer*, the samples were purified using isopycnic centrifugation in an iodixanol gradient (6%–30%). A total of 12 50 μL fractions were collected, and the fifth fraction from the top was selected for polymerization.

### Polymerization of SDO and STL

For each reaction, the corresponding SDO or STL variants were mixed and annealed by gradually cooling from 40 to 22 °C over 3 h (−0.1 °C per minute). The variant ratio was determined by their copy number in the intended products. If present in equal amounts, equimolar stoichiometry was used; otherwise, peripheral monomers were doubled to fully saturate the binding sites on core monomers. Polymer products were characterized by TEM without further purification.

### Dynamic Control of SDO or STL Polymers

The stepwise assembly began with an STL monomer. A new STL variant (50% excess) was added to the previous sample and annealed from 40 to 22 °C over 3 h. A 5 μL aliquot was collected for TEM characterization. This cycle was repeated three times.

For TMSD, the corresponding displacing strands were added to the preformed polymer to achieve a final concentration of 10 μM. The mixture was then incubated at 37 °C for 2 h before TEM characterization.

### Negative-Stain TEM

A 5 μL sample was pipetted onto a glow-discharged Formvar/carbon-coated copper grid (Ted Pella) and stained with 2% uranyl acetate (Electron Microscopy Sciences) for 1 min. Imaging was performed using a Talos F200C scanning transmission electron microscope (Thermo Fisher Scientific).

### Cyro-EM

EM grids (Lacey carbon with an ultrathin carbon layer (2–3 nm), 300 mesh, Au, Ted Pella Inc.) were glow discharged for 15 s with a current of 15 mA and a controlled air pressure of 0.39 mbar, using a Pelco EasiGlow glow discharger (Ted Pella Inc.). The grids were then loaded into a Vitrobot Mark IV plunge freezer (Thermo Fisher Scientific), with the sample chamber set to 4 °C and 100% relative humidity. A 3 μL aliquot of the liposome/DNA sample was applied to the glow-discharged front surface of the grid and then automatically blotted from both sides with filter paper. Blotting times ranged between 5 to 7 s, after which the grid was plunged into liquid ethane for vitrification.

Vitrified liposome/DNA samples were imaged using a 300 kV Titan Krios (Thermo Fisher Scientific), equipped with a high-brightness Field Emission Gun (x-FEG), a spherical aberration corrector, and a GIF BioContinuum HD energy filter (Gatan, Inc.). Cryo-EM images were acquired on a K3 direct electron detector (Gatan, Inc., CA) at a nominal magnification of 42,000×, corresponding to a pixel size of 1.698 Å. To reduce beam-induced motion, a movie was recorded for each data point. Motion correction was applied to each movie, resulting in a corrected and averaged cryo-EM image for each data point.

### Gel Electrophoresis

Agarose gels (0.8% w/v) were prepared in 1× TAE buffer supplemented with 10 mM MgCl_2_ and 0.5 μg/mL ethidium bromide. A 5 μL sample was loaded into individual wells. Electrophoresis was carried out in 1× TAE buffer at 40 V for approximately 5 h. DNA bands were visualized under UV illumination using a ChemiDoc MP system (Bio-Rad) and subsequently analyzed using ImageJ software.

## Supplementary Material



## Notes

DNA sequences of sticky ends, a list of reagents, cadnano design of SDO, additional TEM images for each construct, agarose gel electrophoresis images, summary tables of assembly yields, a discussion of potential applications and limitations (PDF)
