# Supplementary material for: Programmable Liposome Organization via DNA Origami Templates
Source: J Am Chem Soc. 2025 Jul 2;147(28):24548–54. doi: 10.1021/jacs.5c05196 (PMC12272708; doi:10.1021/jacs.5c05196)
Supplement: Supplementary file 1 [file ja5c05196_si_001.pdf]

Programmable Liposome Organization via DNA Origami Templates

Zhao Zhang, Zhaomeng Feng, Xiaowei Zhao, Zhiheng Yu and Edwin R. Chapman

Supplementary Information

TABLE OF CONTENT

Abbreviations 1

Materials 2

Supplementary Figures 4

Figure S1 4

Figure S2 5

Figure S3 6

Figure S4 7

Figure S5 8

Figure S6 9

Figure S7 10

Figure S8 11

Figure S9 12

Figure S10 13

Figure S11 14

Figure S12 15

Figure S13 16

Figure S14 17

Figure S15 18

Figure S16 19

Figure S17 20

Figure S18 21

Figure S19 22

Figure S20 23

Figure S21 24

Figure S22 25

Figure S23 26

Figure S24 27

Figure S25 ... 28
Figure S26 ... 29
Figure S27 ... 30
Figure S28 ... 31
Figure S29 ... 32
Figure S30 ... 33
Figure S31 ... 34
Figure S32 ... 35
Figure S33 ... 36
Figure S34 ... 37
Figure S35 ... 38
Figure S36 ... 39
Figure S37 ... 40
Figure S38 ... 41
Figure S39 ... 42
Figure S40 ... 44
Figure S41 ... 45
Figure S42 ... 46
Figure S43 ... 47
Figure S44 ... 48
Figure S45 ... 50
Figure S46 ... 51
Figure S47 ... 52
Table S1 ... 53
Table S2 ... 54
Potential Applications of STL Arrays ... 59
Limitations and Future Improvements of STL arrays ... 64
References ... 66

Abbreviations

Abbreviation	Full name
1D	one-dimensional
2D	two-dimensional
3D	three-dimensional
18HB	eighteen helix bundle
chol	cholesterol
cryo-EM	cryogenic electron microscopy
DNA	deoxyribonucleic acid
DOPC	1,2-dioleoyl-sn-glycero-3-phosphocholine
DOPS	1,2-dioleoyl-sn-glycero-3-phospho-L-serine
PEG2k-PE	1,2-dioleoyl-sn-glycero-3-phosphoethanolamine-N-[methoxy(polyethylene glycol)-2000]
Rhod-PE	1,2-dioleoyl-sn-glycero-3-phosphoethanolamine-N-(lissamine rhodamine B sulfonyl)
EDTA	ethylenediaminetetraacetic acid
HEPES	4-(2-hydroxyethyl)-1-piperazineethanesulfonic acid
LM1/2/3	STL monomer 1/2/3
OG	n-octyl-β-D-glucopyranoside
Oligo	oligonucleotide
nt	nucleotide
ssDNA	single-stranded DNA
SE	sticky end
SDO	square DNA origami
STL	SDO-templated liposome
SNARE	soluble NSF attachment protein receptor
GUV	giant unilamellar vesicle
SUV	small unilamellar vesicle
T	thymine
TEM	transmission electron microscopy
TMSD	toehold-mediated strand displacement

Materials

All oligonucleotides were purchased from Integrated DNA Technologies (IDT) and used without further purification. Cholesterol-modified oligos (/5Chol-TEG/GTGAGTTGTGGTAGATAATTT) and Cy5-modified oligos (/5Cy5/GAATCGGTCACAGTACAACCG) were HPLC-purified by IDT. All sticky ends (SEs) are extended from the 3' of the corresponding staple strand. See Supplementary Fig. 1 for the origami design. SE sequences used in this study are shown below:

Name	Sequence (5'->3')	Usage in figures
se1	*TTTTT*ATCCGTTA	together forming se1/2 or se2/1 in Fig. 1 & Fig. 2a-2w.
se2	*TTTTT*CGTAGAGT	
se1'	*TTTTT*TAACGGAT	together forming se1'/2' or se2'/1' in Fig. 1 & Fig. 2a-2w.
se2'	*TTTTT*ACTCTACG	
se3	*TTTTT*GCCGCAGGATAC	Fig. 2a-2w.
se3'	*TTTTT*GTATCCTGCGGC	Fig. 2a-2w.
se11	*TTTTTTTTTTTTTTTTTTTT*ATCCGTTA	SE pair 1 in Fig. 2x.
se12	*TTTTTTTTTTTTTTTTTTTT*CGTAGAGT	
se11'	*TTTTTTTTTTTTTTTTTTTT*TAACGGAT	
se12'	*TTTTTTTTTTTTTTTTTTTT*ACTCTACG	
se21	*TTTTTTTTTTTTTTTTTTTT*GCAGGATA	SE pair 2 in Fig. 2x.
se22	*TTTTTTTTTTTTTTTTTTTT*ACGCATAA	
se21'	*TTTTTTTTTTTTTTTTTTTT*TATCCTGC	
se22'	*TTTTTTTTTTTTTTTTTTTT*TTATGCGT	
se31	*TTTTTTTTTTTTTTTTTTTT*CTGGCTTA	SE pair 3 in Fig. 2x.
se32	*TTTTTTTTTTTTTTTTTTTT*GAGAAGTC	
se31'	*TTTTTTTTTTTTTTTTTTTT*TAAGCCAG	
se32'	*TTTTTTTTTTTTTTTTTTTT*GACTTCTC	
se41	*TTTTTTTTTTTTTTTTTTTT*GCAAGAGT	SE pair 4 in Fig. 2x.
se42	*TTTTTTTTTTTTTTTTTTTT*ATCTCACC	
se41'	*TTTTTTTTTTTTTTTTTTTT*ACTCTTGC	
se42'	*TTTTTTTTTTTTTTTTTTTT*GGTGAGAT	
se51	*TTTTTTTTTTTTTTTTTTTT*CTCACGAT	SE pair 5 in Fig. 2x.
se52	*TTTTTTTTTTTTTTTTTTTT*GTAGTCCA	
se51'	*TTTTTTTTTTTTTTTTTTTT*ATCGTGAG	
se52'	*TTTTTTTTTTTTTTTTTTTT*TGGACTAC	
se61	*TTTTTTTTTTTTTTTTTTTT*GGGTGTAT	SE pair 6 in Fig. 2x.
se62	*TTTTTTTTTTTTTTTTTTTT*TAGCTGTC	
se61'	*TTTTTTTTTTTTTTTTTTTT*ATACACCC	
se62'	*TTTTTTTTTTTTTTTTTTTT*GACAGCTA	
se71t	*TTTTT*CGTAGAGTACGCATAAATAT	SE pair 1 in Fig. 3. Toehold region is underlined.
se71'	*TTTTT*GCGTACTCTACG	
se72t	*TTTTT*GCTGGCTTAGAGAAGTCCCG	SE pair 2 in Fig. 3. Toehold region is underlined.
se72'	*TTTTT*CTCTAAGCCAGC	
se73t	*TTTTT*GCCGCAGGATACAGAATACG	SE pair 3 in Fig. 3. Toehold region is underlined.
se73'	*TTTTT*GTATCCTGCGGC	
se71t'	CGGGACTTCTCTAAGCCAGC	displacing strand for SE pair 1 in Fig. 3b.
se72t'	ATATTTATGCGTACTCTACG	displacing strand for SE pair 2 in Fig. 3b.
se73t'	CGTATTCTGTATCCTGCGGC	displacing strand for SE pair 3 in Fig. 3b.

Notable chemicals used in this study:

Chemical	Company	Catalog #
DOPC	Avanti Polar Lipids	850375C
DOPS	Avanti Polar Lipids	840035C
PEG2k-PE	Avanti Polar Lipids	880130C
Rhod-PE	Avanti Polar Lipids	810150C
Chloroform	Sigma-Aldrich	CX1058
OG	Goldbio	O-110
0.5 M EDTA solution	Thermo Fisher Scientific	J15694.AE
Trizma® base	Sigma-Aldrich	T4661
HEPES	Sigma-Aldrich	H4034
Magnesium chloride hexahydrate	Sigma-Aldrich	M9272
Potassium chloride	Sigma-Aldrich	P4504
Glycerol	Thermo Fisher Scientific	BP229-4
Iodixanol (OptiPrep™)	Cosmo Bio USA	AXS-1114542
UltraPure™ Agarose	Thermo Fisher Scientific	16500500
Ethidium bromide solution	Bio-Rad	1610433
2% Uranyl Acetate Solution	Electron Microscopy Sciences	22400-2

Notable labware used in this study:

Labware	Company	Catalog #
SW50.1 - Box Of 50 1/2" X 2" Polyclear™ Open Top Ultracentrifuge Tubes	BioComp	151-513
0.8 mL, Open-Top Thinwall Ultra-Clear Tube, 5 x 41mm	Beckman Coulter	344090
Amicon Ultra-0.5 Centrifugal Filter Unit 30kDa	Millipore	UFC503096
Slide-A-Lyzer™ 7K MWCO Dialysis Cassettes (0.5mL)	Thermo Fisher Scientific	66373
Formvar/Carbon 400 mesh, Copper approx. grid hole size: 42µm	Ted Pella	01754-F

Lipid compositions used in this study:

Lipids	Percentage
DOPC	74.8%
DOPS	20%
PEG2k-PE	5%
Rhod-PE	0.2%

Supplementary Figures

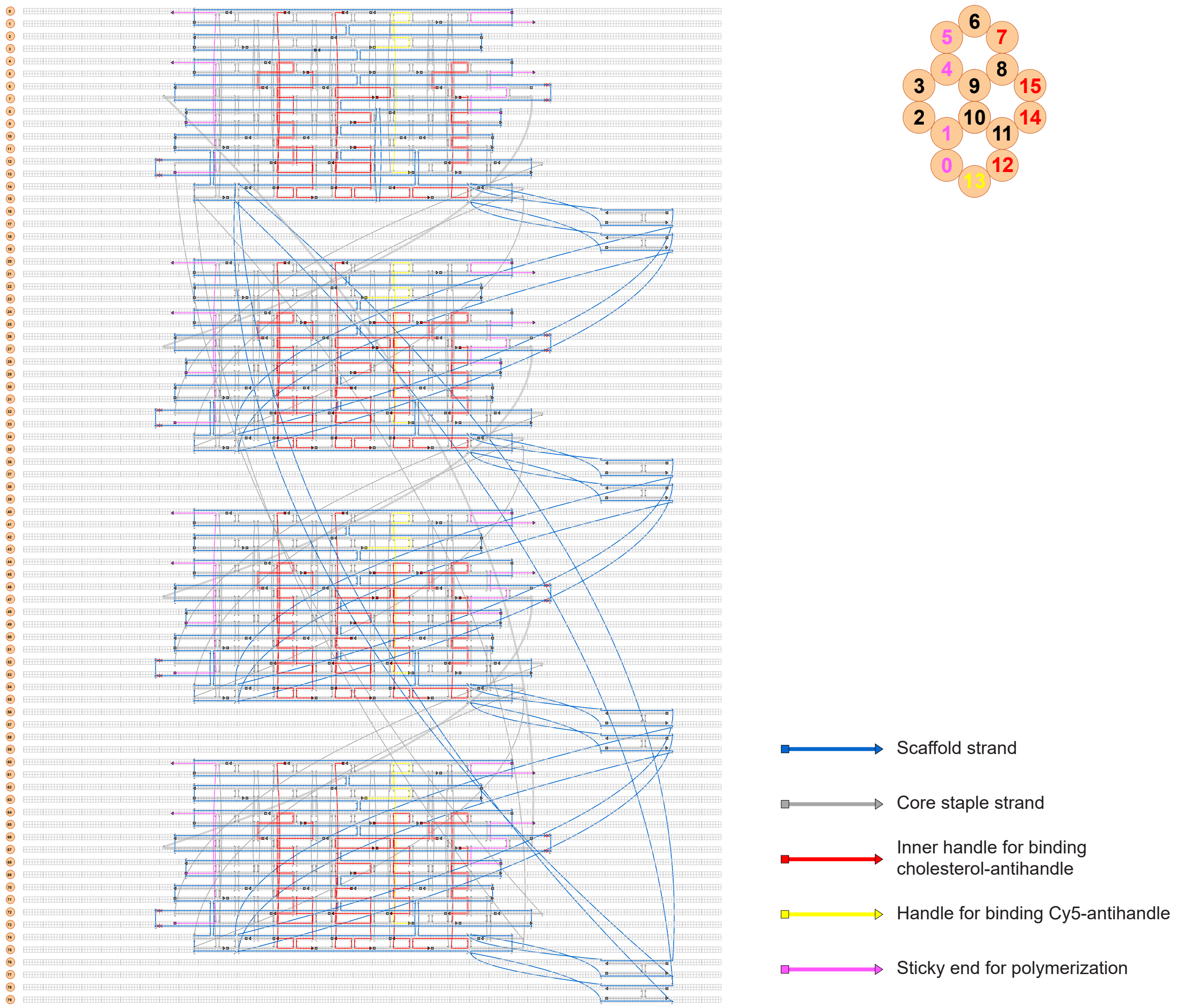

Figure S1. Cadnano design of the square DNA origami (SDO) used in this study.

This design is a revised version of a previously reported structure. DNA helices are arranged in a close-packed hexagonal lattice. Each side of the square consists of a 16-helix bundle (16HB) rod with an average length of 120 base pairs (bp). To maintain 90-degree interior angles, two 2HB supporting structures are positioned on the inner side of each vertex.

Each end of the 16HB sides extends a pair of single-stranded DNA (ssDNA) known as sticky ends, resulting in two pairs of sticky ends (magenta) at each vertex for monomer association. A total of 31 staples with their 3' ends pointing inward (red) have ssDNA extensions (handles), which can hybridize with cholesterol-modified oligonucleotides (anti-handles). Additionally, a set of 4 handles (yellow) is designed for hybridization with Cy5-labeled anti-handles. All other staples are shown in grey, while the scaffold strand (p8064) is depicted in blue.

The original cadnano file and DNA sequences are provided upon request.

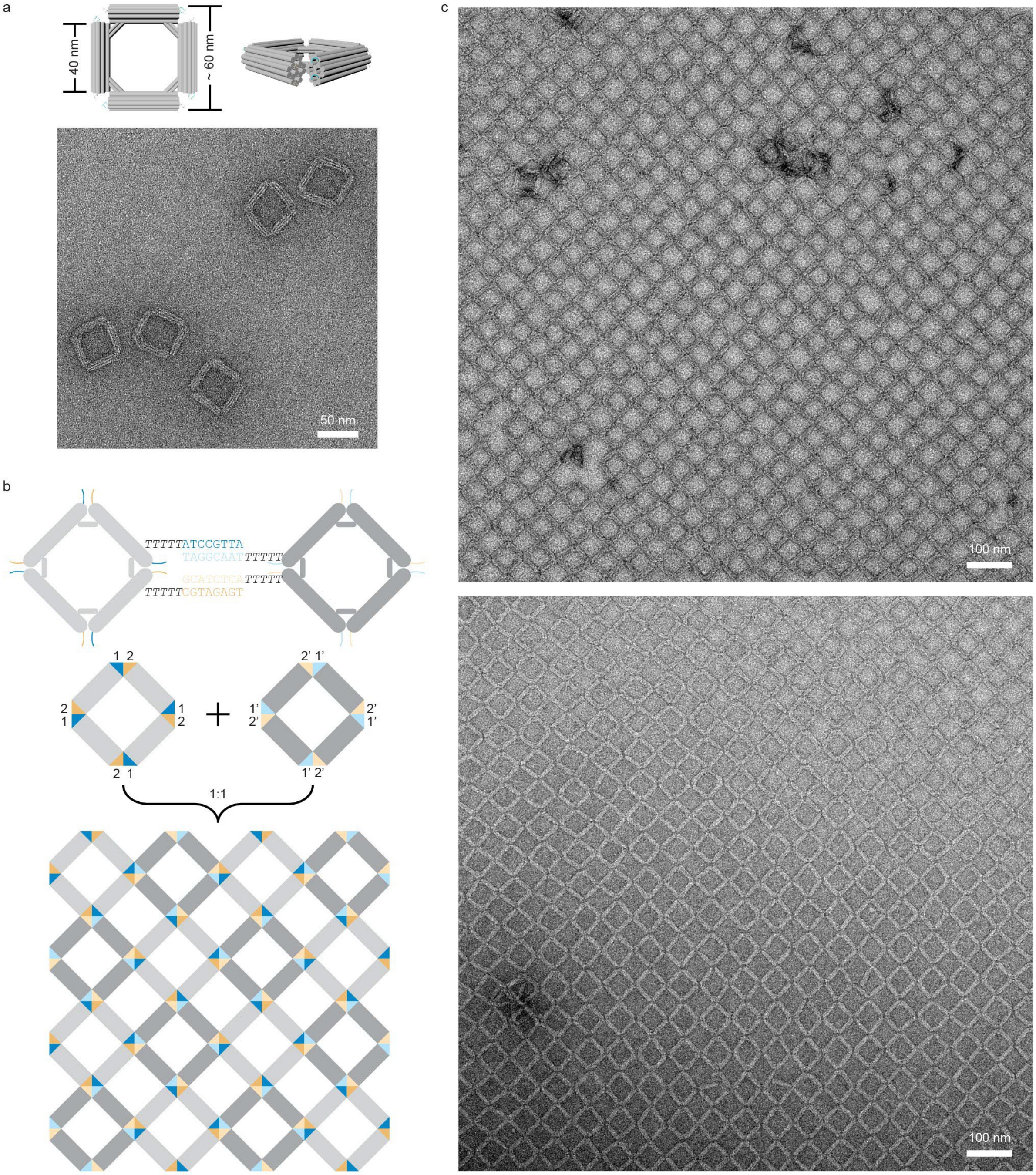

Figure S2. SDO monomers and lattices. (a) Top: a 3D model generated by Maya (Autodesk) depicting the top and side views of an SDO. Bottom: a negative-stain transmission electron microscopy (TEM) image confirming the correct assembly of the SDO. (b-c) Schematics and TEM images illustrating the formation of square lattices by two SDO variants in a 1:1 ratio. Both variants feature chiral SEs on all four vertices, with complementary SE sequences, as shown in the top panel of (b).

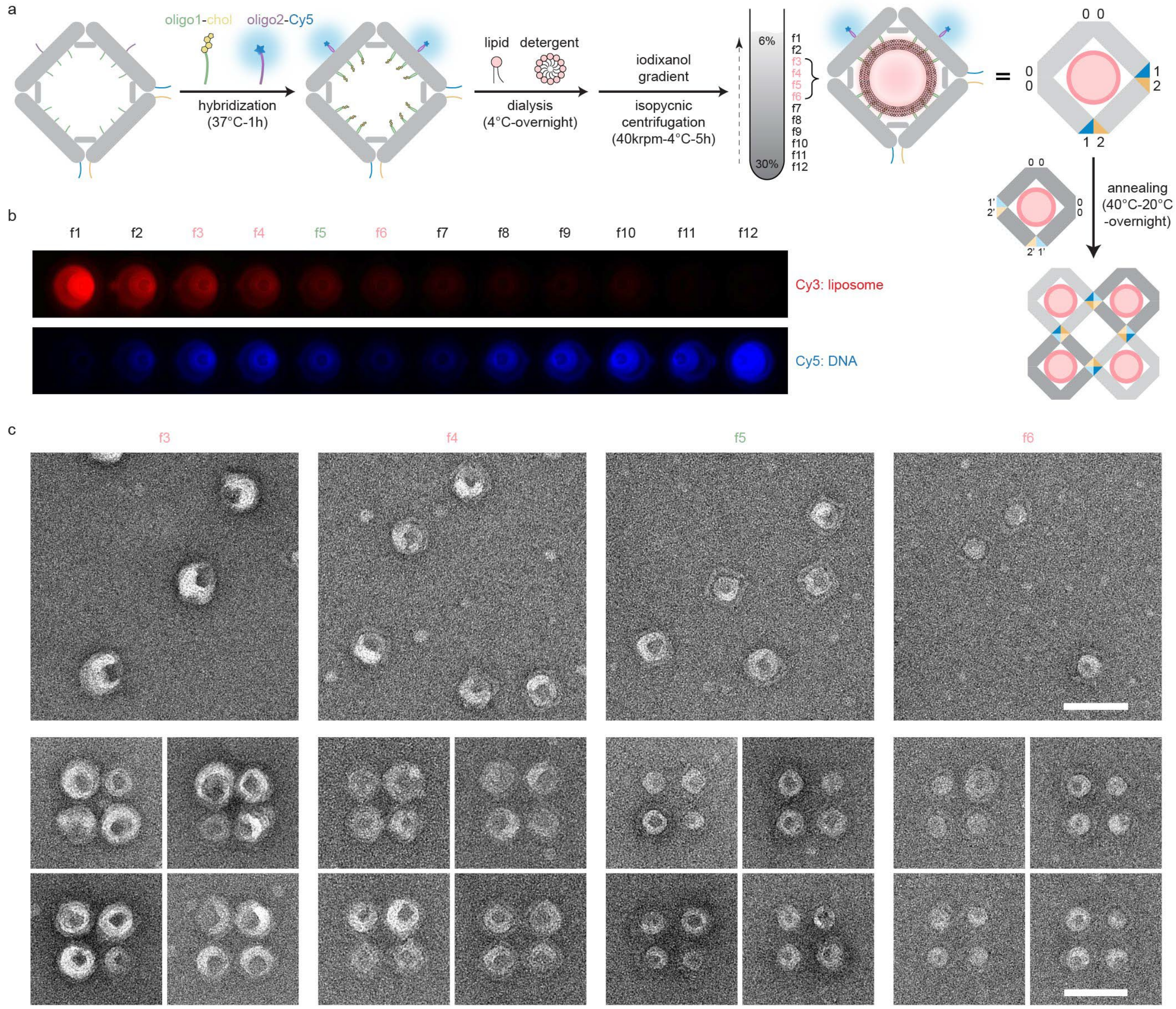

Figure S3. Fractionation of SDO-templated liposomes (STLs). (a) Schematics of STL formation via dialysis-based detergent removal, followed by purification using isopycnic centrifugation, and polymerization into a square-shaped tetramer. See the Methods section in the main text for experimental details. (b) A total of twelve 50 µl fractions were collected into a 96-well plate and scanned using a gel imager (ChemiDoc MP, Bio-rad) with LEDs excitation for Rhodamine (red) and Cy5 (blue). Due to excess lipids in the reaction, most liposomes in the products lacked an origami scaffold and floated to the top fraction (f1). Similarly, a large amount of Cy5-oligos remained in the bottom fractions (f8-f12). Nevertheless, the two fluorescence channels overlapped in fraction 3-6, indicating the presence of STLs. (c) TEM images of STL monomers (top row) in fraction 3-6 respectively, which formed tetramers (bottom row) with another STL variants in the same fraction from a separate preparation. Scale bars: 100 nm.

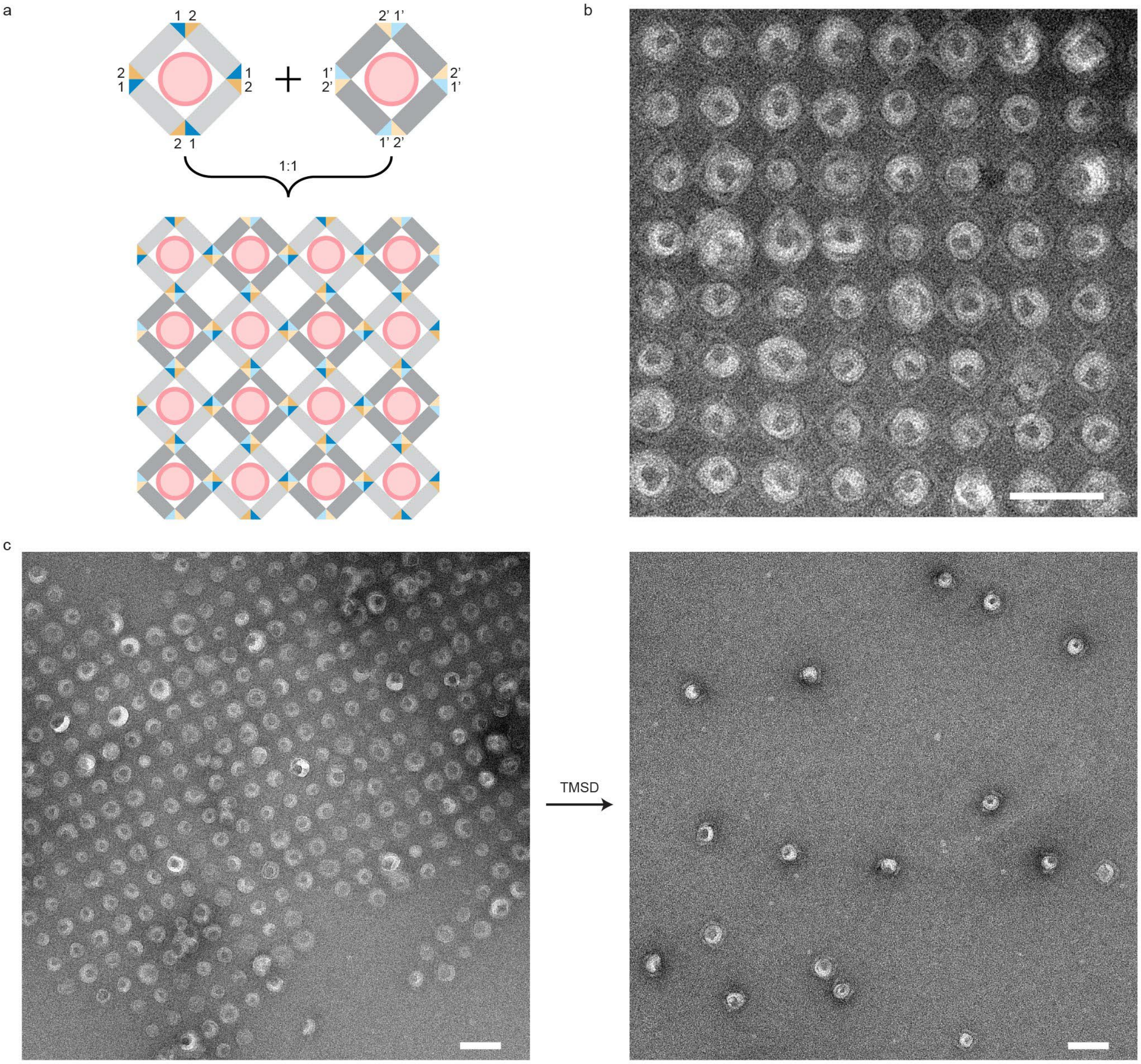

Figure S4. Assembly and disassembly of the STL 2D lattice. (a) Schematic illustration of 2D lattice formation by two equimolar STL variants featuring complementary chiral SEs (1/2 or 2'/1') on all four vertices. (b) A cropped TEM image showing an 8 × 8 liposome array. (c) TEM images confirming the disassembly of STL lattices via toehold-mediated strand displacement (TMSD). Scale bars: 100 nm.

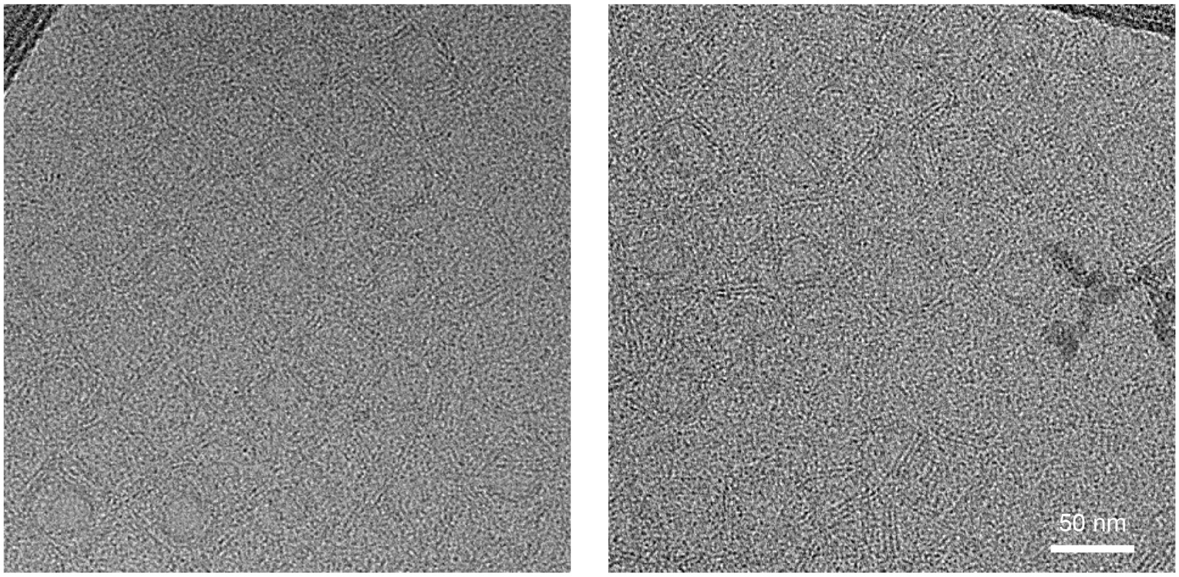

Figure S5. Representative cryo-EM images of 2D liposome arrays. Both SDO and liposome arrays are visible, with the liposomes generally exhibiting a spherical shape and intact membranes. Some vesicles appear absent from the array, likely due to landing in a different focal plane. To more accurately evaluate liposome occupancy, future studies may employ cryo-electron tomography. We note that negative-stain TEM images show a liposome occupancy rate exceeding 98%, which may in fact underestimate the true value given the relatively harsh sample preparation steps, such as washing and drying.

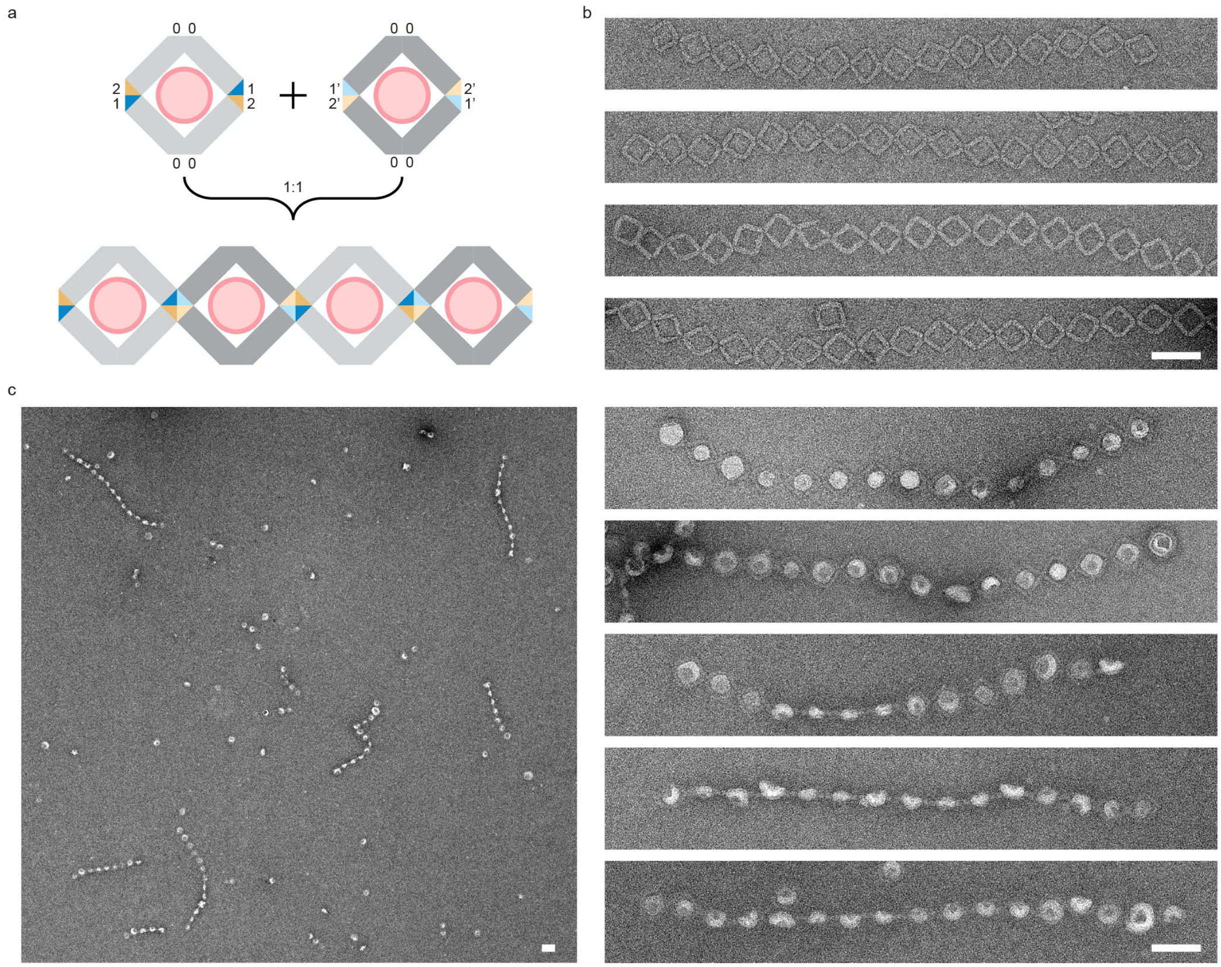

Figure S6. Assembly of the STL 1D array. (a) Schematic illustration of 1D array formation by two equimolar STL variants featuring complementary chiral SEs (1/2 or 2'/1') on a pair of opposite vertices. (b) Cropped TEM images of SDO 1D arrays. (c) A wide-field TEM image (left) and a collection of cropped TEM images (right) showing assembled STL 1D arrays. Scale bars: 100 nm.

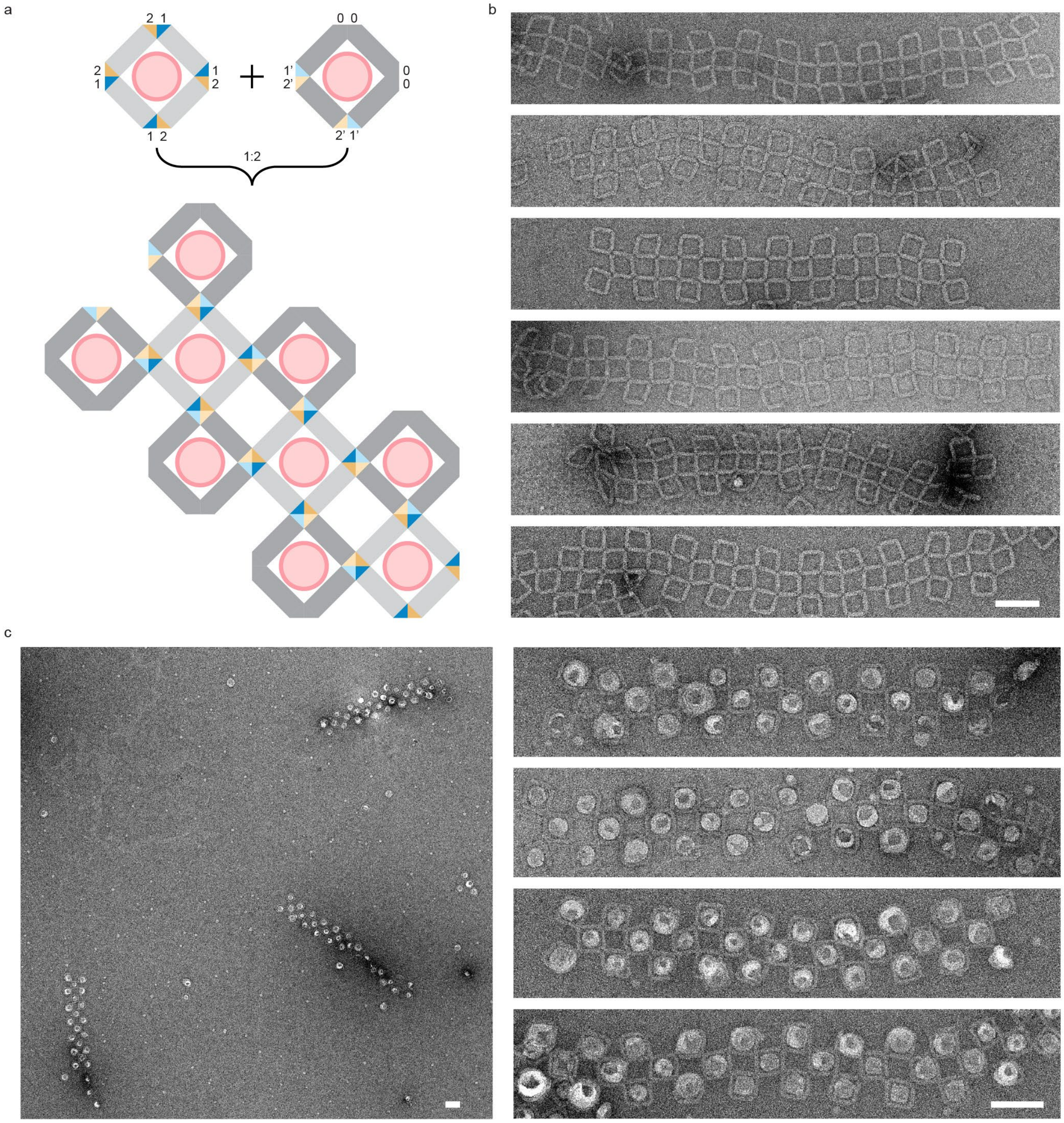

Figure S7. Assembly of the STL three-layer ribbons. (a) Schematic illustration of three-layer ribbon formation by two STL variants in a 1:2 ratio. One variant (LM1) carries chiral SEs (1/2) on all four vertices with alternating chirality, while the other variant (LM2) features complementary SEs (2'/1') on two neighboring vertices with opposite chirality. The precise chirality of SEs ensures correct binding orientation, with a central layer of LM1 sandwiched between two LM2 layers. (b) Cropped TEM images of SDO three-layer ribbons. (c) A wide-field TEM image (left) and a collection of cropped TEM images (right) showing assembled STL three-layer ribbons. Scale bars: 100 nm.

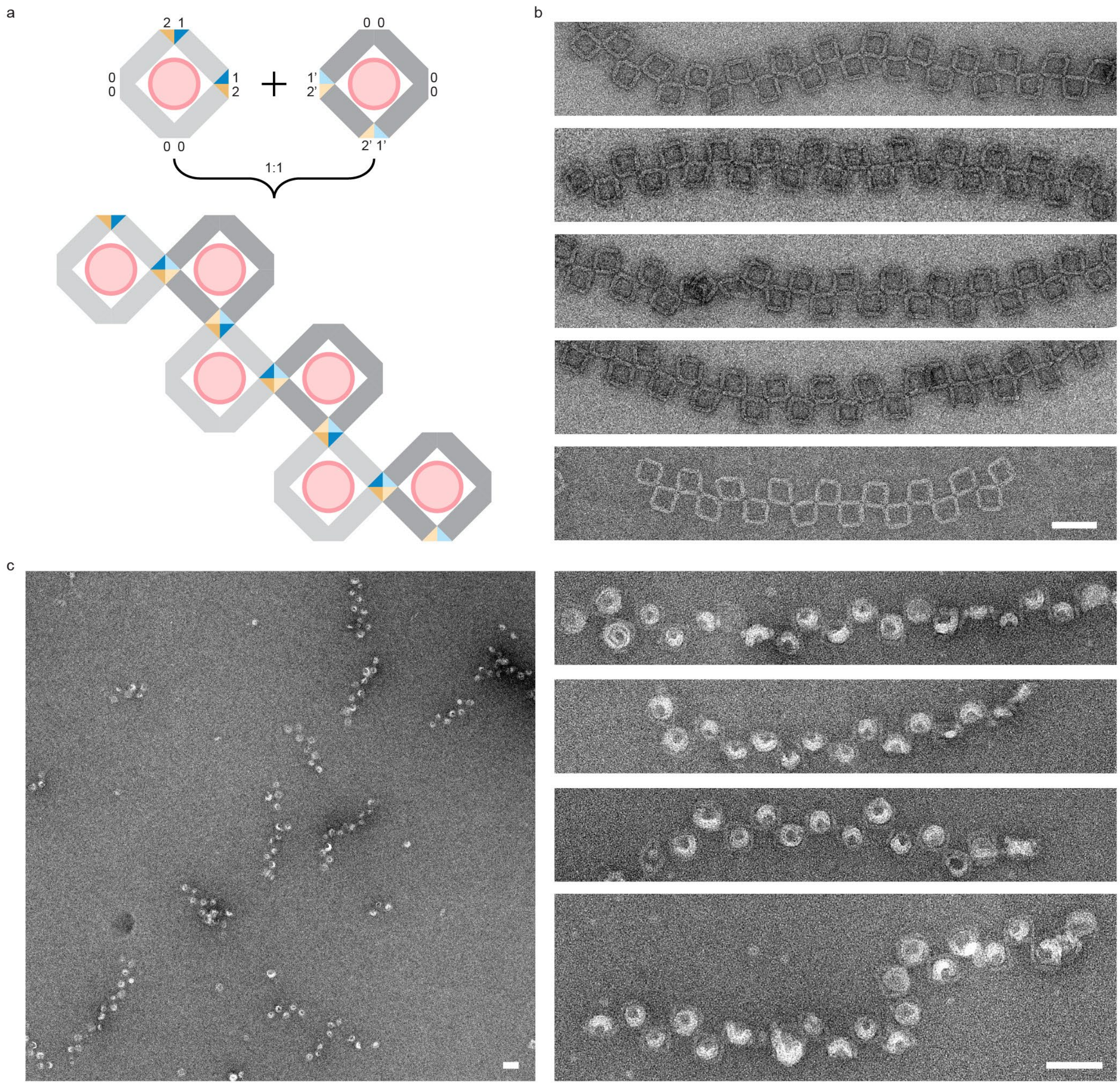

Figure S8. Assembly of STL zigzag array version 1 (v1). (a) Schematic illustration of zigzag array v1 formation by two equimolar STL variants featuring complementary chiral SEs (1/2 or 2'/1') on a pair of neighboring vertices with opposite chirality. The precise chirality of SEs ensures correct binding orientation. (b) Cropped TEM images of SDO zigzag array v1. (c) A wide-field TEM image (left) and a collection of cropped TEM images (right) showing assembled STL zigzag arrays v1. Scale bars: 100 nm.

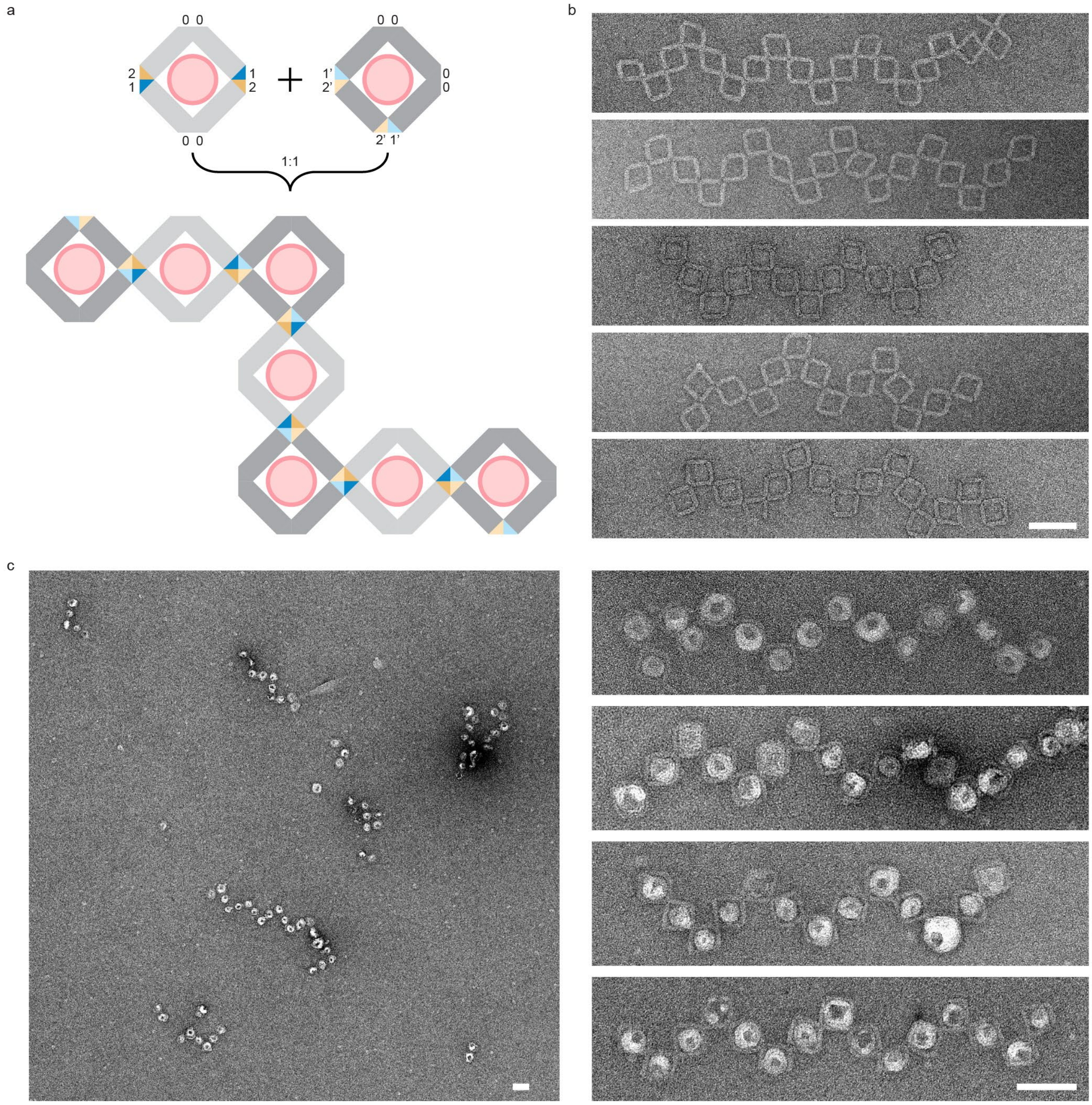

Figure S9. Assembly of STL zigzag array version 2 (v2). (a) Schematic illustration of zigzag array v2 formation by two STL variants in a 1:1 ratio. One variant carries chiral SEs (1/2) on a pair of opposite vertices with the same chirality, while the other variant features complementary SEs (2'/1') on two neighboring vertices with opposite chirality. The precise chirality of SEs ensures correct binding orientation. (b) Cropped TEM images of SDO zigzag array v2. (c) A wide-field TEM image (left) and a collection of cropped TEM images (right) showing assembled STL zigzag arrays v2. Scale bars: 100 nm.

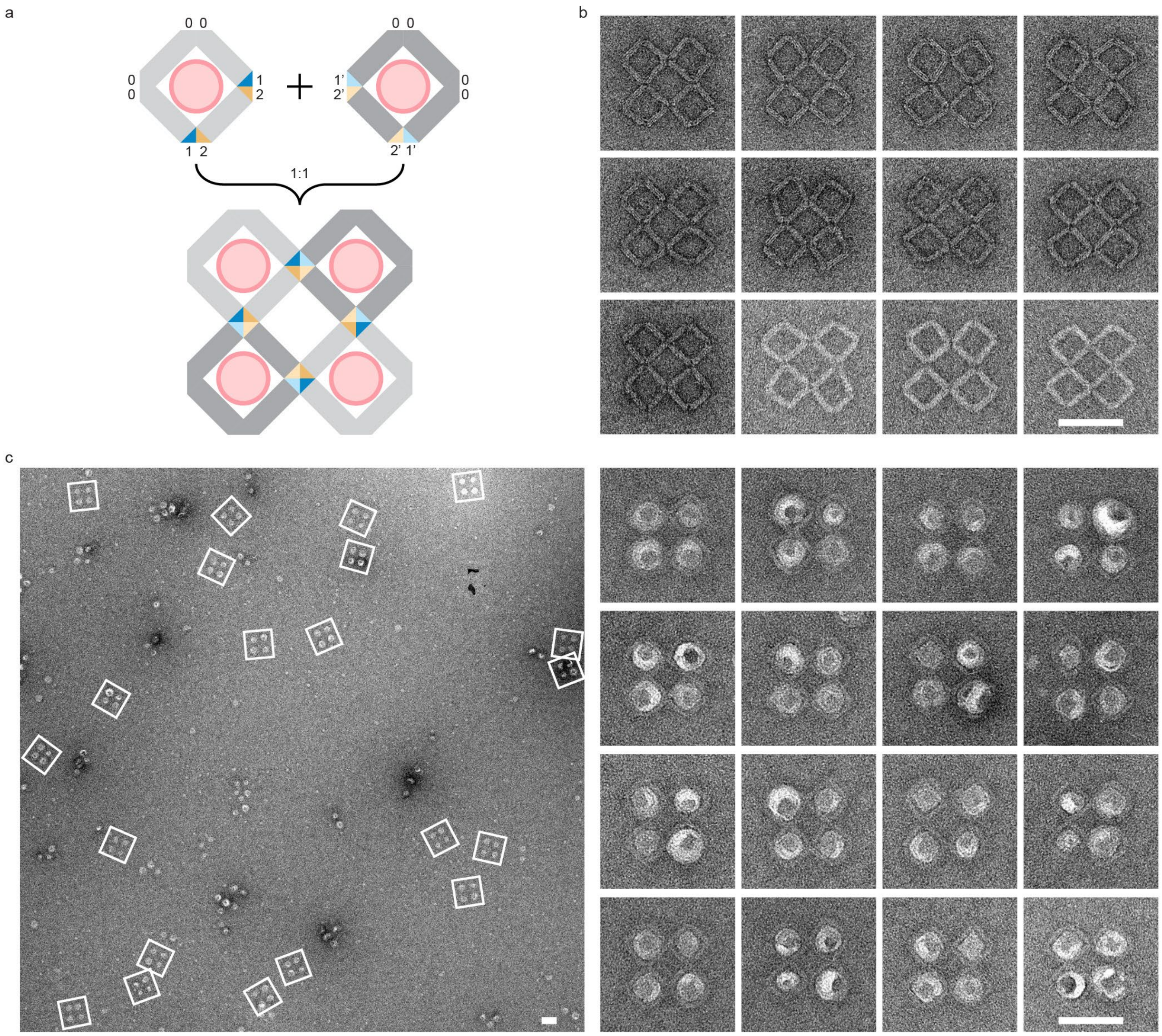

Figure S10. Assembly of STL tetramer version 1 (v1). (a) Schematic illustration of tetramer v1 formation by two equimolar STL variants featuring complementary chiral SEs (1/2 or 2'/1') on a pair of neighboring vertices with opposite chirality. The precise chirality of SEs ensures correct binding orientation. (b) Cropped TEM images of SDO tetramer v1. (c) A wide-field TEM image (left) and a collection of cropped TEM images (right) showing assembled STL tetramer v1. Scale bars: 100 nm.

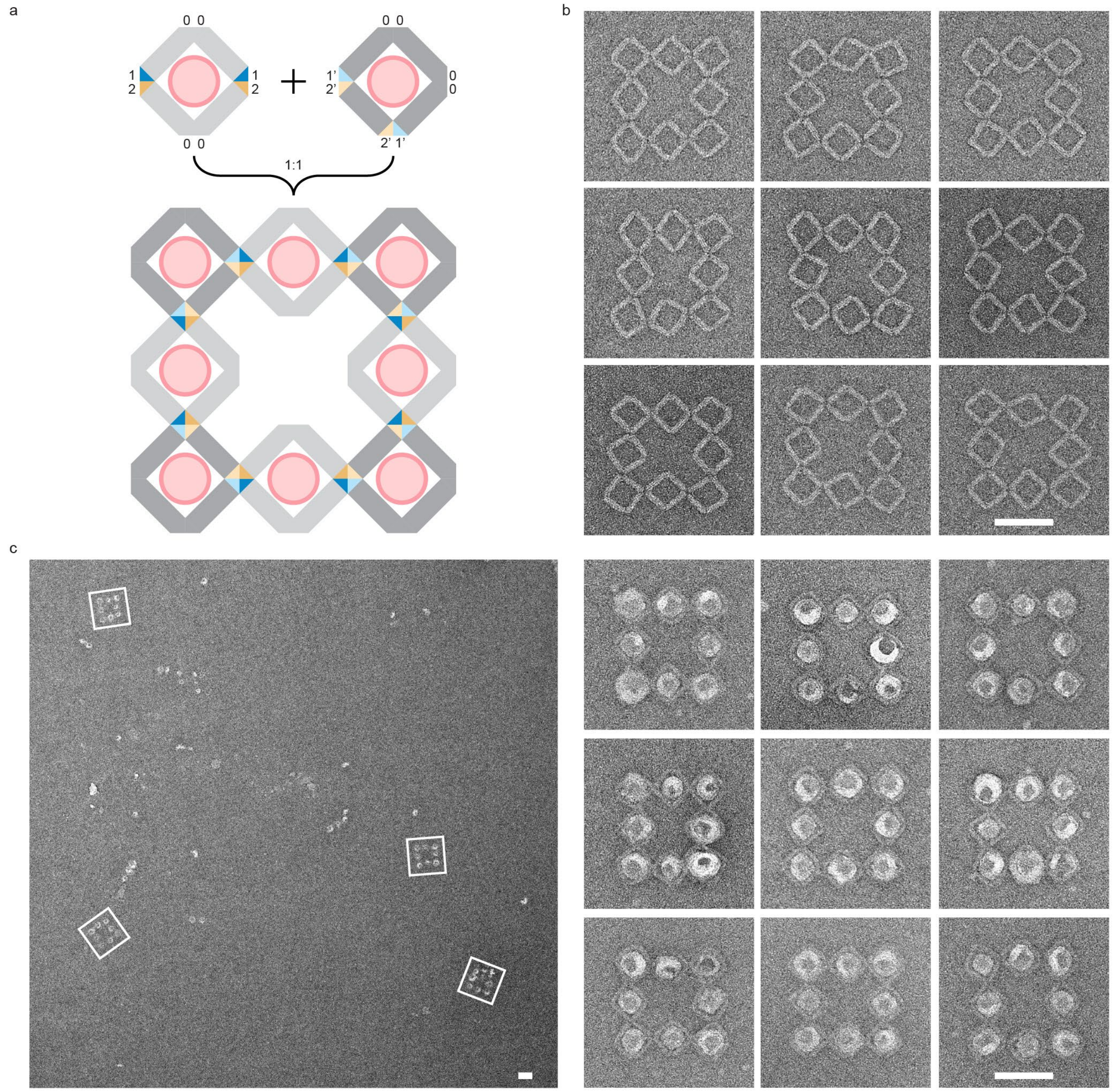

Figure S11. Assembly of STL 8-mer version 1 (v1). (a) Schematic illustration of 8-mer v1 formation by two STL variants in a 1:1 ratio. One variant carries chiral SEs (1/2) on a pair of opposite vertices with opposite chirality, while the other variant features complementary SEs (2'/1') on two neighboring vertices with opposite chirality. The precise chirality of SEs ensures correct binding orientation. (b) Cropped TEM images of SDO 8-mer v1. (c) A wide-field TEM image (left) and a collection of cropped TEM images (right) showing assembled STL 8-mer v1. Scale bars: 100 nm.

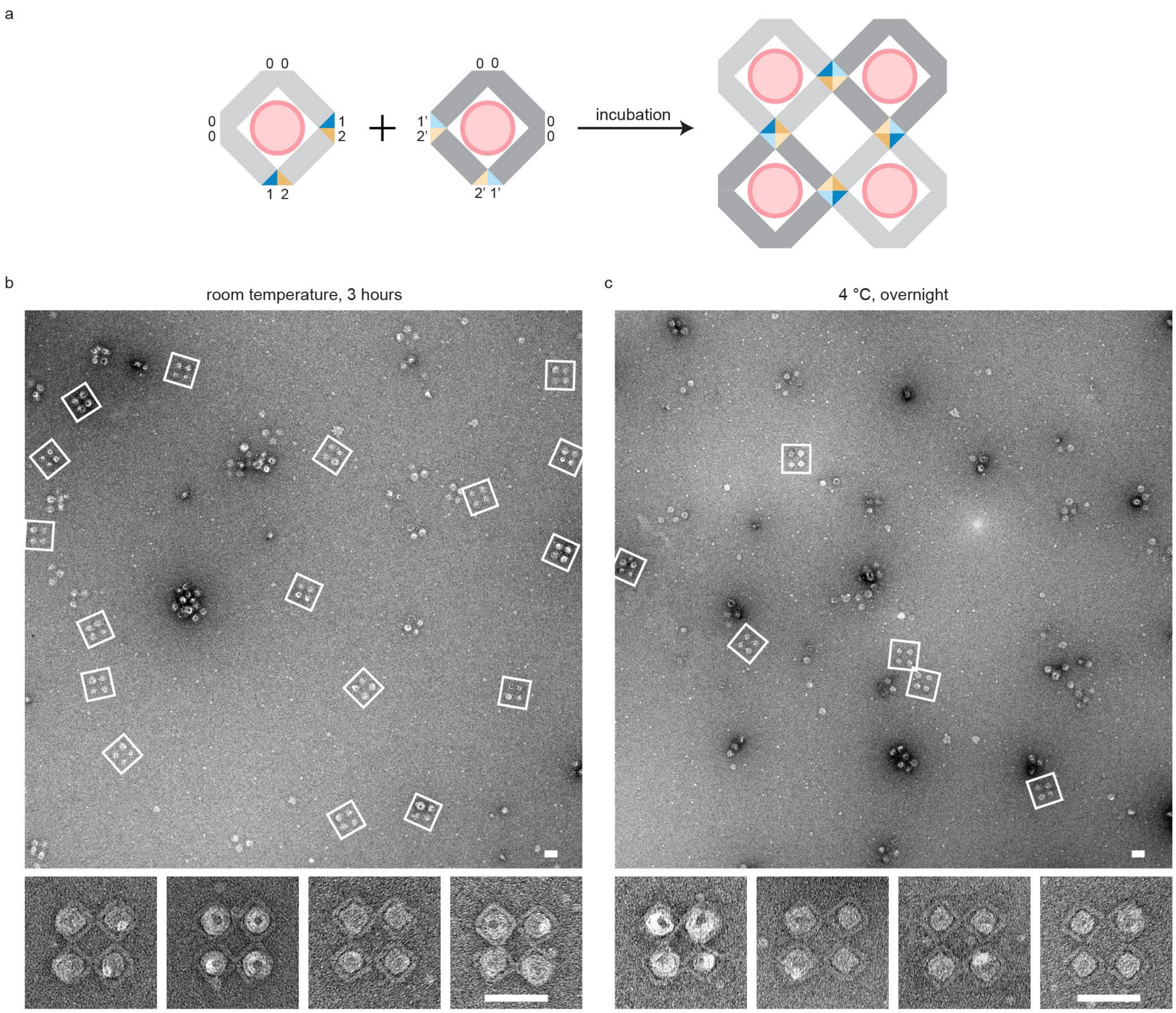

Figure S12. Assembly of STL tetramer v1 by incubation. (a) Schematic illustration of tetramer v1 formation from two equimolar STL variants. (b-c) Wide-field TEM images (top) and a collection of cropped TEM images (bottom) showing assembled STL tetramer v1 after incubation of the STL mixture at room temperature (~23 °C) for 3 hours (b) or 4 °C overnight (c). The yields of correctly formed tetramers were 38% at room temperature and 24% at 4 °C, compared to 38% achieved through the standard annealing from 40 °C to 20 °C (see Table S2 for details). Scale bars: 100 nm.

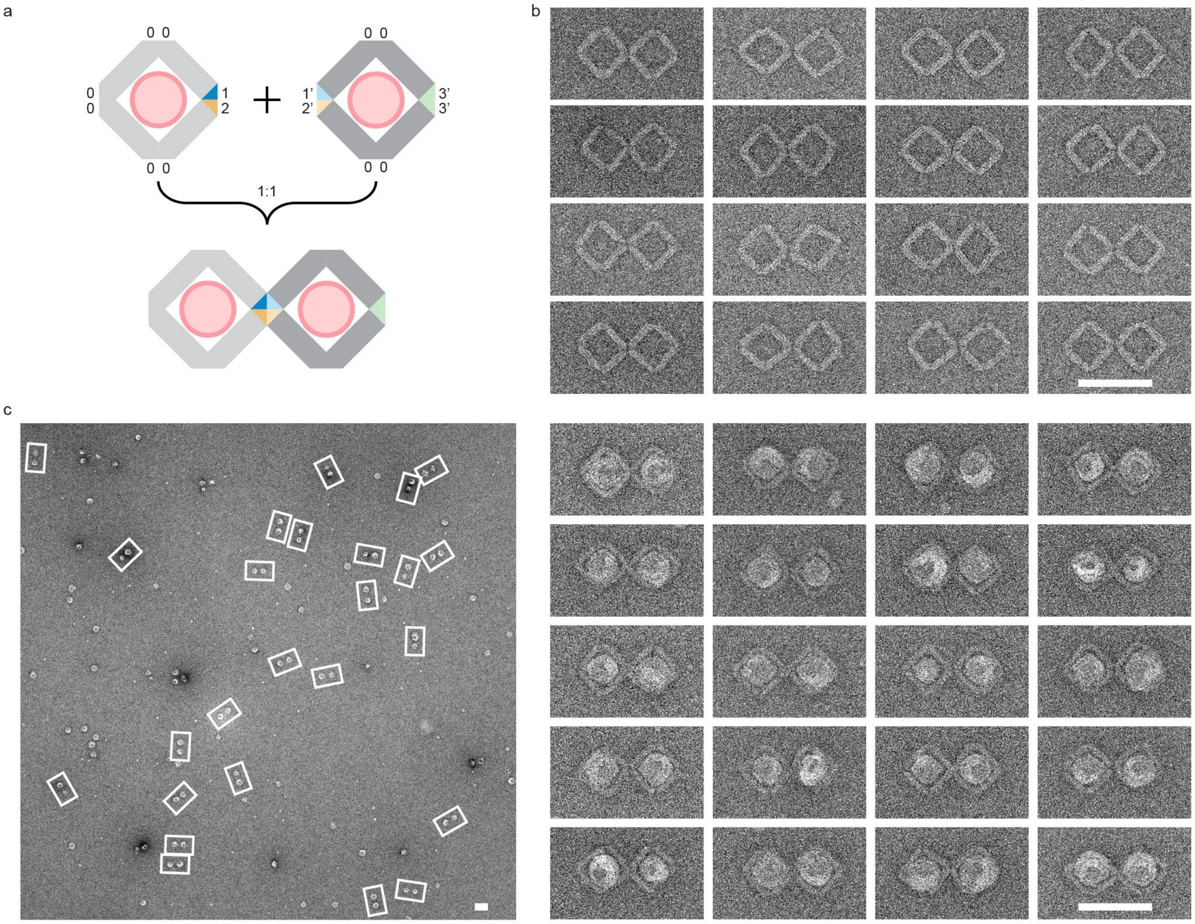

Figure S13. Assembly of the STL dimer. (a) Schematic illustration of dimer formation by two equimolar STL variants featuring complementary chiral SEs (1/2 or 2'/1') on one vertex. (b) Cropped TEM images of SDO dimers. (c) A wide-field TEM image (left) and a collection of cropped TEM images (right) showing assembled STL dimers. Scale bars: 100 nm.

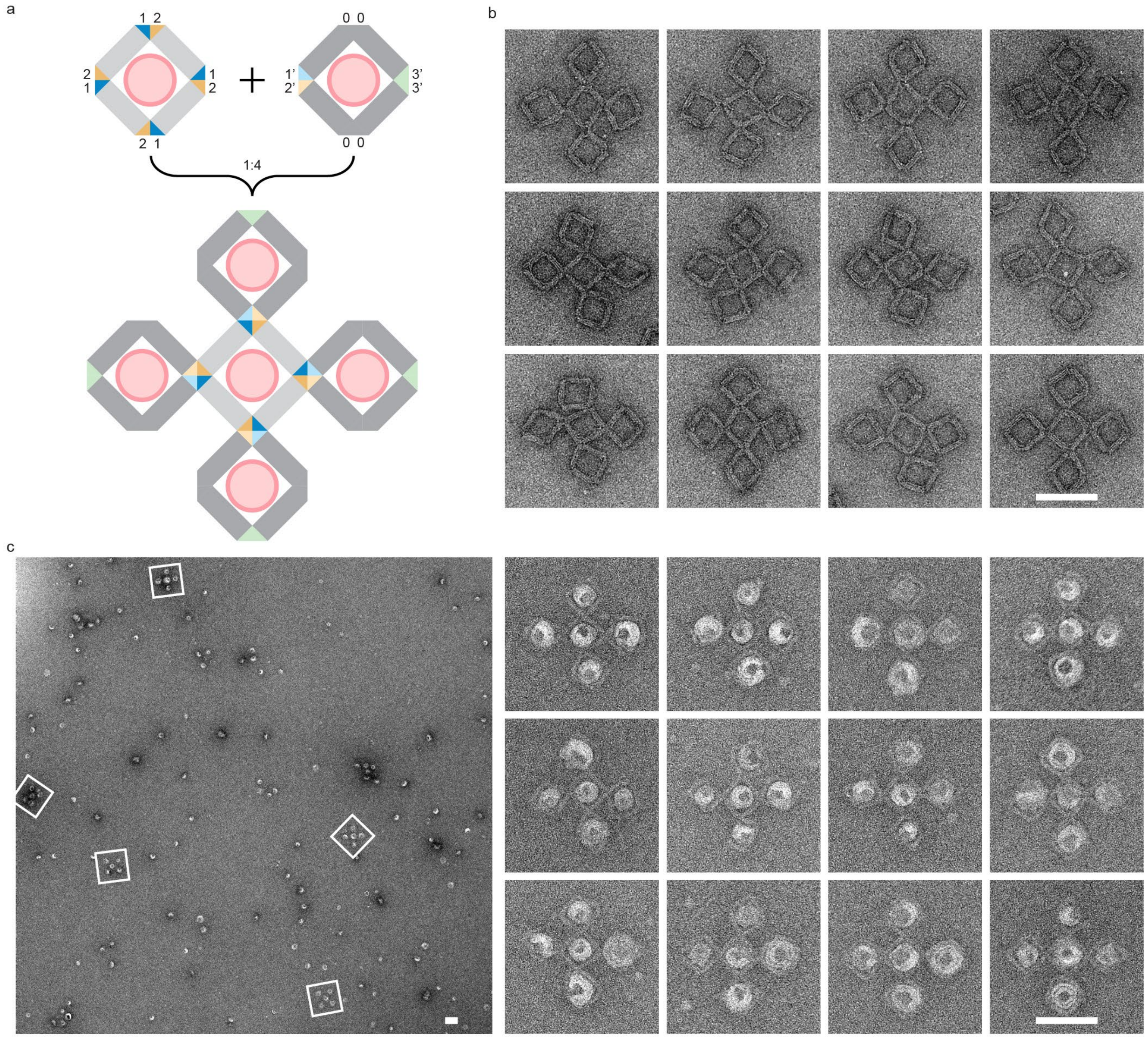

Figure S14. Assembly of STL 5-mer version 1 (v1). (a) Schematic illustration of 5-mer v1 formation by two STL variants in a 1:4 ratio. One variant carries chiral SEs (1/2) on all four vertices, while the other variant features complementary SEs (2'/1') on a single vertex. (b) Cropped TEM images of SDO 5-mer v1. (c) A wide-field TEM image (left) and a collection of cropped TEM images (right) showing assembled STL 5-mer v1. Scale bars: 100 nm.

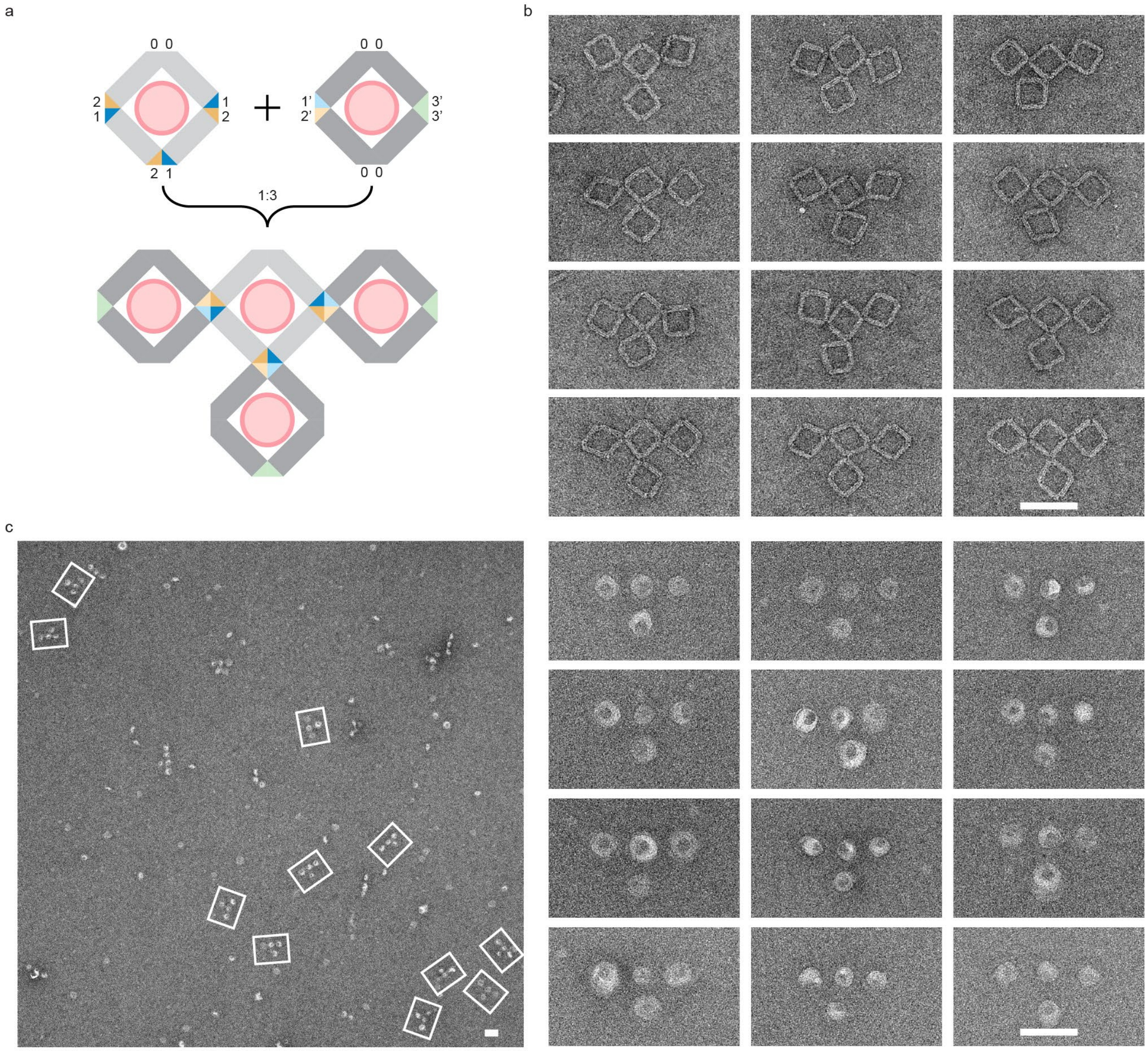

Figure S15. Assembly of STL tetramer version 2 (v2). (a) Schematic illustration of tetramer v2 formation by two STL variants in a 1:3 ratio. One variant carries chiral SEs (1/2) on three vertices, while the other variant features complementary SEs (2'/1') on a single vertex. (b) Cropped TEM images of SDO tetramer v2. (c) A wide-field TEM image (left) and a collection of cropped TEM images (right) showing assembled STL tetramer v2. Scale bars: 100 nm.

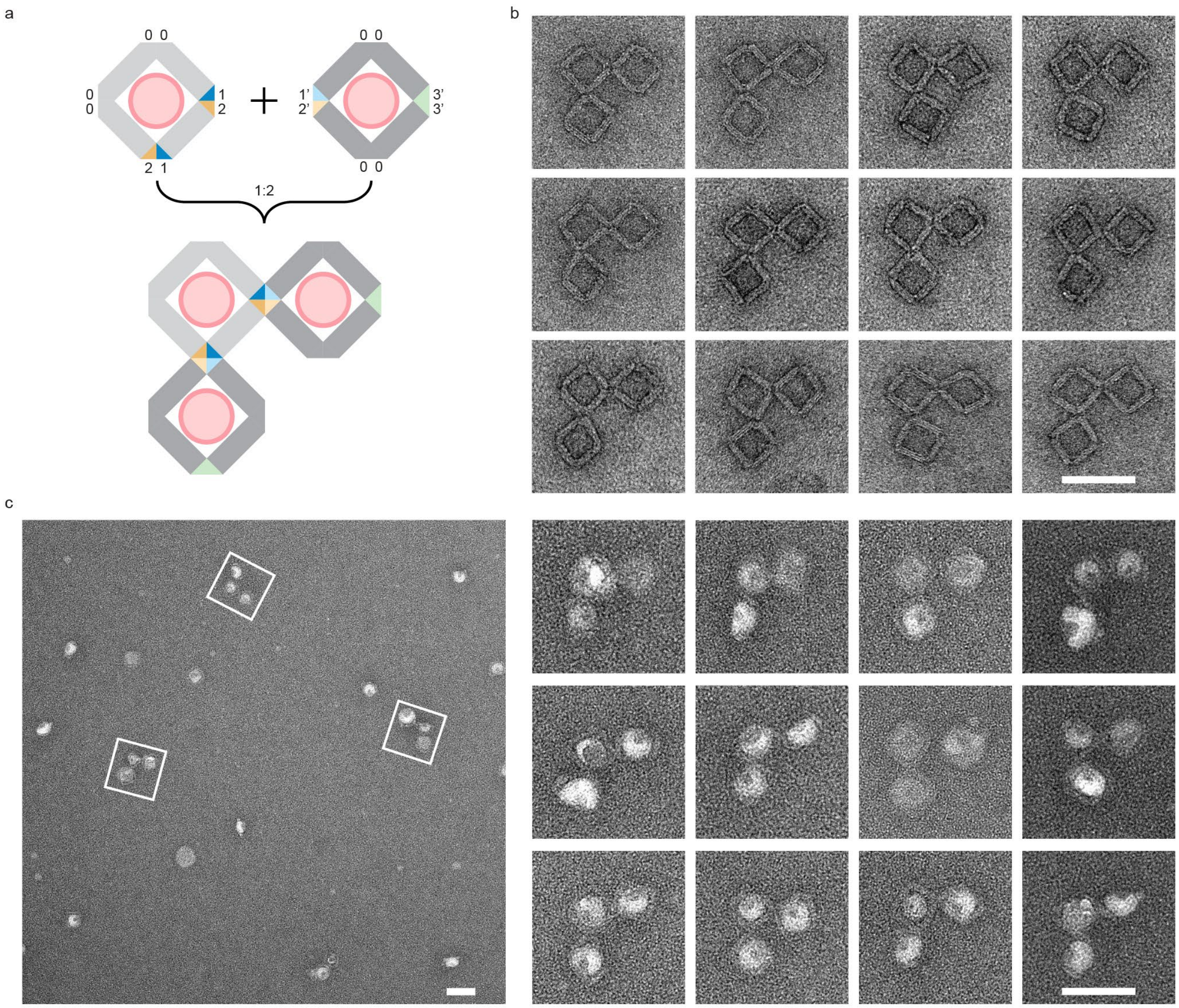

Figure S16. Assembly of STL trimer version 1 (v1). (a) Schematic illustration of trimer v1 formation by two STL variants in a 1:2 ratio. One variant carries chiral SEs (1/2) on two neighboring vertices, while the other variant features complementary SEs (2'/1') on a single vertex. (b) Cropped TEM images of SDO trimer v1. (c) A wide-field TEM image (left) and a collection of cropped TEM images (right) showing assembled STL trimer v1. Scale bars: 100 nm.

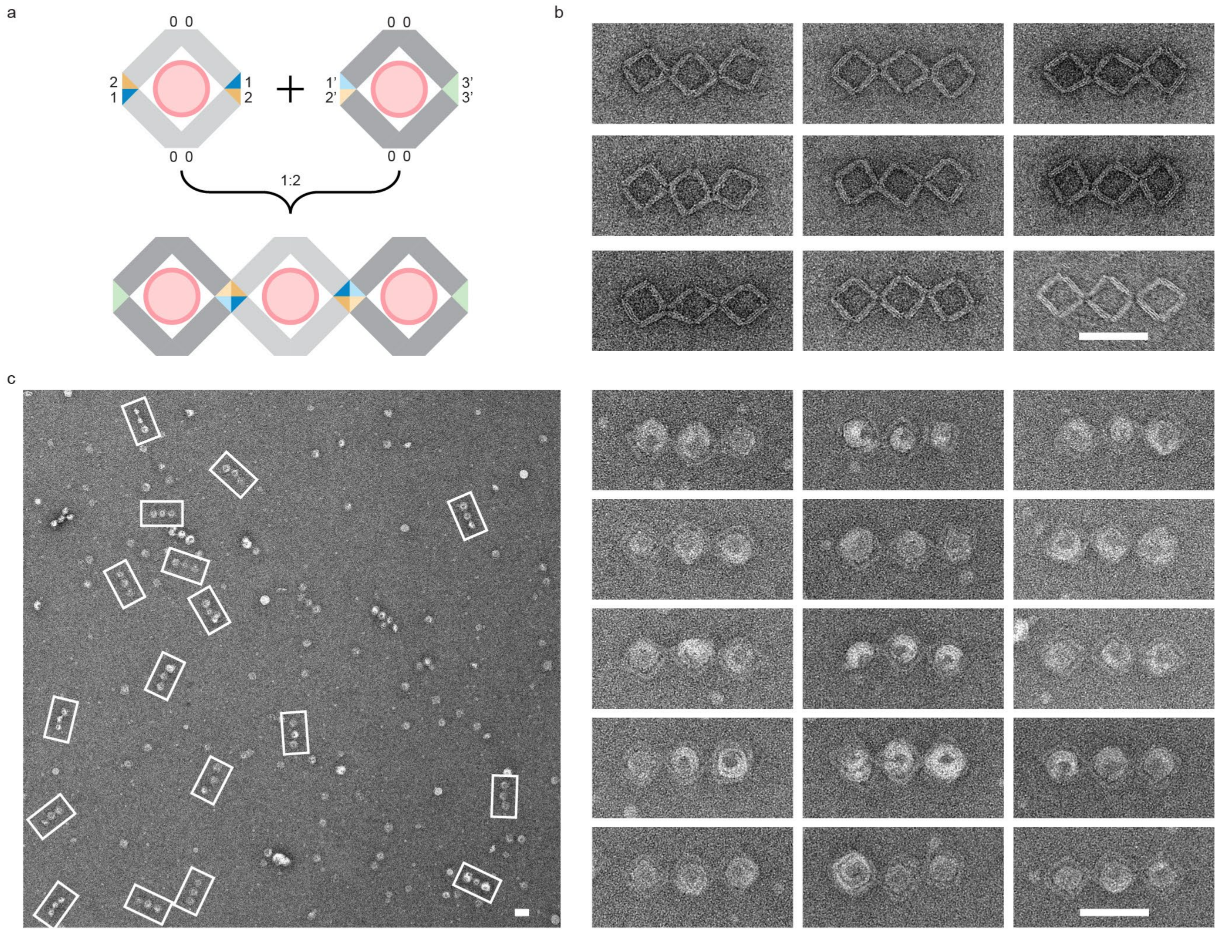

Figure S17. Assembly of STL trimer version 2 (v2). (a) Schematic illustration of trimer v2 formation by two STL variants in a 1:2 ratio. One variant carries chiral SEs (1/2) on two opposite vertices, while the other variant features complementary SEs (2'/1') on a single vertex. (b) Cropped TEM images of SDO trimer v2. (c) A wide-field TEM image (left) and a collection of cropped TEM images (right) showing assembled STL trimer v2. Scale bars: 100 nm.

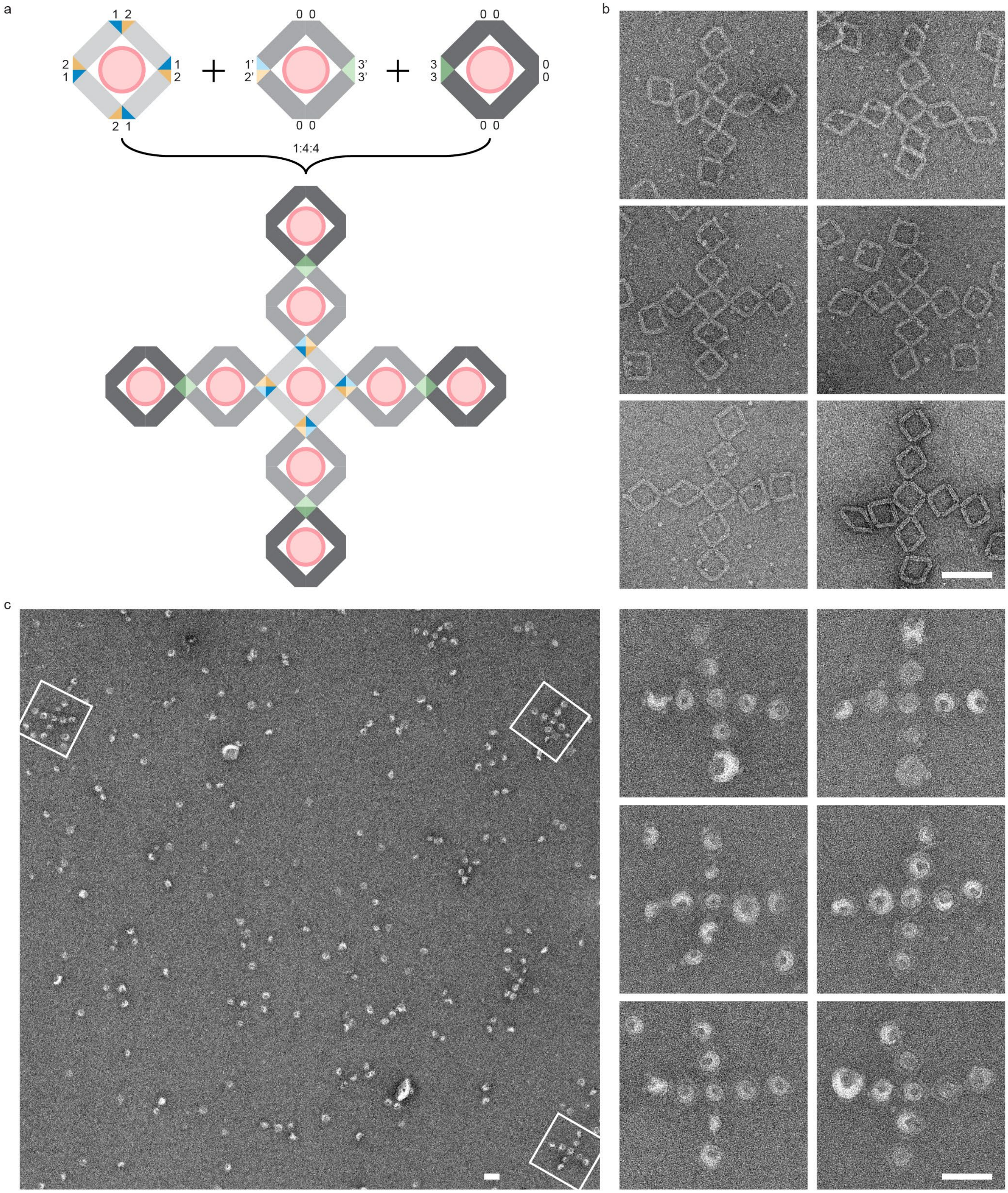

Figure S18. Assembly of STL 9-mer version 1 (v1). (a) Schematic illustration of 9-mer v1 formation by three STL variants in a 1:4:4 ratio. One variant carries chiral SEs (1/2) on all four vertices; the second variant features complementary SEs (2'/1') on one vertex and an additional set of SEs (3') on the opposite vertex; the third variant has complementary SEs (3) on a single vertex. (b) Cropped TEM images of SDO 9-mer v1. (c) A wide-field TEM image (left) and a collection of cropped TEM images (right) showing assembled STL 9-mer v1. Scale bars: 100 nm.

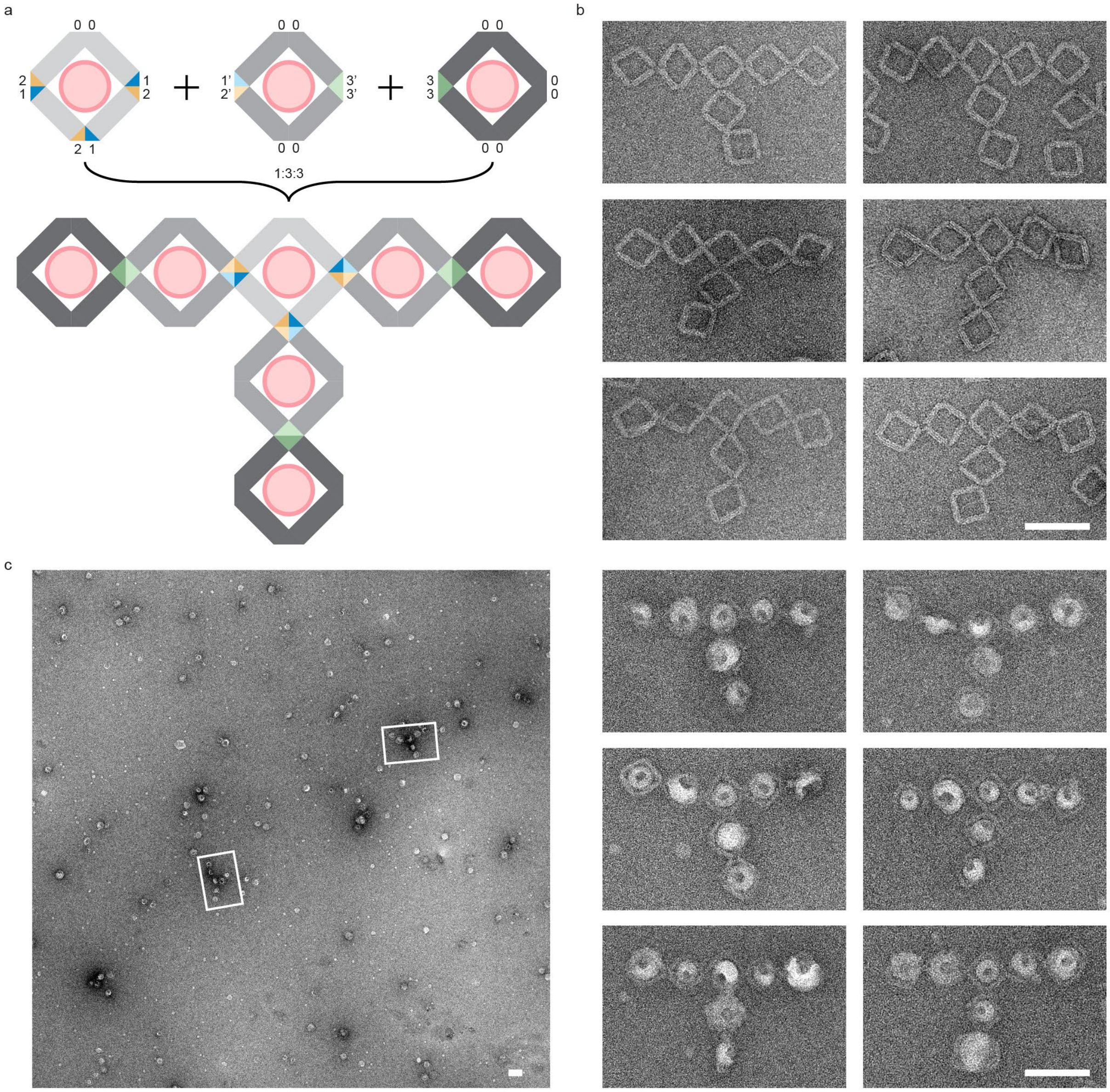

Figure S19. Assembly of STL 7-mer version 1 (v1). (a) Schematic illustration of 7-mer v1 formation by three STL variants in a 1:3:3 ratio. One variant carries chiral SEs (1/2) on three vertices; the second variant features complementary SEs (2'/1') on one vertex and an additional set of SEs (3') on the opposite vertex; the third variant has complementary SEs (3) on a single vertex. (b) Cropped TEM images of SDO 7-mer v1. (c) A wide-field TEM image (left) and a collection of cropped TEM images (right) showing assembled STL 7-mer v1. Scale bars: 100 nm.

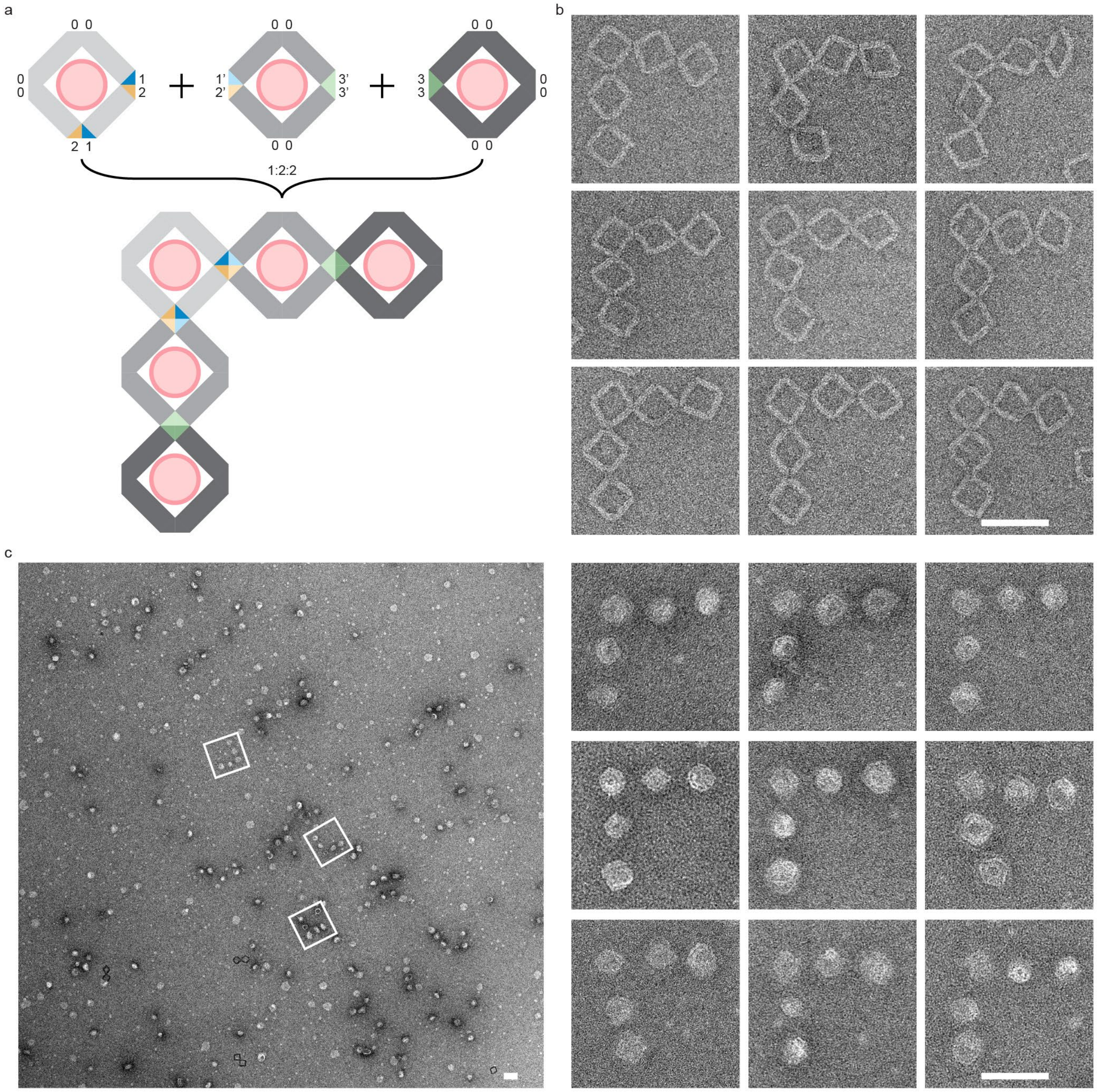

Figure S20. Assembly of STL 5-mer version 2 (v2). (a) Schematic illustration of 5-mer v2 formation by three STL variants in a 1:2:2 ratio. One variant carries chiral SEs (1/2) on two neighboring vertices; the second variant features complementary SEs (2'/1') on one vertex and an additional set of SEs (3') on the opposite vertex; the third variant has complementary SEs (3) on a single vertex. (b) Cropped TEM images of SDO 5-mer v2. (c) A wide-field TEM image (left) and a collection of cropped TEM images (right) showing assembled STL 5-mer v2. Scale bars: 100 nm.

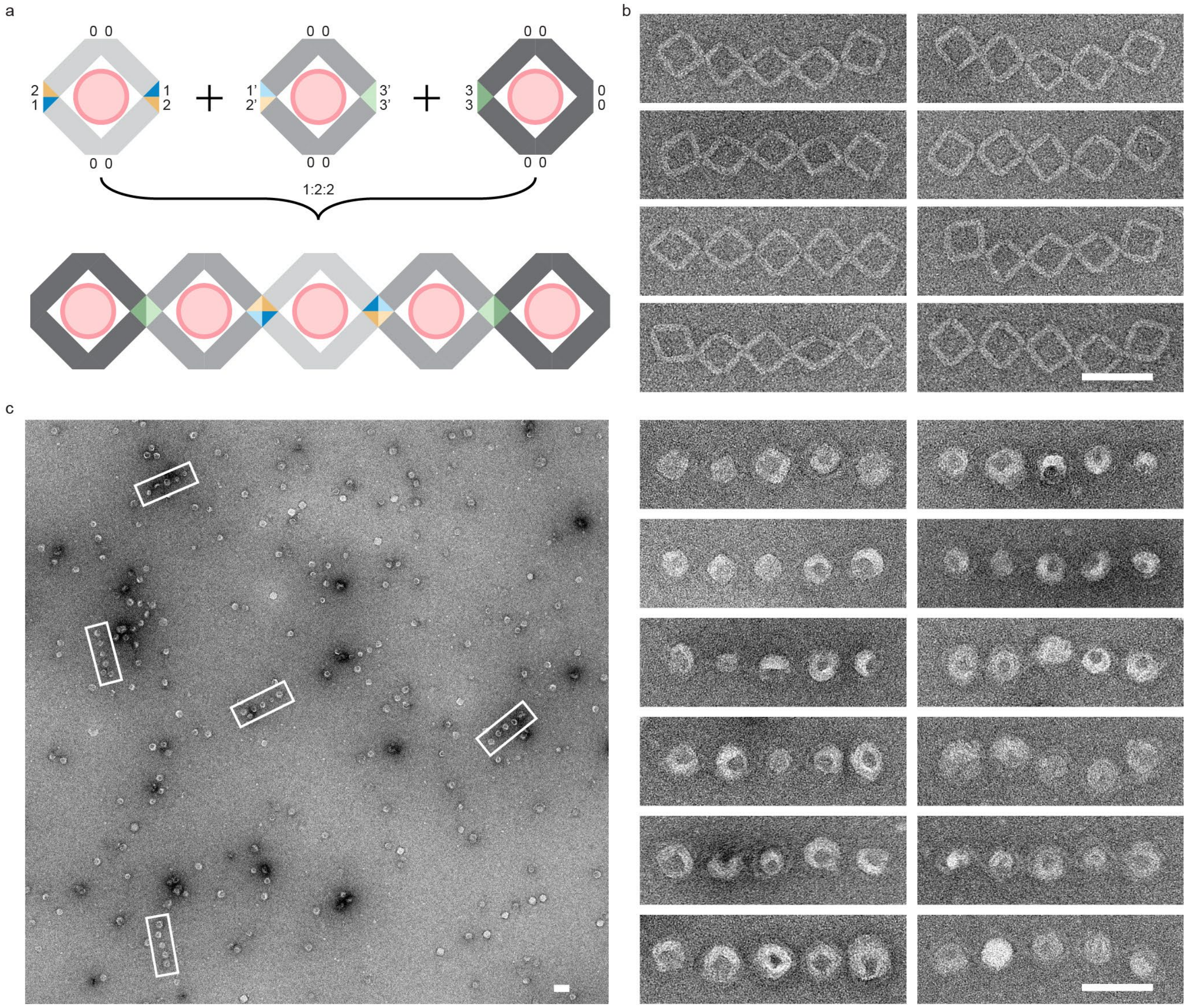

Figure S21. Assembly of STL 5-mer version 3 (v3). (a) Schematic illustration of 5-mer v3 formation by three STL variants in a 1:2:2 ratio. One variant carries chiral SEs (1/2) on two opposite vertices; the second variant features complementary SEs (2'/1') on one vertex and an additional set of SEs (3') on the opposite vertex; the third variant has complementary SEs (3) on a single vertex. (b) Cropped TEM images of SDO 5-mer v3. (c) A wide-field TEM image (left) and a collection of cropped TEM images (right) showing assembled STL 5-mer v3. Scale bars: 100 nm.

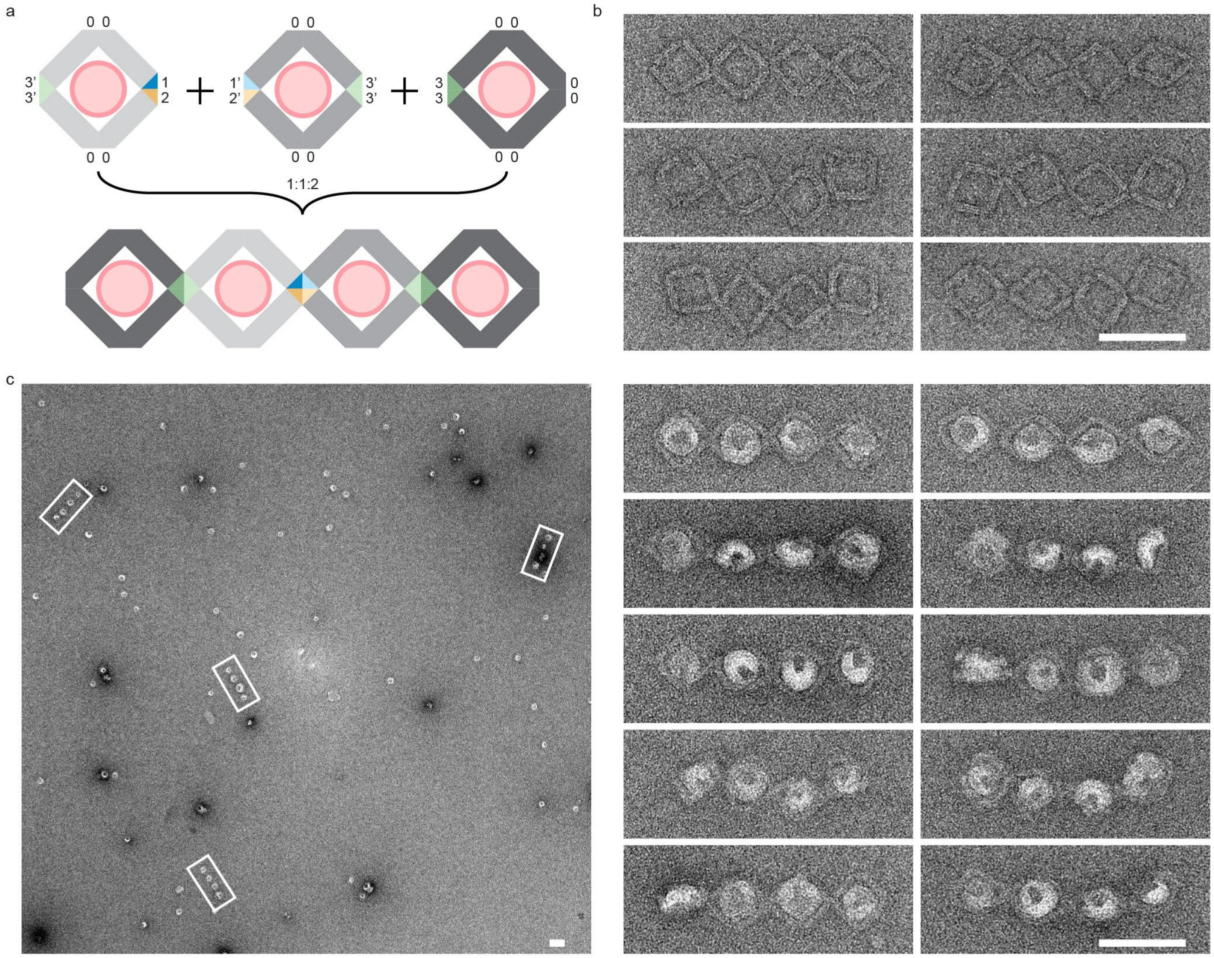

Figure S22. Assembly of STL tetramer version 3 (v3). (a) Schematic illustration of tetramer v3 formation by three STL variants in a 1:1:2 ratio. One variant carries chiral SEs (1/2) on one vertex and an additional set of SEs (3') on the opposite vertex; the second variant features complementary SEs (2'/1') on one vertex and an additional set of SEs (3') on the opposite vertex; the third variant has complementary SEs (3) on a single vertex. (b) Cropped TEM images of SDO tetramer v3. (c) A wide-field TEM image (left) and a collection of cropped TEM images (right) showing assembled STL tetramer v3. Scale bars: 100 nm.

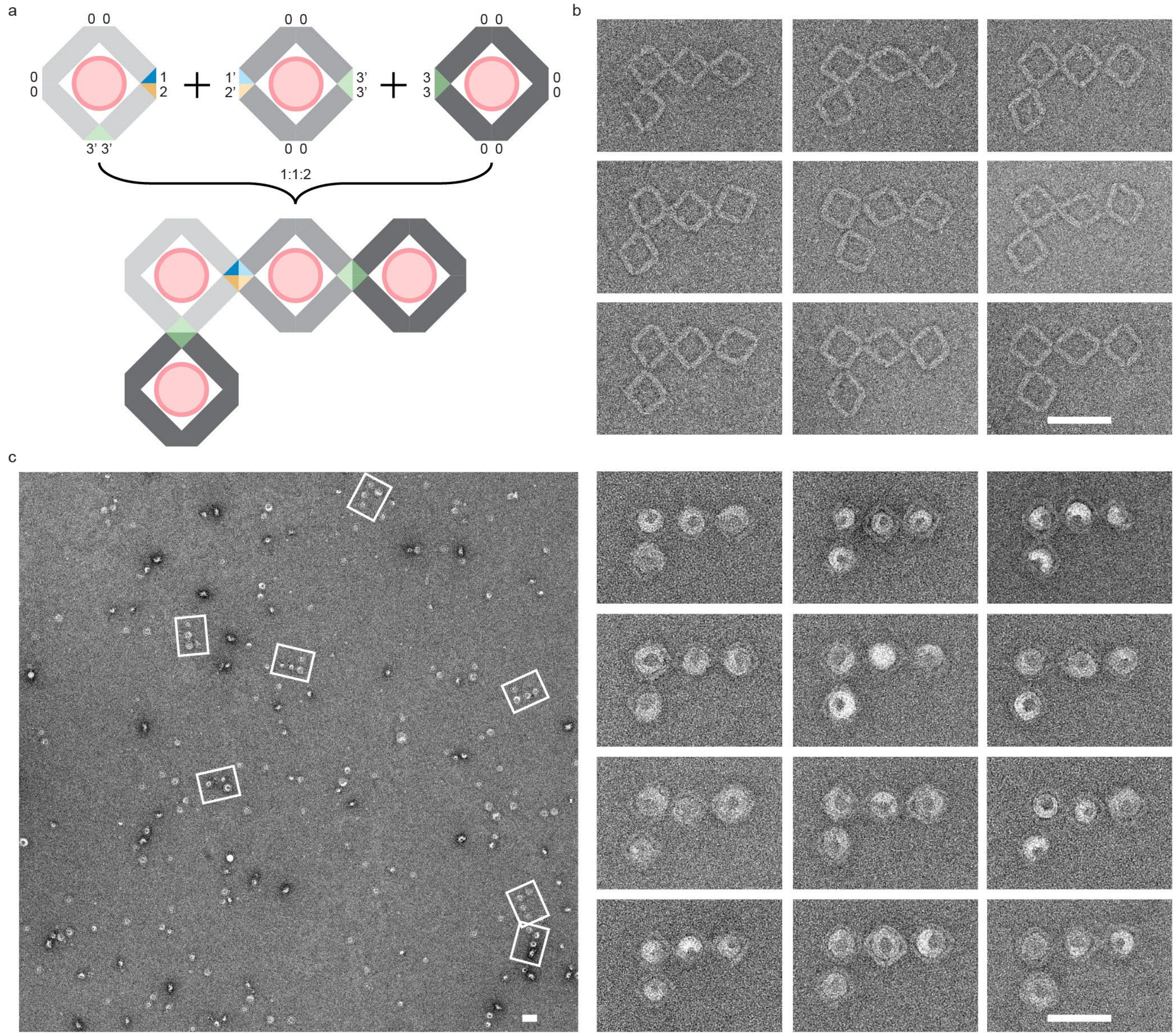

Figure S23. Assembly of STL tetramer version 4 (v4). (a) Schematic illustration of tetramer v4 formation by three STL variants in a 1:1:2 ratio. One variant carries chiral SEs (1/2) on one vertex and an additional set of SEs (3') on the neighboring vertex; the second variant features complementary SEs (2'/1') on one vertex and an additional set of SEs (3') on the opposite vertex; the third variant has complementary SEs (3) on a single vertex. (b) Cropped TEM images of SDO tetramer v4. (c) A wide-field TEM image (left) and a collection of cropped TEM images (right) showing assembled STL tetramer v4. Scale bars: 100 nm.

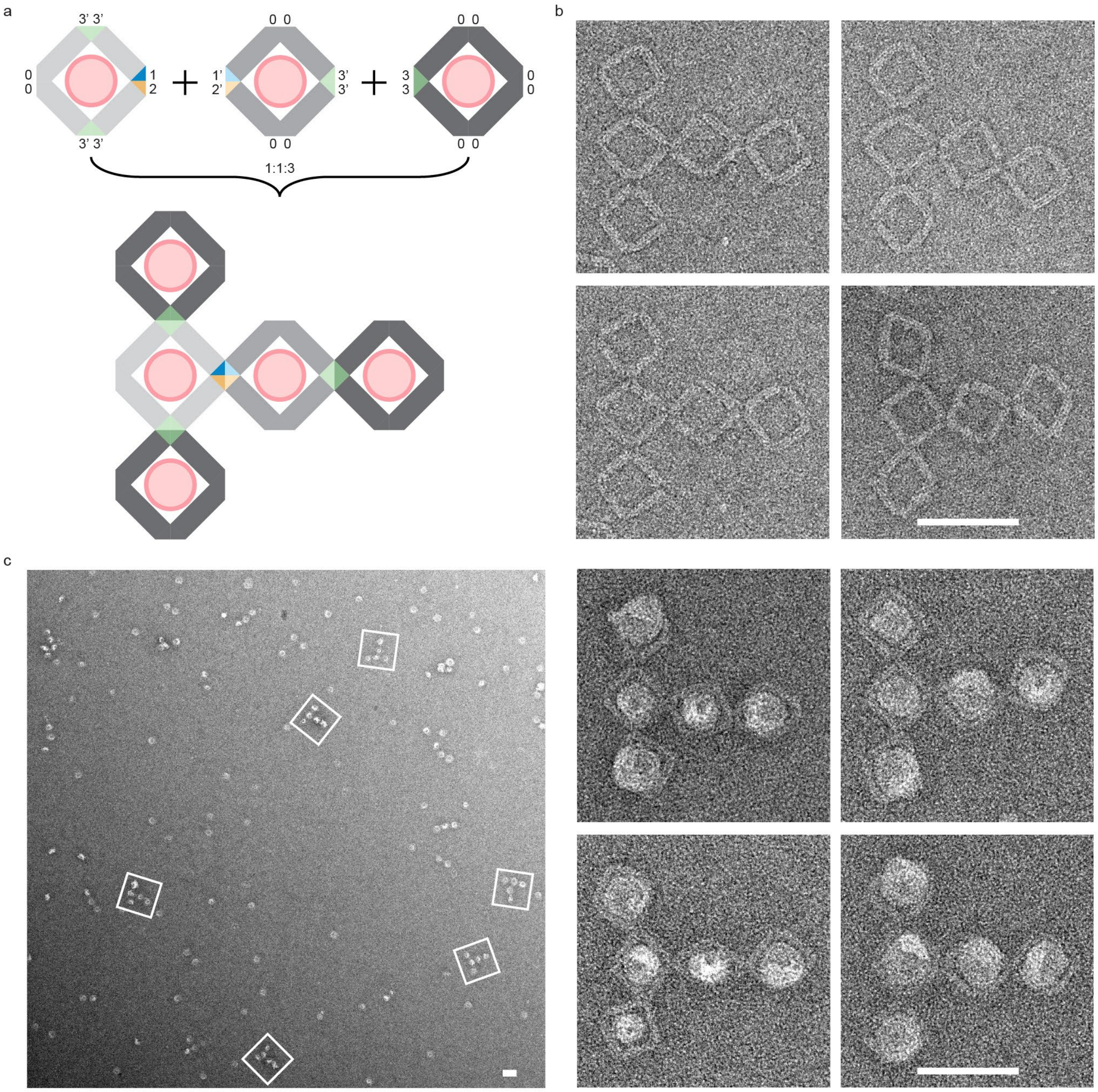

Figure S24. Assembly of STL 5-mer version 4 (v4). (a) Schematic illustration of 5-mer v4 formation by three STL variants in a 1:1:3 ratio. One variant carries chiral SEs (1/2) on one vertex and an additional set of SEs (3') on two neighboring vertices; the second variant features complementary SEs (2'/1') on one vertex and an additional set of SEs (3') on the opposite vertex; the third variant has complementary SEs (3) on a single vertex. (b) Cropped TEM images of SDO 5-mer v4. (c) A wide-field TEM image (left) and a collection of cropped TEM images (right) showing assembled STL 5-mer v4. Scale bars: 100 nm.

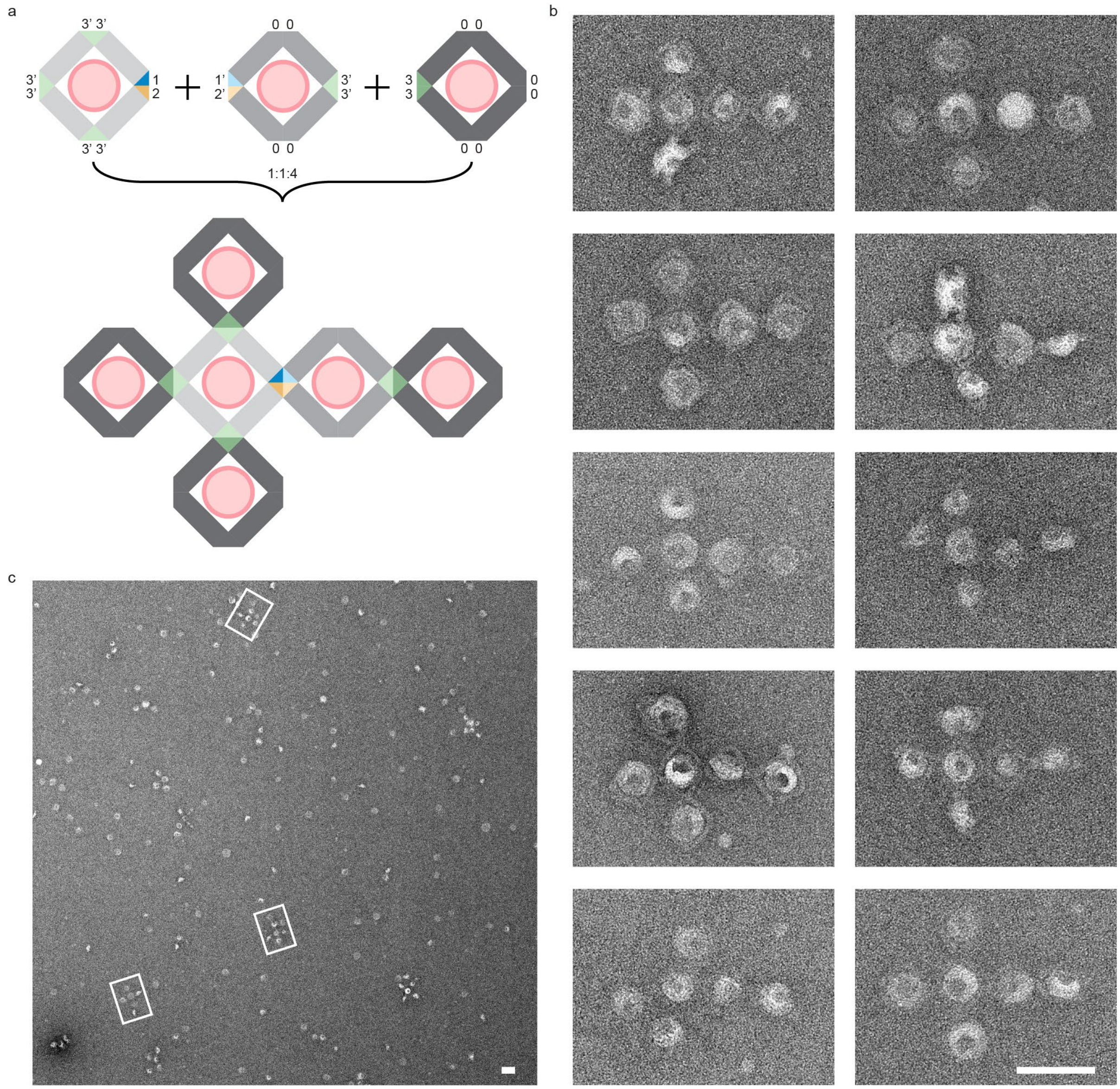

Figure S25. Assembly of STL 6-mer version 1 (v1). (a) Schematic illustration of 6-mer v1 formation by three STL variants in a 1:1:4 ratio. One variant carries chiral SEs (1/2) on one vertex and an additional set of SEs (3') on the other three vertices; the second variant features complementary SEs (2'/1') on one vertex and an additional set of SEs (3') on the opposite vertex; the third variant has complementary SEs (3) on a single vertex. (b) Cropped TEM images of SDO 6-mer v1. (c) A wide-field TEM image (left) and a collection of cropped TEM images (right) showing assembled STL 6-mer v1. Scale bars: 100 nm.

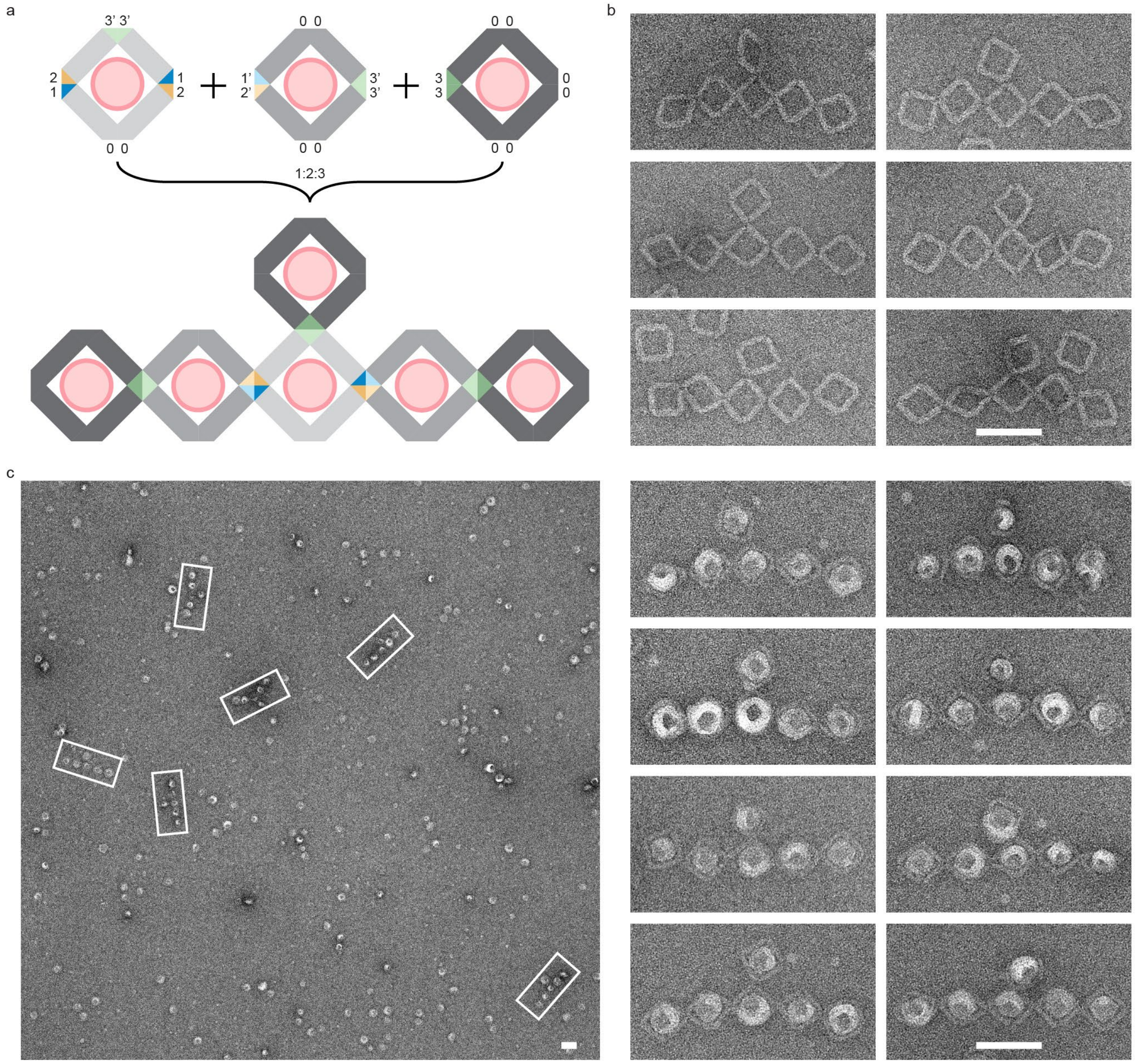

Figure S26. Assembly of STL 6-mer version 2 (v2). (a) Schematic illustration of 6-mer v2 formation by three STL variants in a 1:2:3 ratio. One variant carries chiral SEs (1/2) on two opposite vertices and an additional set of SEs (3') on another vertex; the second variant features complementary SEs (2'/1') on one vertex and an additional set of SEs (3') on the opposite vertex; the third variant has complementary SEs (3) on a single vertex. (b) Cropped TEM images of SDO 6-mer v2. (c) A wide-field TEM image (left) and a collection of cropped TEM images (right) showing assembled STL 6-mer v2. Scale bars: 100 nm.

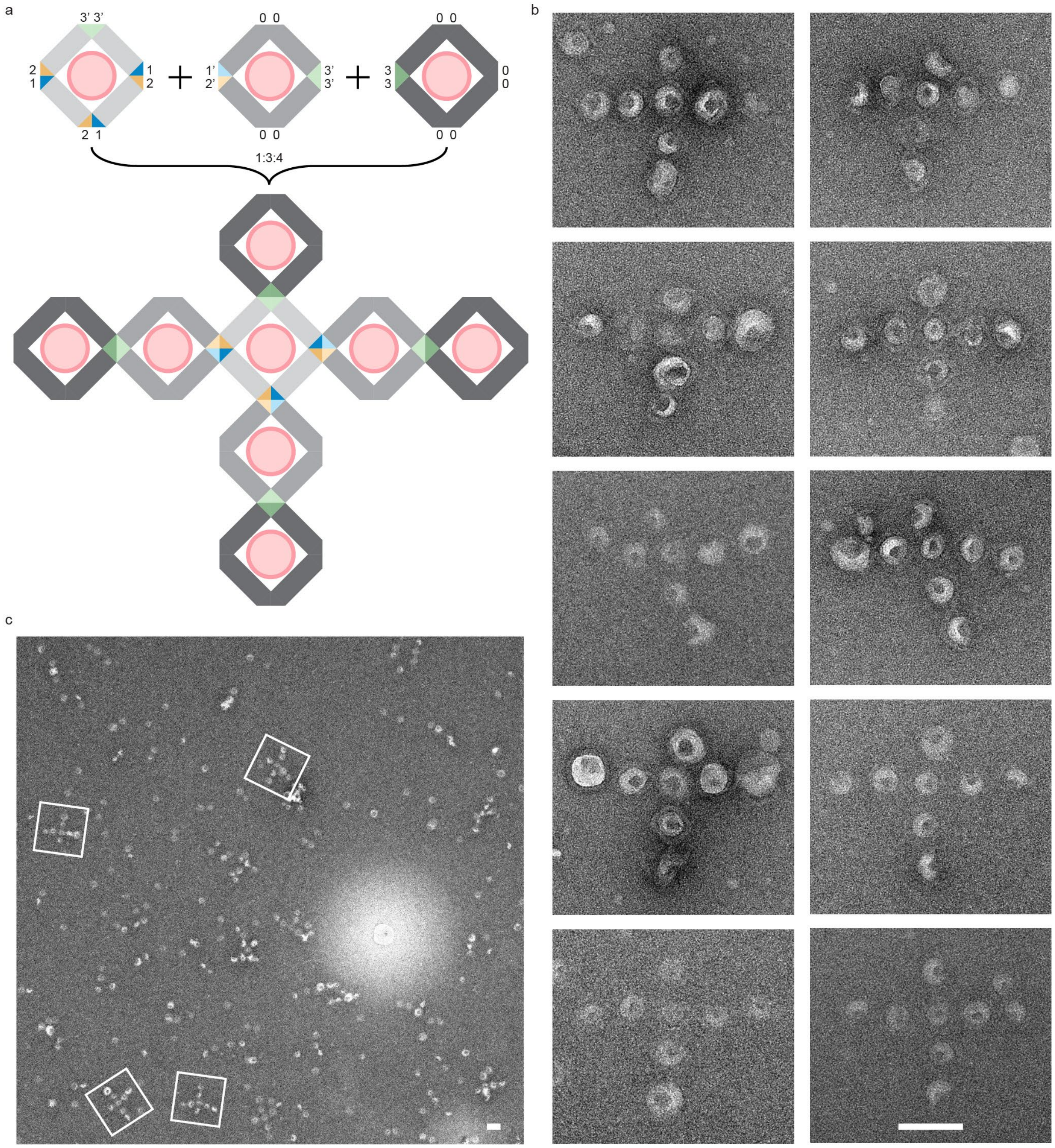

Figure S27. Assembly of STL 8-mer version 2 (v2). (a) Schematic illustration of 8-mer v2 formation by three STL variants in a 1:3:4 ratio. One variant carries chiral SEs (1/2) on three vertices and an additional set of SEs (3') on the last vertex; the second variant features complementary SEs (2'/1') on one vertex and an additional set of SEs (3') on the opposite vertex; the third variant has complementary SEs (3) on a single vertex. (b) Cropped TEM images of SDO 8-mer v2. (c) A wide-field TEM image (left) and a collection of cropped TEM images (right) showing assembled STL 8-mer v2. Scale bars: 100 nm.

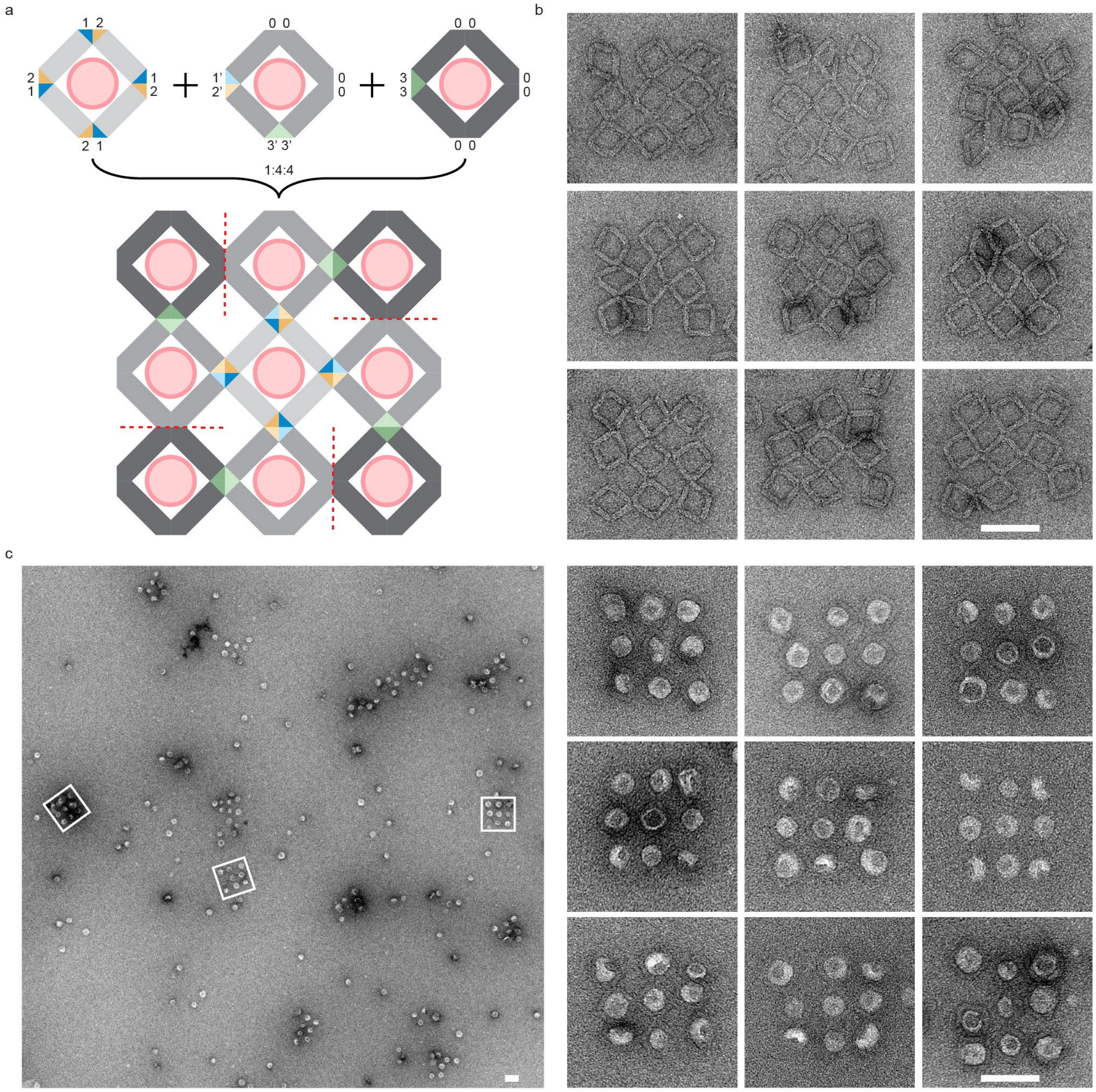

Figure S28. Assembly of STL 9-mer version 2 (v2). (a) Schematic illustration of 9-mer v2 formation by three STL variants in a 1:4:4 ratio. One variant carries chiral SEs (1/2) on all four vertices; the second variant features complementary SEs (2'/1') on one vertex and an additional set of SEs (3') on the neighboring vertex; the third variant has complementary SEs (3) on a single vertex. The precise chirality of SEs ensures correct binding orientation. Dashed lines indicate no sticky end pairing between two monomers. (b) Cropped TEM images of SDO 9-mer v2. (c) A wide-field TEM image (left) and a collection of cropped TEM images (right) showing assembled STL 9-mer v2. Scale bars: 100 nm.

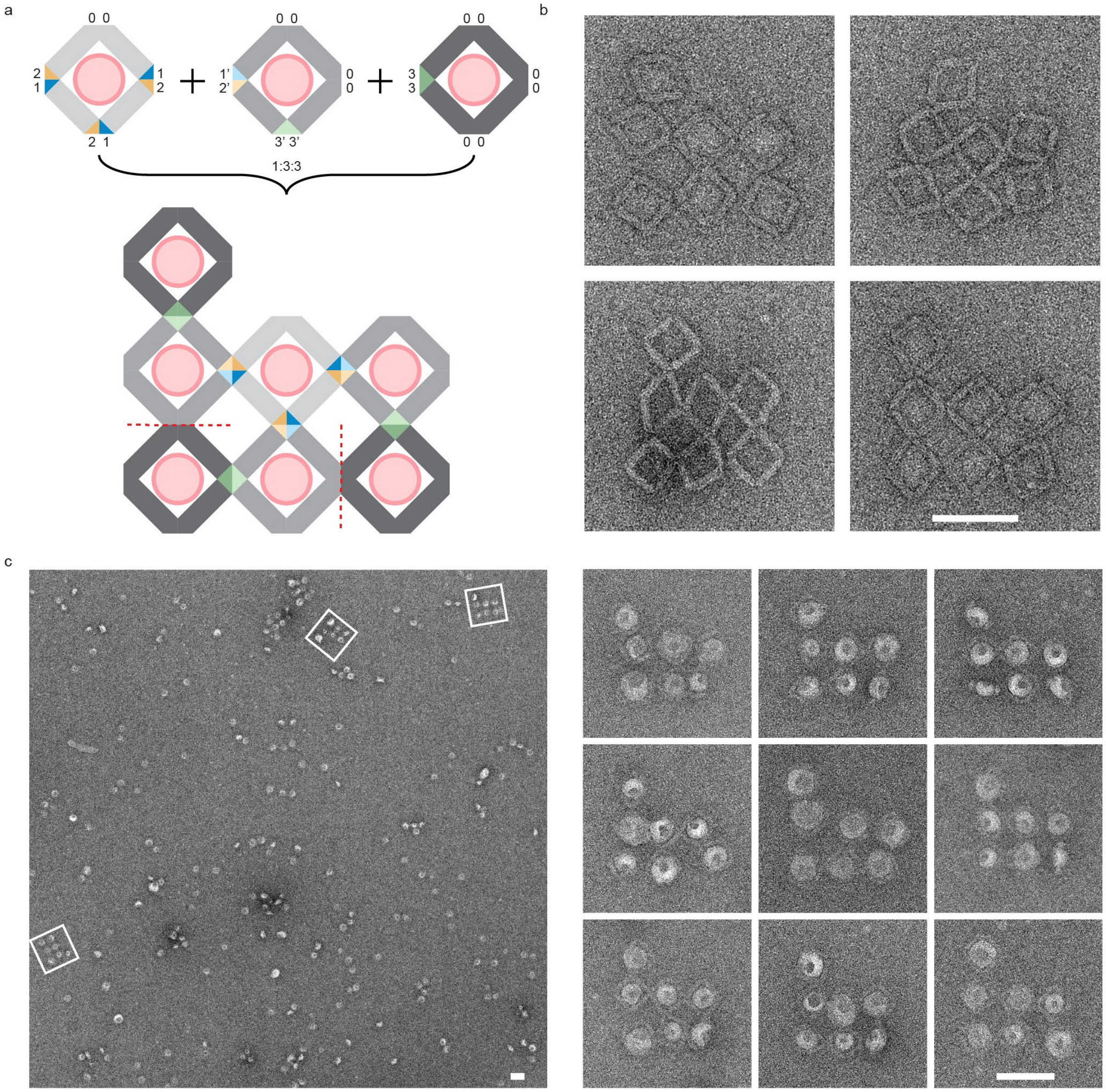

Figure S29. Assembly of STL 7-mer version 2 (v2). (a) Schematic illustration of 7-mer v2 formation by three STL variants in a 1:3:3 ratio. One variant carries chiral SEs (1/2) on three vertices; the second variant features complementary SEs (2'/1') on one vertex and an additional set of SEs (3') on the neighboring vertex; the third variant has complementary SEs (3) on a single vertex. The precise chirality of SEs ensures correct binding orientation. (b) Cropped TEM images of SDO 7-mer v2. (c) A wide-field TEM image (left) and a collection of cropped TEM images (right) showing assembled STL 7-mer v2. Scale bars: 100 nm.

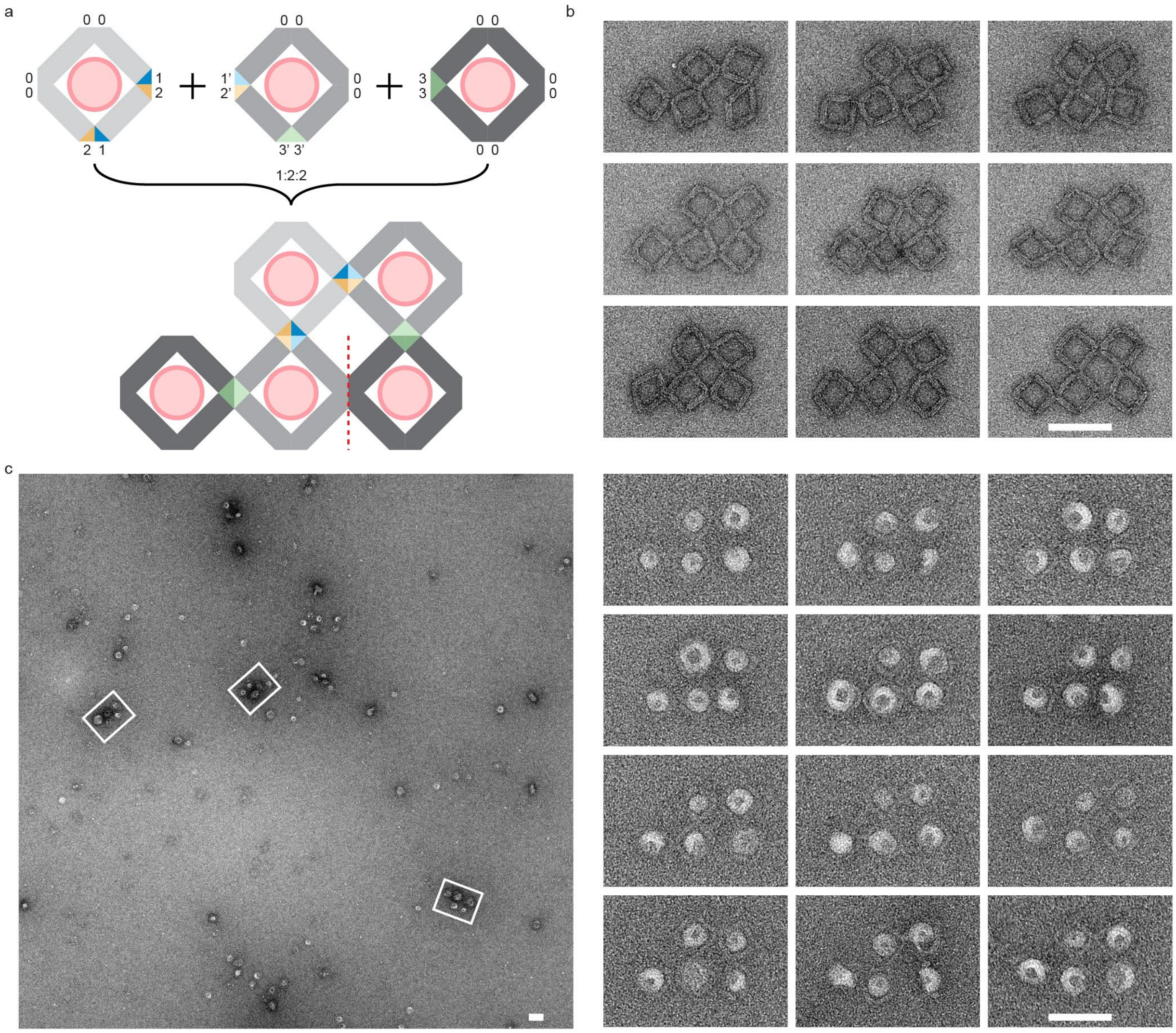

Figure S30. Assembly of STL 5-mer version 5 (v5). (a) Schematic illustration of 5-mer v5 formation by three STL variants in a 1:2:2 ratio. One variant carries chiral SEs (1/2) on two neighboring vertices with the same chirality; the second variant features complementary SEs (2'/1') on one vertex and an additional set of SEs (3') on the neighboring vertex; the third variant has complementary SEs (3) on a single vertex. The precise chirality of SEs ensures correct binding orientation. (b) Cropped TEM images of SDO 5-mer v5. (c) A wide-field TEM image (left) and a collection of cropped TEM images (right) showing assembled STL 5-mer v5. Scale bars: 100 nm.

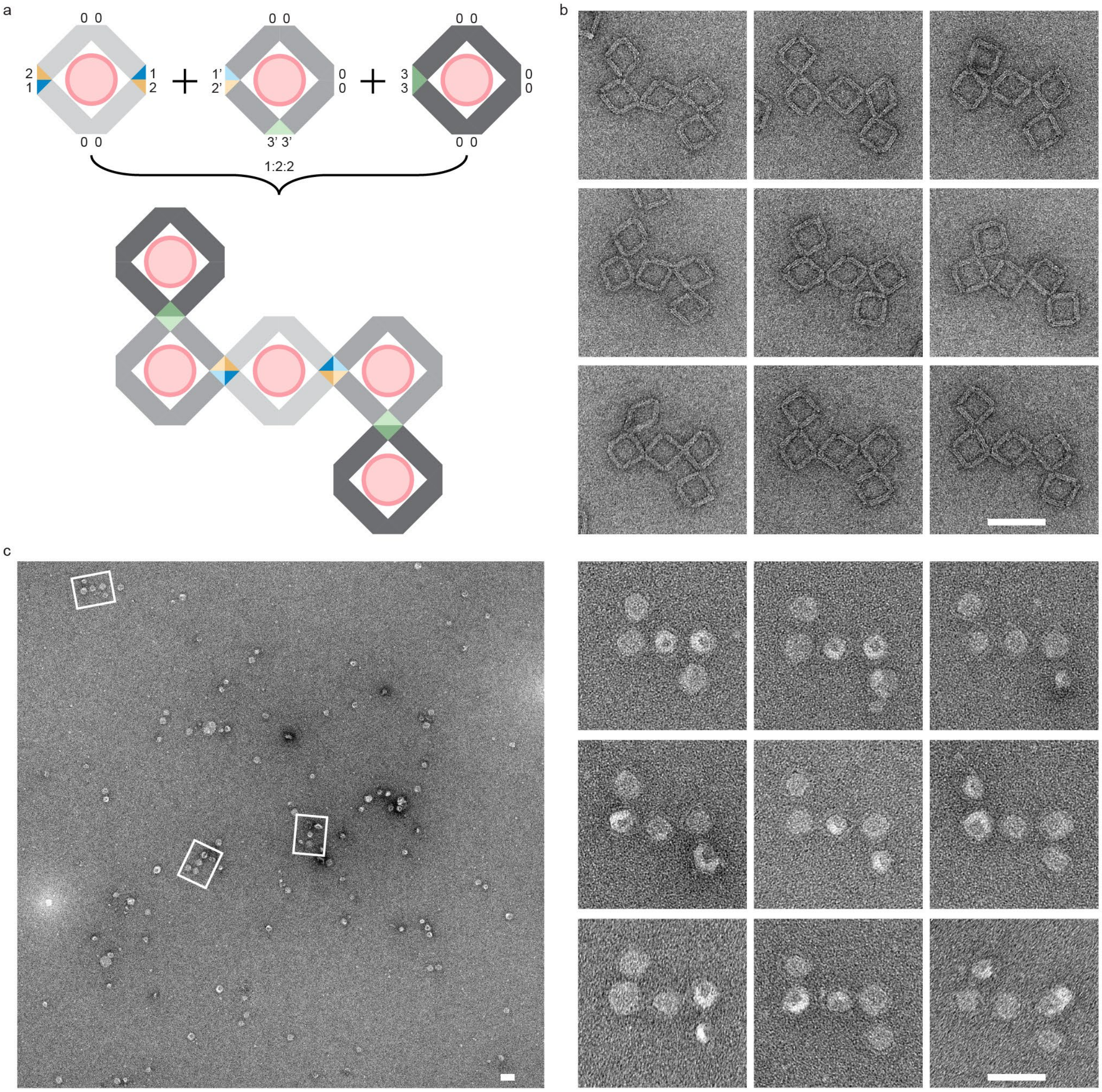

Figure S31. Assembly of STL 5-mer version 6 (v6). (a) Schematic illustration of 5-mer v6 formation by three STL variants in a 1:2:2 ratio. One variant carries chiral SEs (1/2) on two opposite vertices with the same chirality; the second variant features complementary SEs (2'/1') on one vertex and an additional set of SEs (3') on the neighboring vertex; the third variant has complementary SEs (3) on a single vertex. The precise chirality of SEs ensures correct binding orientation. (b) Cropped TEM images of SDO 5-mer v6. (c) A wide-field TEM image (left) and a collection of cropped TEM images (right) showing assembled STL 5-mer v6. Scale bars: 100 nm.

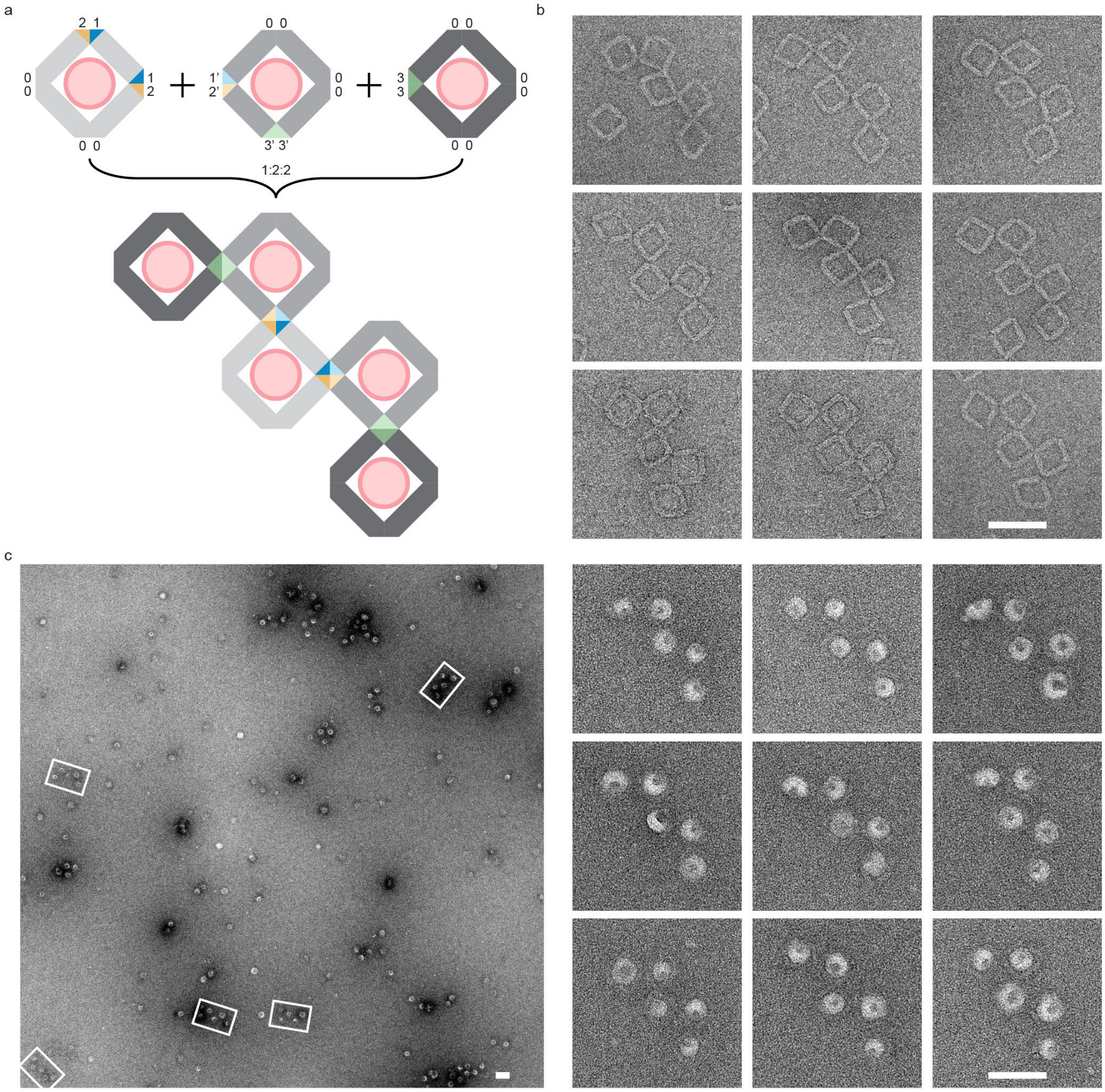

Figure S32. Assembly of STL 5-mer version 7 (v7). (a) Schematic illustration of 5-mer v7 formation by three STL variants in a 1:2:2 ratio. One variant carries chiral SEs (1/2) on two neighboring vertices with opposite chirality; the second variant features complementary SEs (2'/1') on one vertex and an additional set of SEs (3') on the neighboring vertex; the third variant has complementary SEs (3) on a single vertex. The precise chirality of SEs ensures correct binding orientation. (b) Cropped TEM images of SDO 5-mer v7. (c) A wide-field TEM image (left) and a collection of cropped TEM images (right) showing assembled STL 5-mer v7. Scale bars: 100 nm.

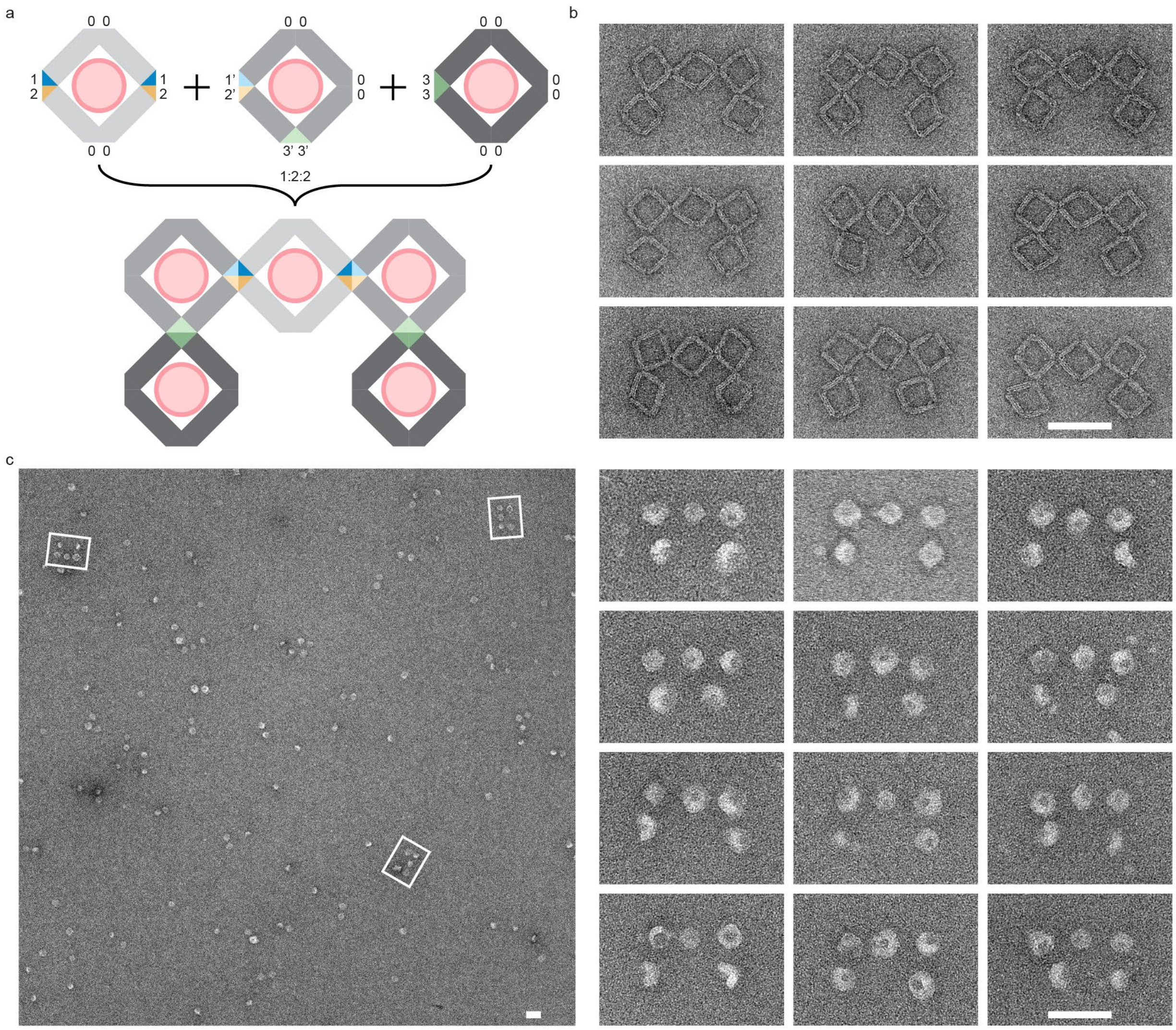

Figure S33. Assembly of STL 5-mer version 8 (v8). (a) Schematic illustration of 5-mer v8 formation by three STL variants in a 1:2:2 ratio. One variant carries chiral SEs (1/2) on two opposite vertices with opposite chirality; the second variant features complementary SEs (2'/1') on one vertex and an additional set of SEs (3') on the neighboring vertex; the third variant has complementary SEs (3) on a single vertex. The precise chirality of SEs ensures correct binding orientation. (b) Cropped TEM images of SDO 5-mer v8. (c) A wide-field TEM image (left) and a collection of cropped TEM images (right) showing assembled STL 5-mer v8. Scale bars: 100 nm.

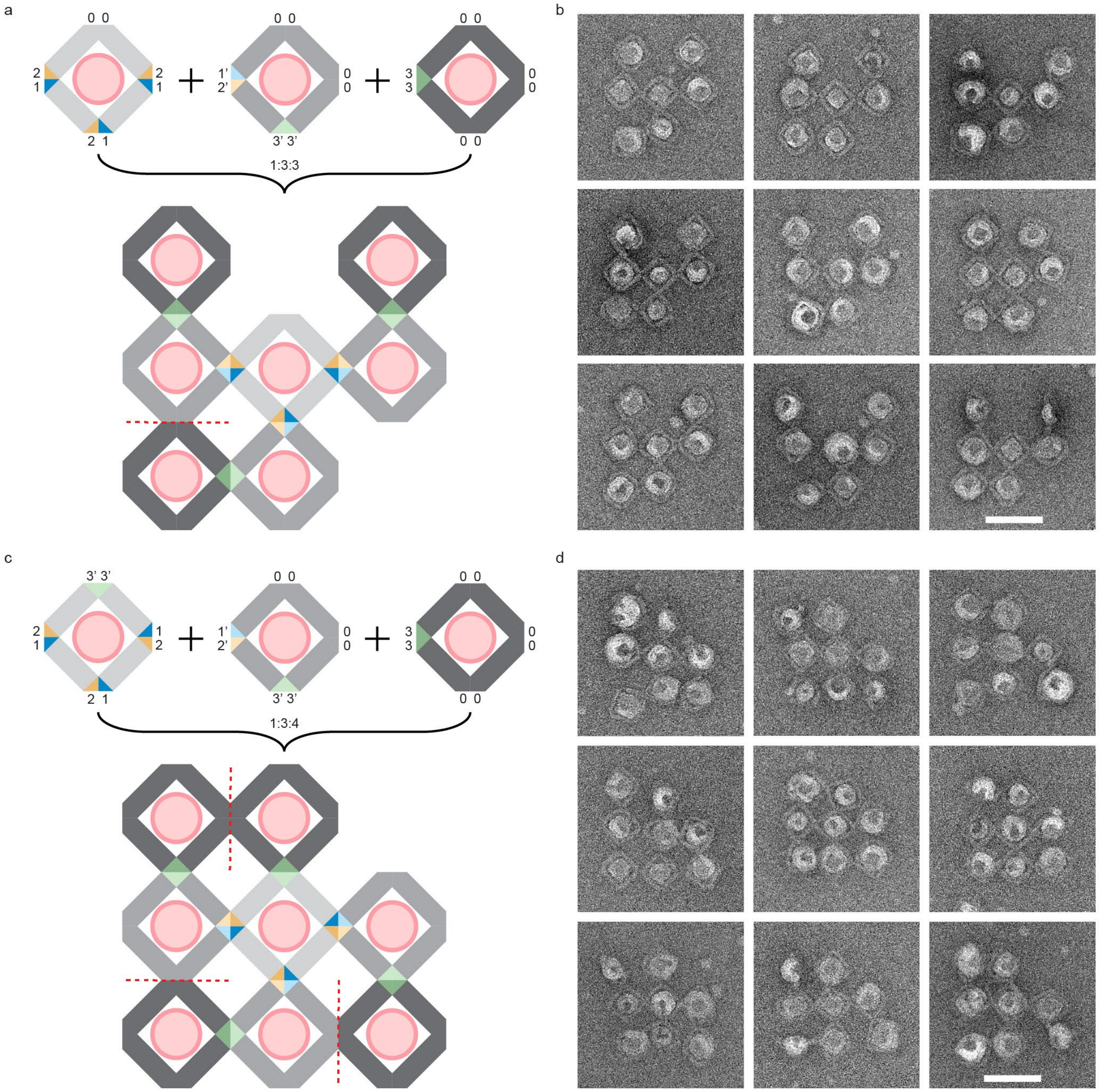

Figure S34. Assembly of STL 7-mer and 8-mer version 3 (v3). (a-b) Schematic illustration and cropped TEM images of 7-mer v3 formation by three STL variants in a 1:3:3 ratio. (c-d) Schematic illustration and cropped TEM images of 8-mer v3 formation by three STL variants in a 1:3:4 ratio. For 7-mer v3, variant 1 (LM1) carries chiral SEs (1/2) on three vertices with varied chirality, while for 8-mer v3, LM1 carries chiral SEs (1/2) on three vertices with the same chirality and an additional set of SEs (3') on the last vertex. For both 7-mer and 8-mer v3, variant 2 features complementary SEs (2'/1') on one vertex and an additional set of SEs (3') on the neighboring vertex, while variant 3 has complementary SEs (3) on a single vertex. The precise chirality of SEs ensures correct binding orientation. Scale bars: 100 nm.

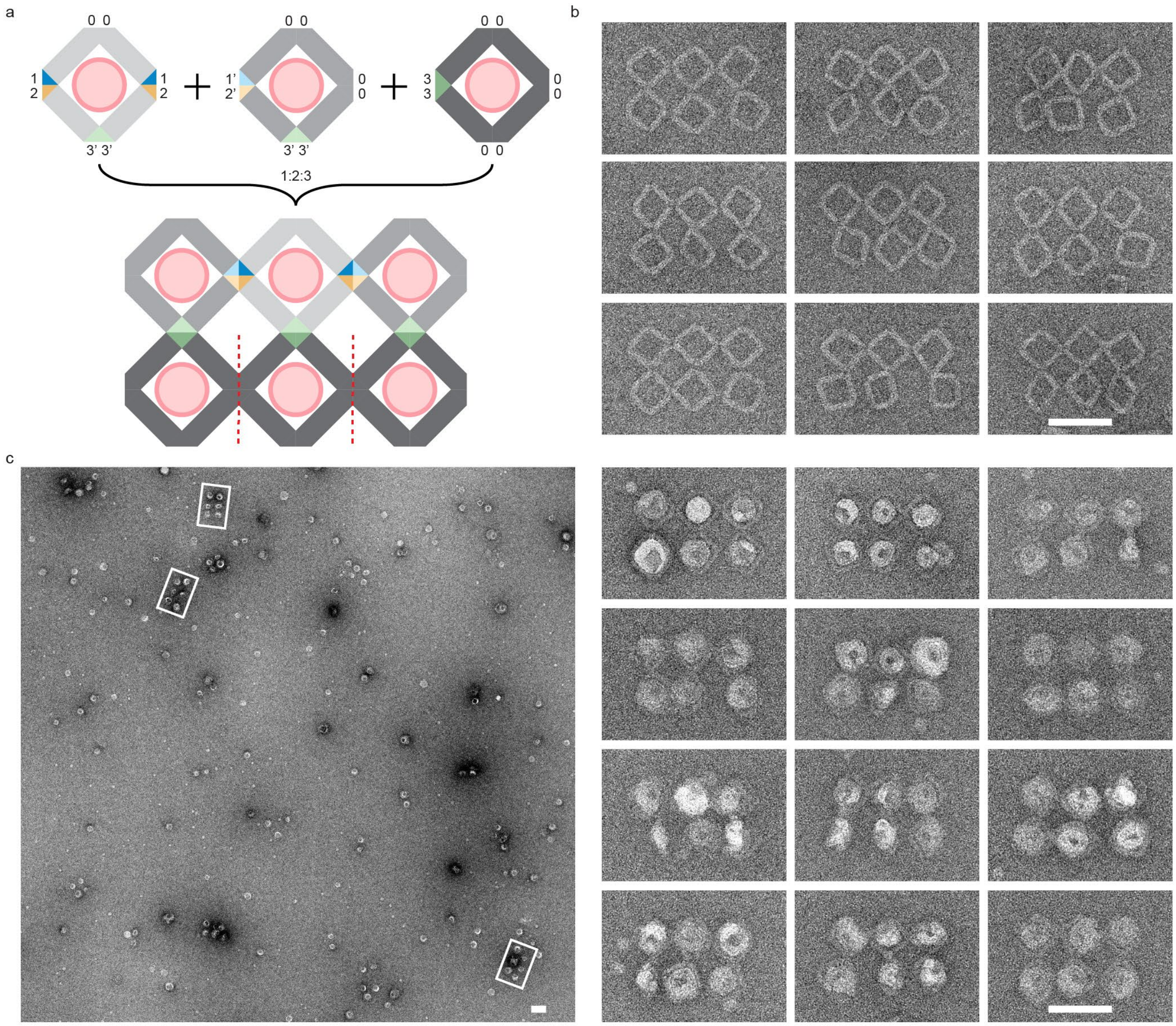

Figure S35. Assembly of STL 6-mer version 3 (v3). (a) Schematic illustration of 6-mer v3 formation by three STL variants in a 1:2:3 ratio. One variant carries chiral SEs (1/2) on two opposite vertices with opposite chirality and an additional set of SEs (3') on another vertex (adjacent to sticky end 2); the second variant features complementary SEs (2'/1') on one vertex and an additional set of SEs (3') on the neighboring vertex; the third variant has complementary SEs (3) on a single vertex. The precise chirality of SEs ensures correct binding orientation. (b) Cropped TEM images of SDO 6-mer v3. (c) A wide-field TEM image (left) and a collection of cropped TEM images (right) showing assembled STL 6-mer v3. Scale bars: 100 nm.

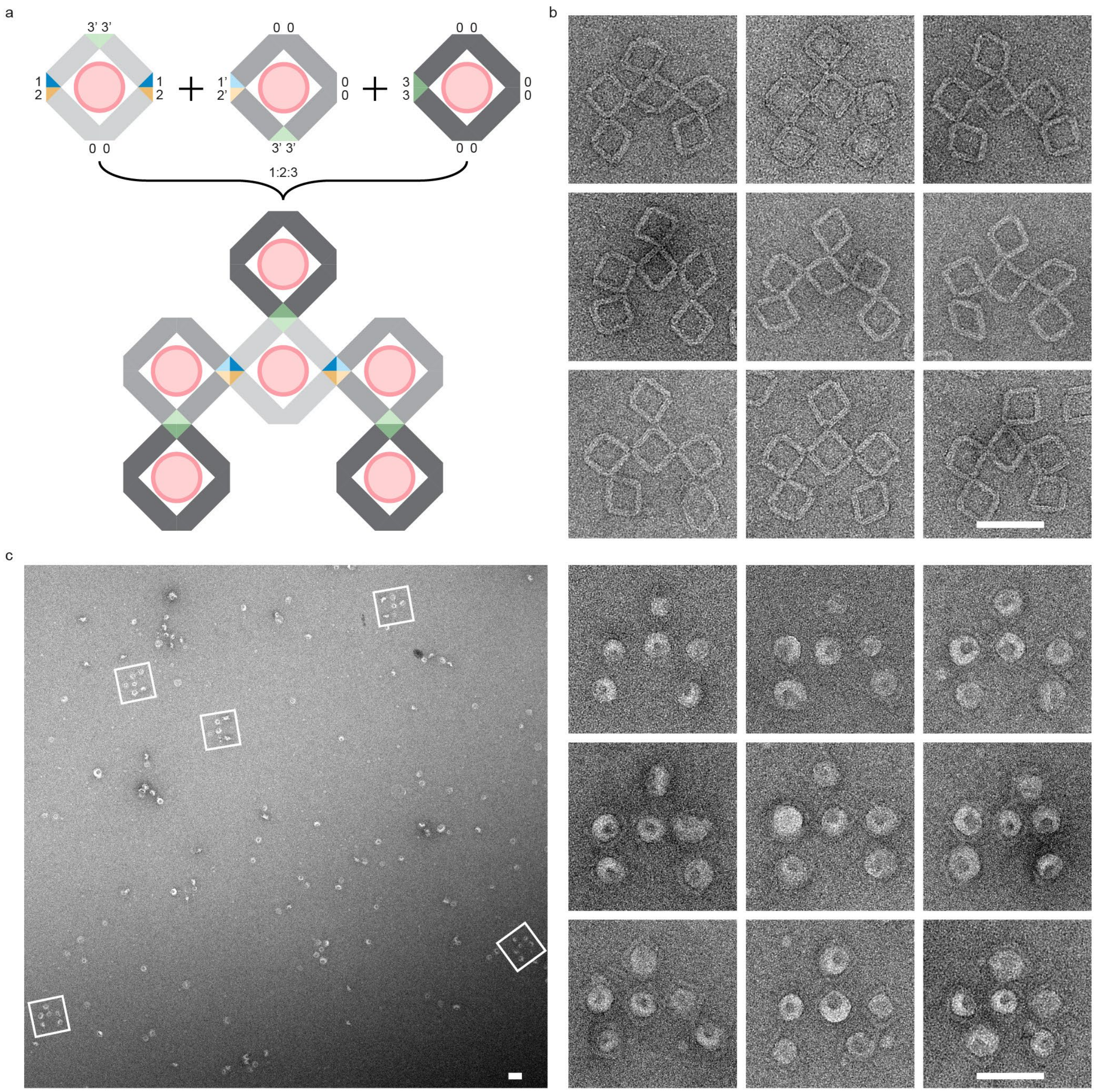

Figure S36. Assembly of STL 6-mer version 4 (v4). (a) Schematic illustration of 6-mer v4 formation by three STL variants in a 1:2:3 ratio. One variant carries chiral SEs (1/2) on two opposite vertices with opposite chirality and an additional set of SEs (3') on another vertex (adjacent to sticky end 1); the second variant features complementary SEs (2'/1') on one vertex and an additional set of SEs (3') on the neighboring vertex; the third variant has complementary SEs (3) on a single vertex. The precise chirality of SEs ensures correct binding orientation. (b) Cropped TEM images of SDO 6-mer v4. (c) A wide-field TEM image (left) and a collection of cropped TEM images (right) showing assembled STL 6-mer v4. Scale bars: 100 nm.

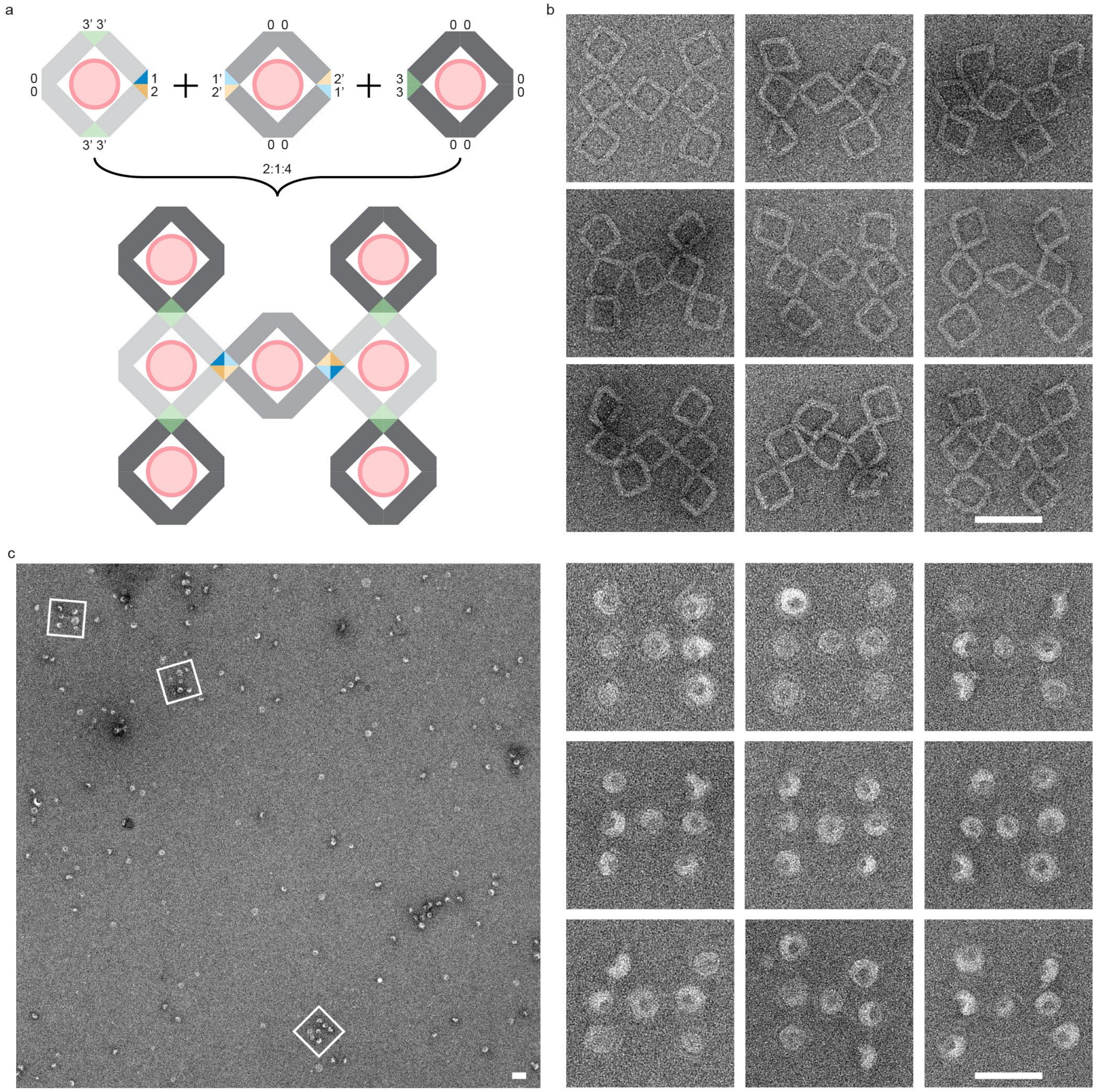

Figure S37. Assembly of STL 7-mer version 4 (v4). (a) Schematic illustration of 7-mer v4 formation by three STL variants in a 2:1:4 ratio. One variant carries chiral SEs (1/2) on one vertex and an additional set of SEs (3') on two neighboring vertices; the second variant features complementary SEs (2'/1') on two opposite vertices; the third variant has complementary SEs (3) on a single vertex. (b) Cropped TEM images of SDO 7-mer v4. (c) A wide-field TEM image (left) and a collection of cropped TEM images (right) showing assembled STL 7-mer v4. Scale bars: 100 nm.

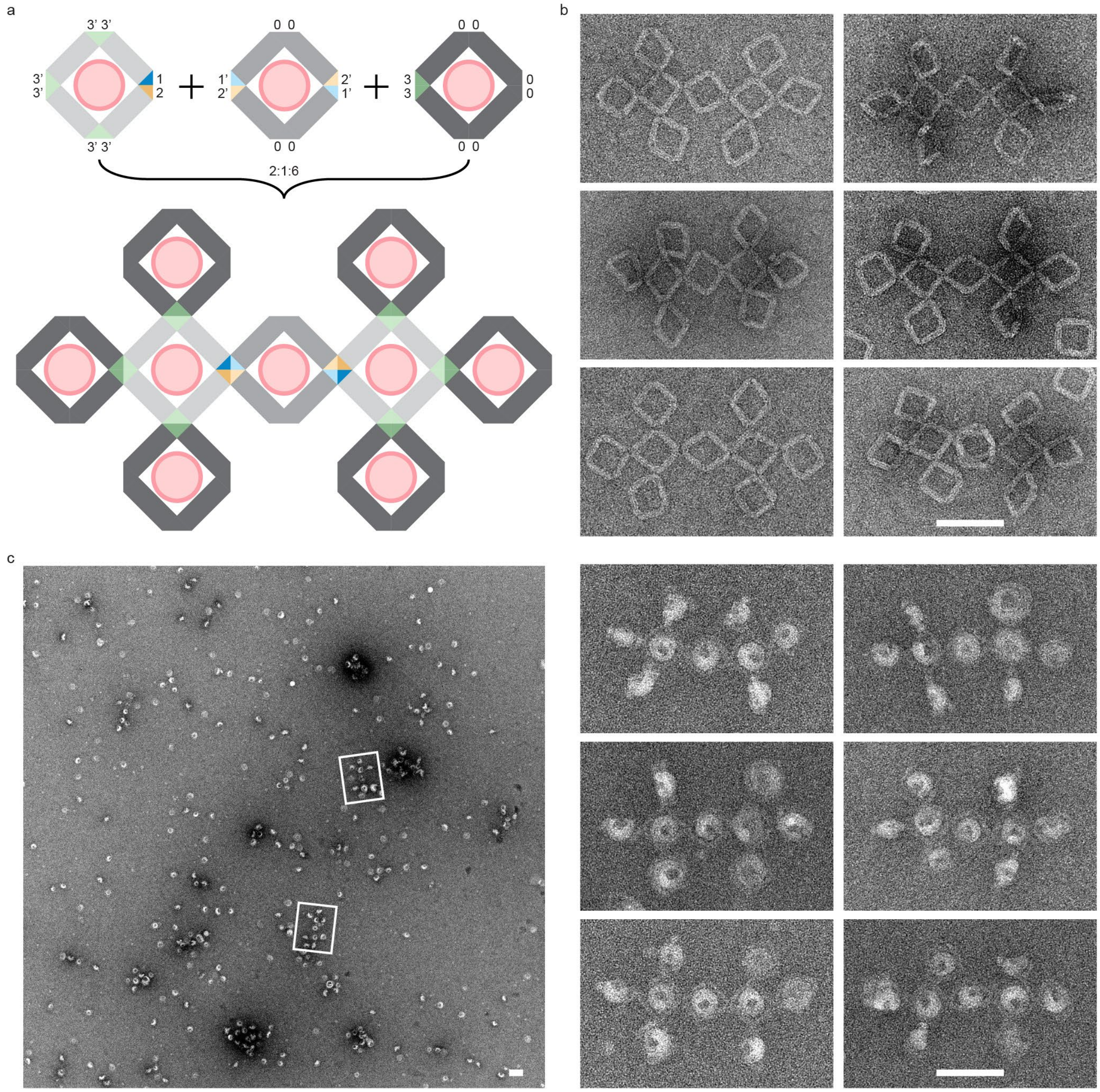

Figure S38. Assembly of STL 9-mer version 3 (v3). (a) Schematic illustration of 9-mer v3 formation by three STL variants in a 2:1:6 ratio. One variant carries chiral SEs (1/2) on one vertex and an additional set of SEs (3') on the other three vertices; the second variant features complementary SEs (2'/1') on two opposite vertices; the third variant has complementary SEs (3) on a single vertex. (b) Cropped TEM images of SDO 9-mer v3. (c) A wide-field TEM image (left) and a collection of cropped TEM images (right) showing assembled STL 9-mer v3. Scale bars: 100 nm.

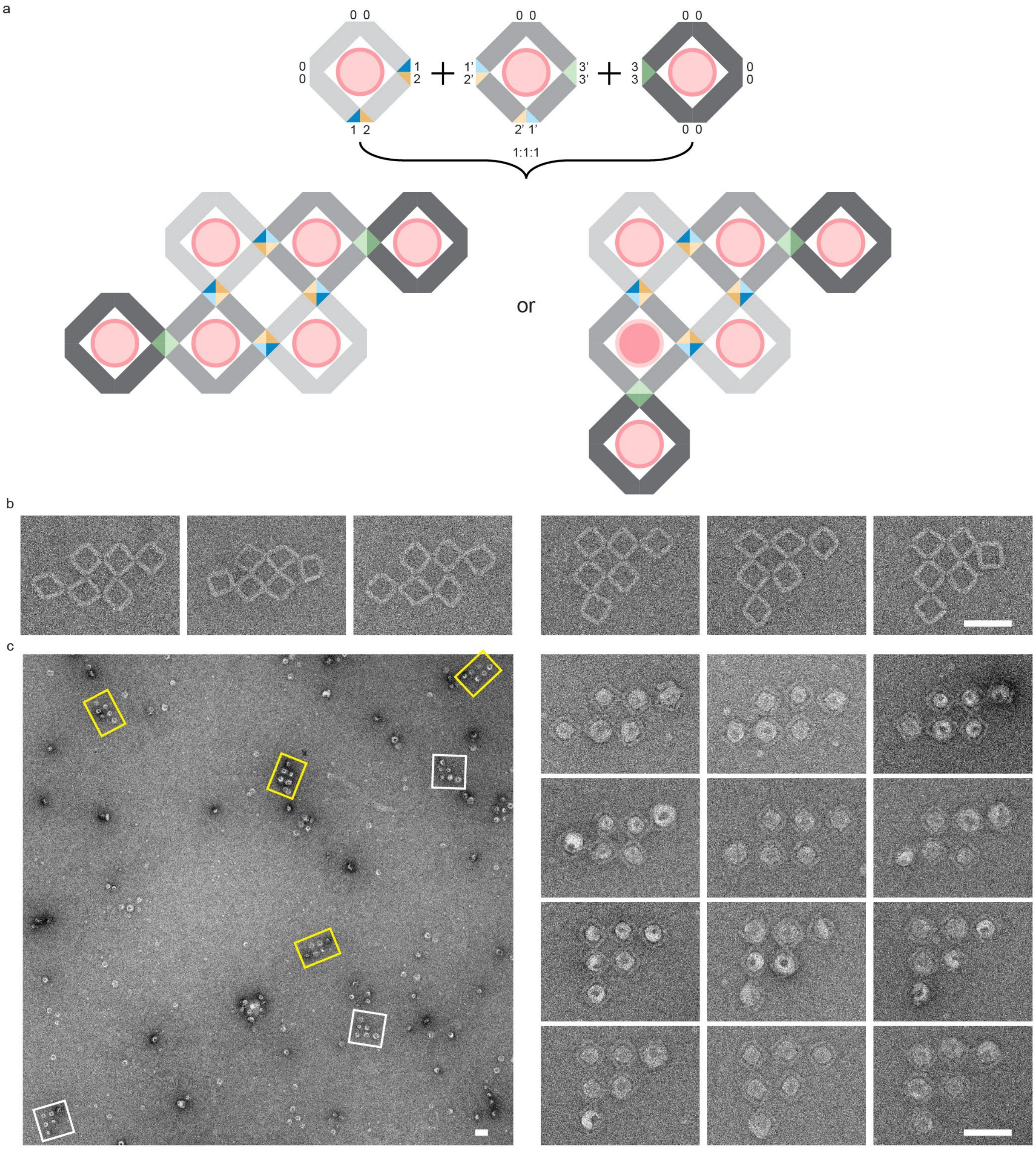

Figure S39. Assembly of STL 6-mer mixture. (a) Schematic illustration of 6-mer mixture formation by three STL variants in a 1:1:1 ratio. One variant (LM1) carries chiral SEs (1/2) on two neighboring vertices with opposite chirality; the second variant (LM2) features complementary SEs (2'/1') on two neighboring vertices with opposite chirality and an additional set of SEs (3') on another vertex; the third variant (LM3) has complementary SEs (3) on a single vertex. Upon binding, LM1 and LM2 form a square-shaped tetramer, either all facing up or with one of the LM2 (highlighted in pink) facing down. This variation results in two distinct configurations after LM3 binds to LM2. (b) Cropped TEM images of SDO 6-mer mixture. (c) A wide-field TEM image (left)

and a collection of cropped TEM images (right) showing assembled STL 6-mer mixture. Scale bars: 100 nm.

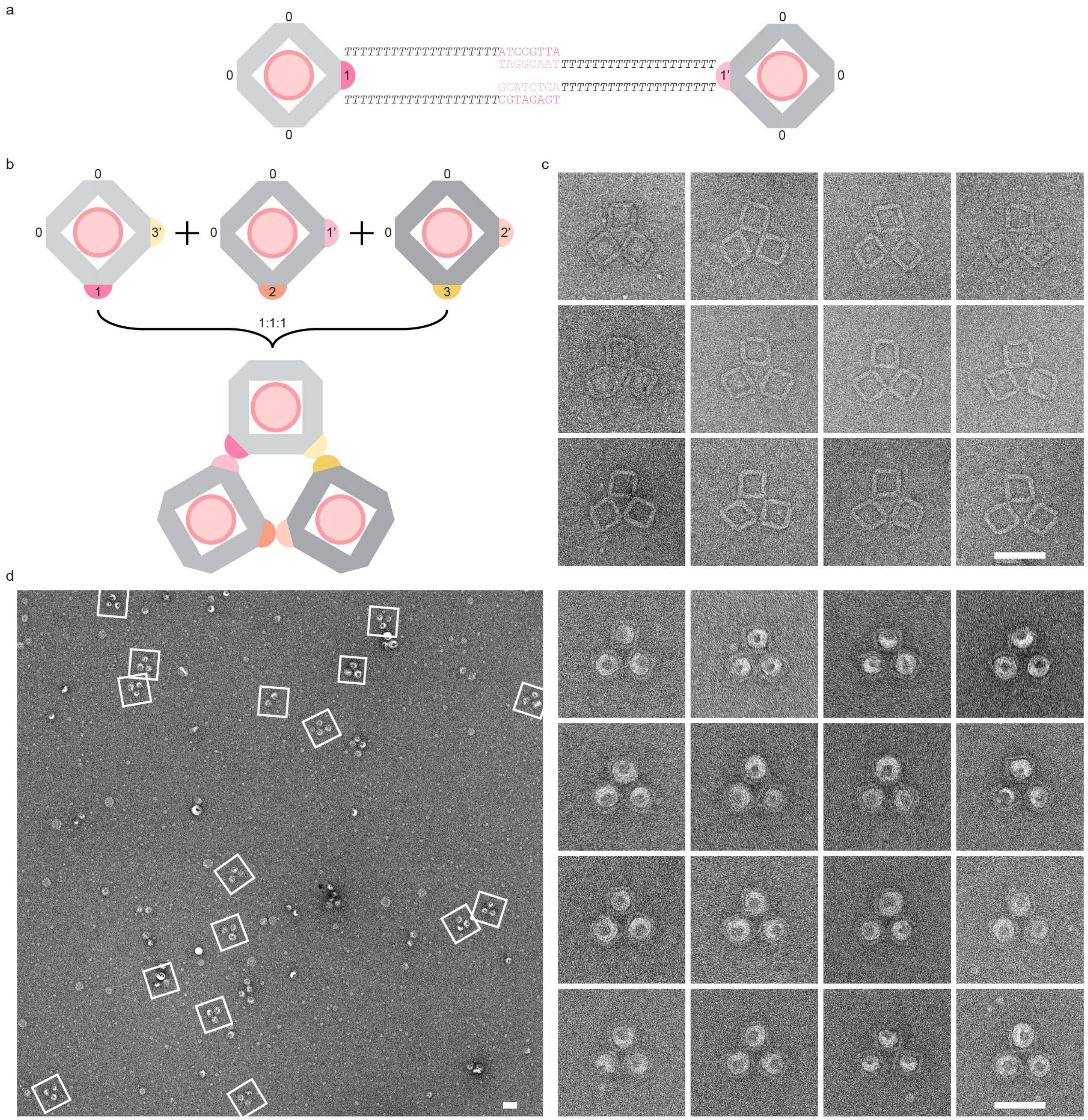

Figure S40. Assembly of the STL trimer ring. (a) A T20 spacer is incorporated between the origami body and the 8-nt SE, providing flexibility that allows angles other than 180 degrees between two interacting monomers. In the cartoon models, a single-color semicircle represents a chiral T20 SE. (b) Schematic illustration of trimer ring formation by three equimolar STL variants featuring complementary chiral T20 SEs on a pair of neighboring vertices. The precise chirality of SEs ensures correct binding orientation. (c) Cropped TEM images of SDO trimer rings. (d) A wide-field TEM image (left) and a collection of cropped TEM images (right) showing assembled STL trimer rings. Scale bars: 100 nm.

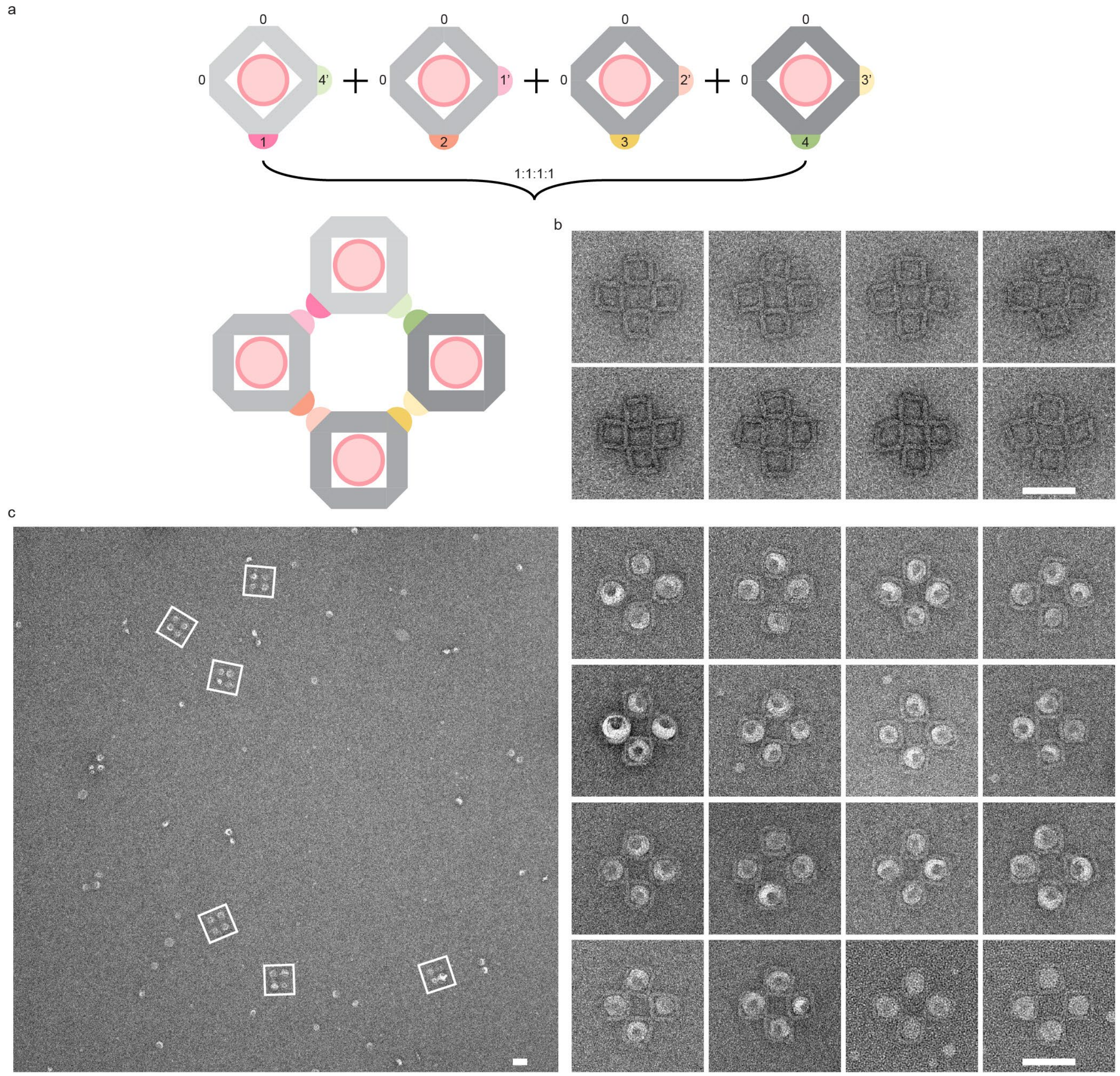

Figure S41. Assembly of the STL tetramer ring. (a) Schematic illustration of tetramer ring formation by four equimolar STL variants featuring complementary chiral T20 SEs on a pair of neighboring vertices. The precise chirality of SEs ensures correct binding orientation. (b) Cropped TEM images of SDO tetramer rings. (c) A wide-field TEM image (left) and a collection of cropped TEM images (right) showing assembled STL tetramer rings. Scale bars: 100 nm.

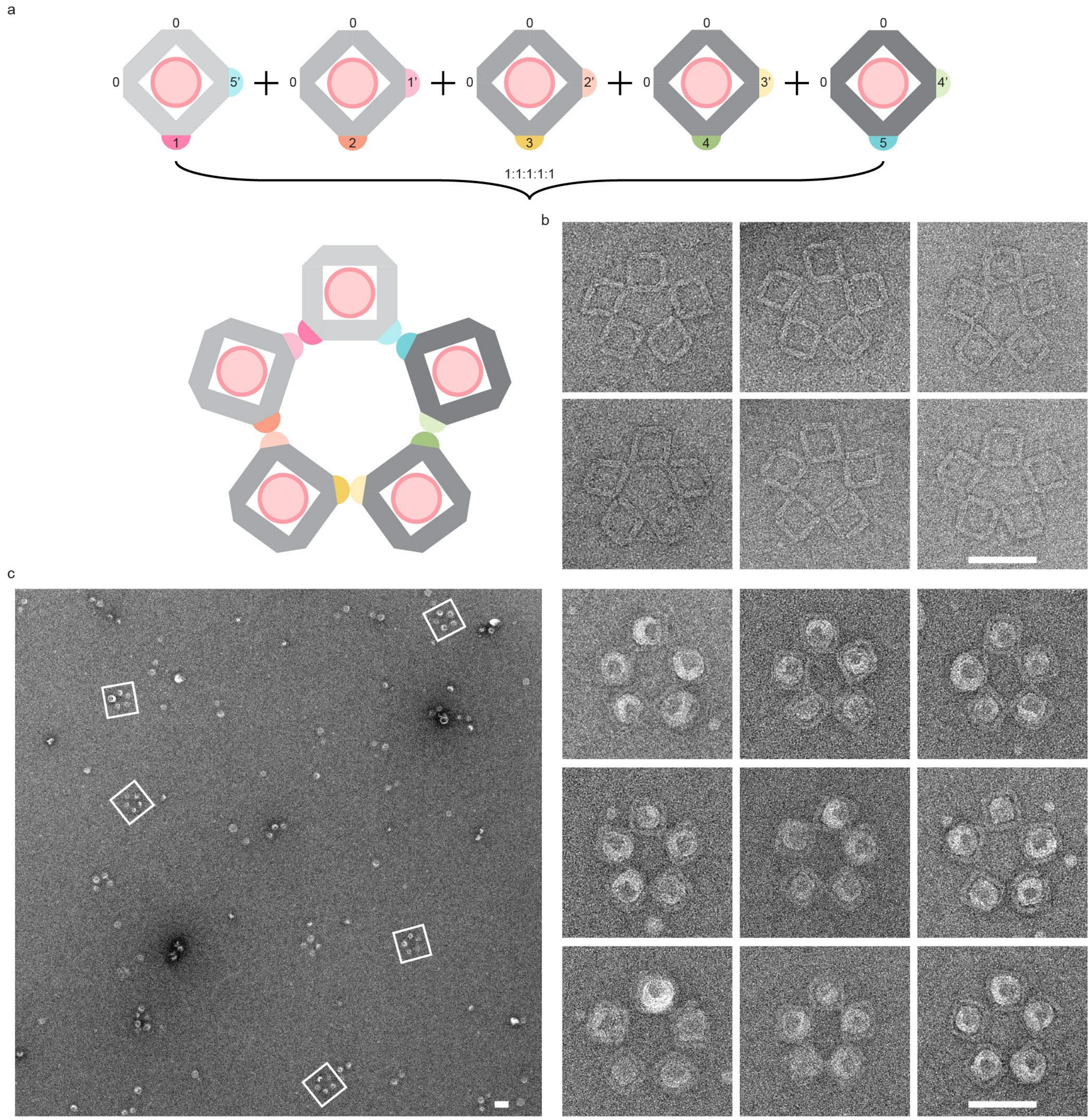

Figure S42. Assembly of the STL 5-mer ring. (a) Schematic illustration of 5-mer ring formation by five equimolar STL variants featuring complementary chiral T20 SEs on a pair of neighboring vertices. The precise chirality of SEs ensures correct binding orientation. (b) Cropped TEM images of SDO 5-mer rings. (c) A wide-field TEM image (left) and a collection of cropped TEM images (right) showing assembled STL 5-mer rings. Scale bars: 100 nm.

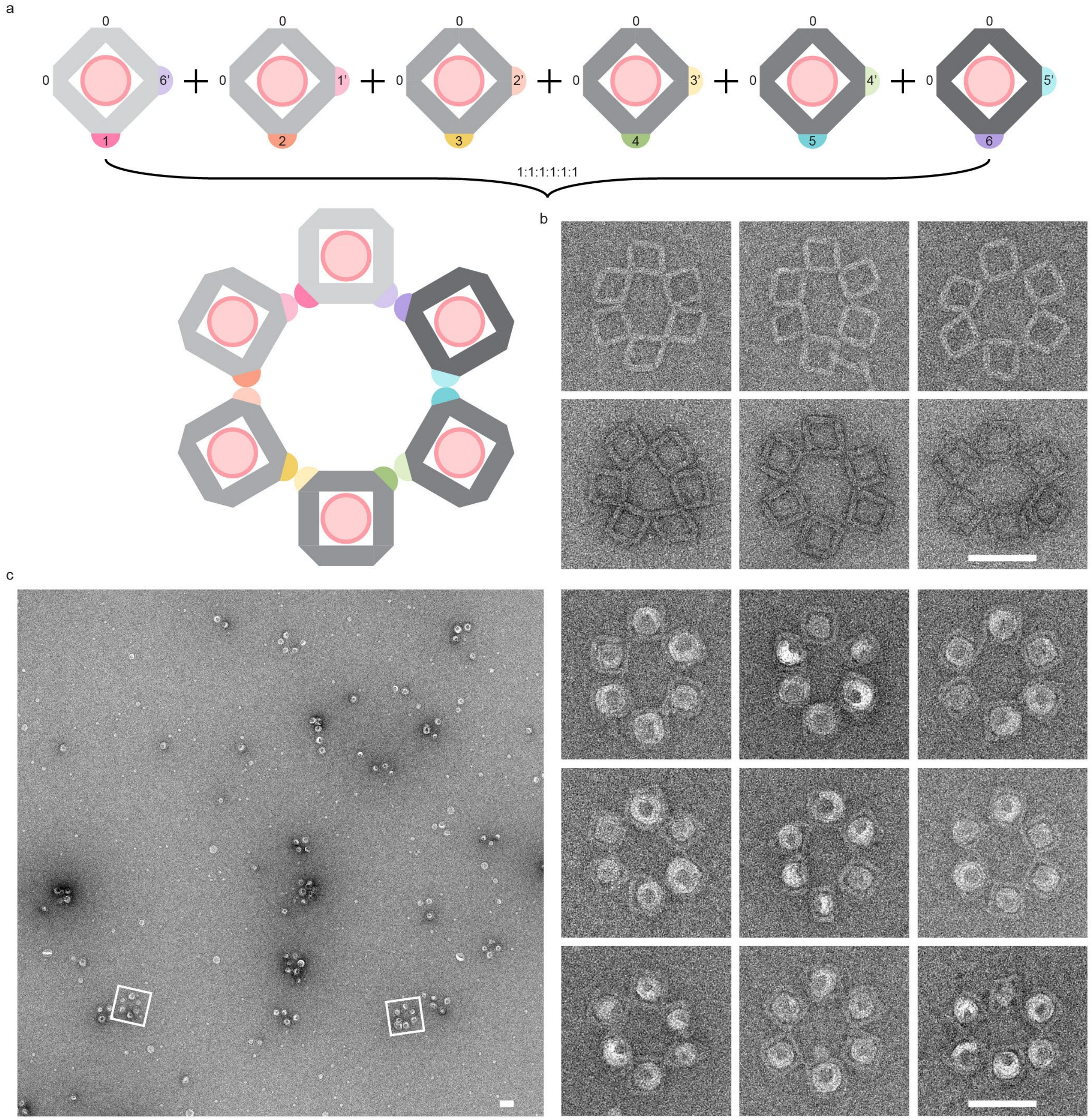

Figure S43. Assembly of the STL 6-mer ring. (a) Schematic illustration of 6-mer ring formation by six equimolar STL variants featuring complementary chiral T20 SEs on a pair of neighboring vertices. The precise chirality of SEs ensures correct binding orientation. (b) Cropped TEM images of SDO 6-mer rings. (c) A wide-field TEM image (left) and a collection of cropped TEM images (right) showing assembled STL 6-mer rings. Scale bars: 100 nm.

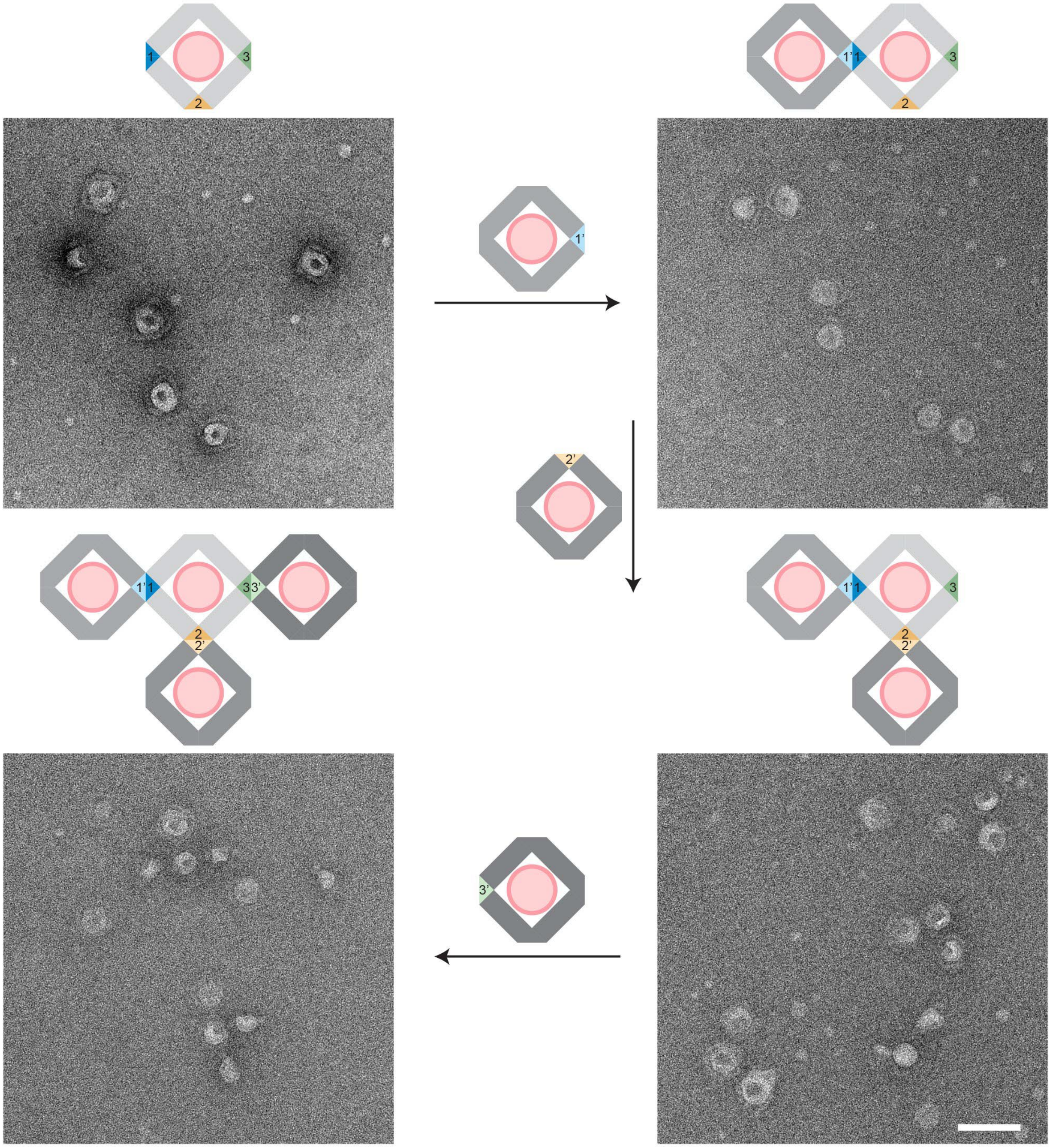

Figure S44. Stepwise assembly of STL hetero-tetramers. The central STL monomers contained three distinct SEs (1, 2, and 3) on separate vertices (top left). By sequentially adding three STL variants with complementary SEs (1', 2' or 3') on a single vertex, a dimer (top right), trimer (bottom right), and ultimately a tetramer (bottom left) was formed. In each step, the mixture was annealed from 40 °C to 22 °C over 3 hours. A cartoon model and a wide-field TEM image are shown for each construct. Scale bar: 100 nm.

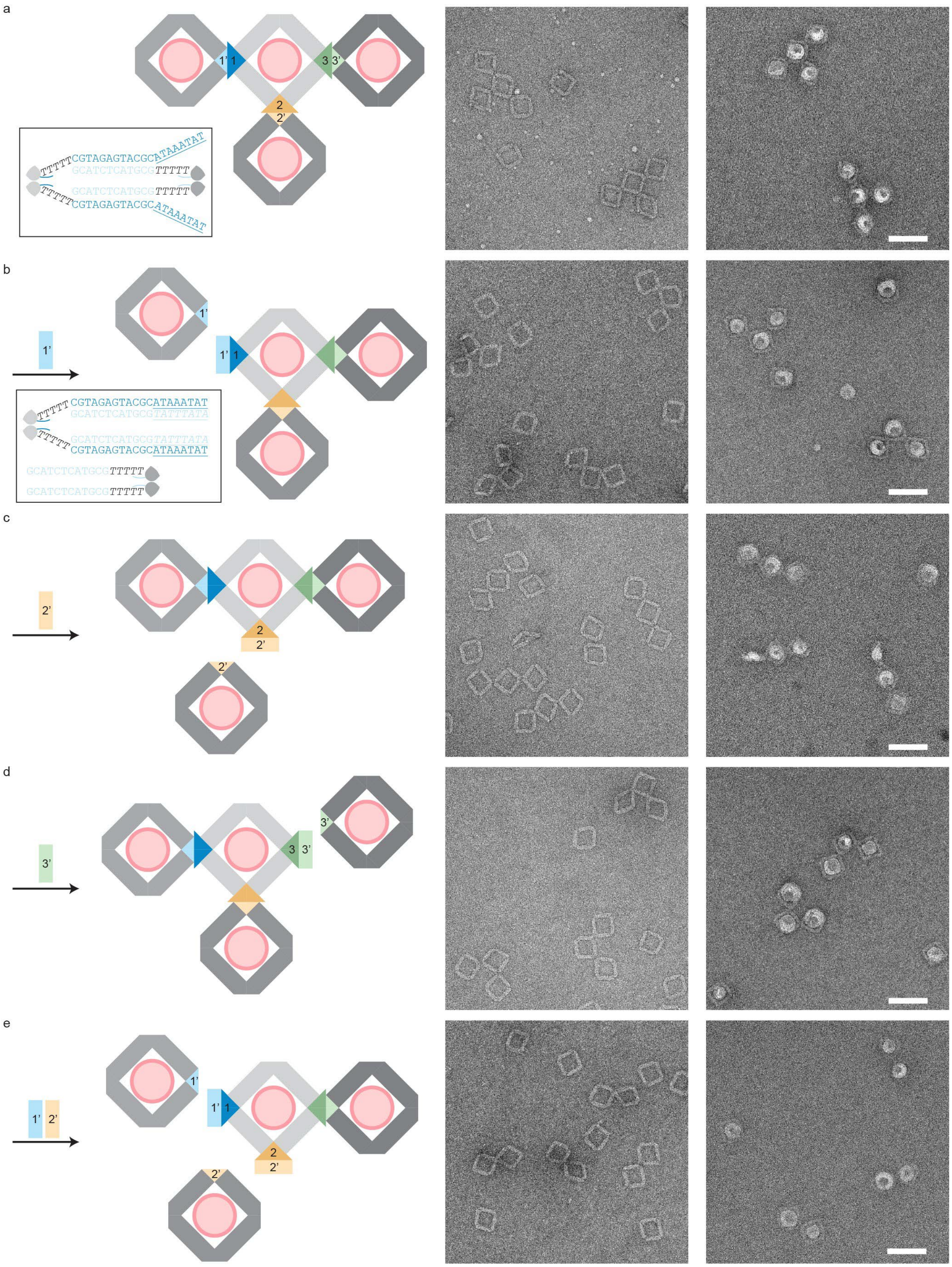

a
1' 1
3 3'
2
2'
TTTTTCGTAGAGTACGCATAAATAT
GCATCTCATGCGTTTTT
GCATCTCATGCGTTTTT
TTTTTCGTAGAGTACGCATAAATAT
b
1'
1'
1' 1
TTTTTCGTAGAGTACGCATAAATAT
GCATCTCATGCGTATTTATA
GCATCTCATGCGTATTTATA
TTTTTCGTAGAGTACGCATAAATAT
GCATCTCATGCGTTTTT
GCATCTCATGCGTTTTT
c
2'
2
2'
2'
d
3'
3'
3 3'
e
1' 2'
1'
1' 1
2
2'
2'

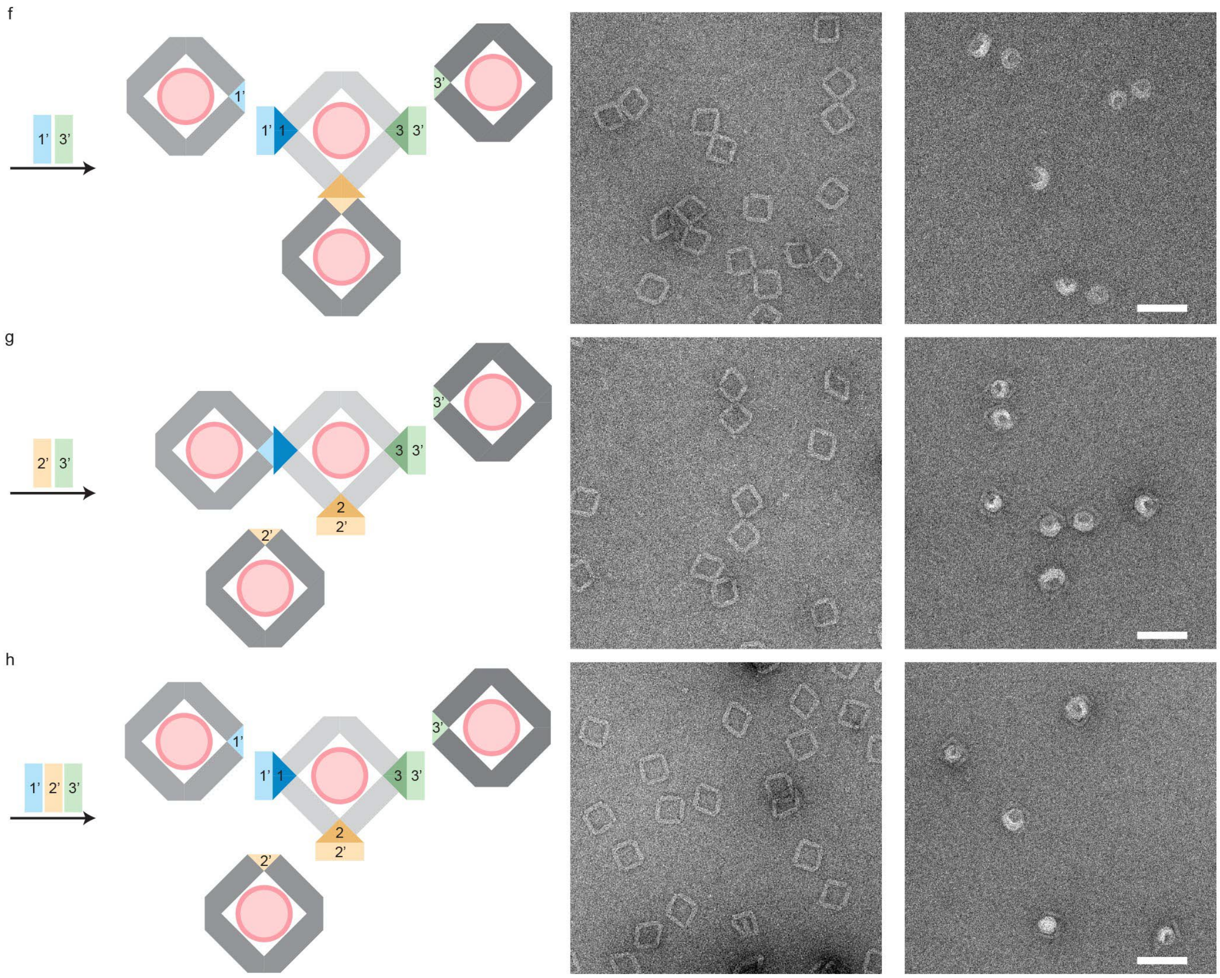

Figure S45. Targeted disassembly of a STL hetero-tetramer. Three peripheral STL monomers bind to the central monomers through distinct SE pairings (1-1', 2-2', and 3-3') on separate vertices. An 8-nt toehold was incorporated at the end of each SE on the central monomer (inset in (a)), allowing a fully complementary strand to displace the partially complementary SE on the peripheral monomers via TMSD (inset in (b)). As a result, the preformed hetero-tetramer (a) was selectively disassembled when specific combination of displacing strands were introduced and incubated at 37 °C for 2 hours. (b-h) A cartoon model and a wide-field TEM image are shown for the products of each reaction using SDO (middle) or STL (right). Scale bars: 100 nm.

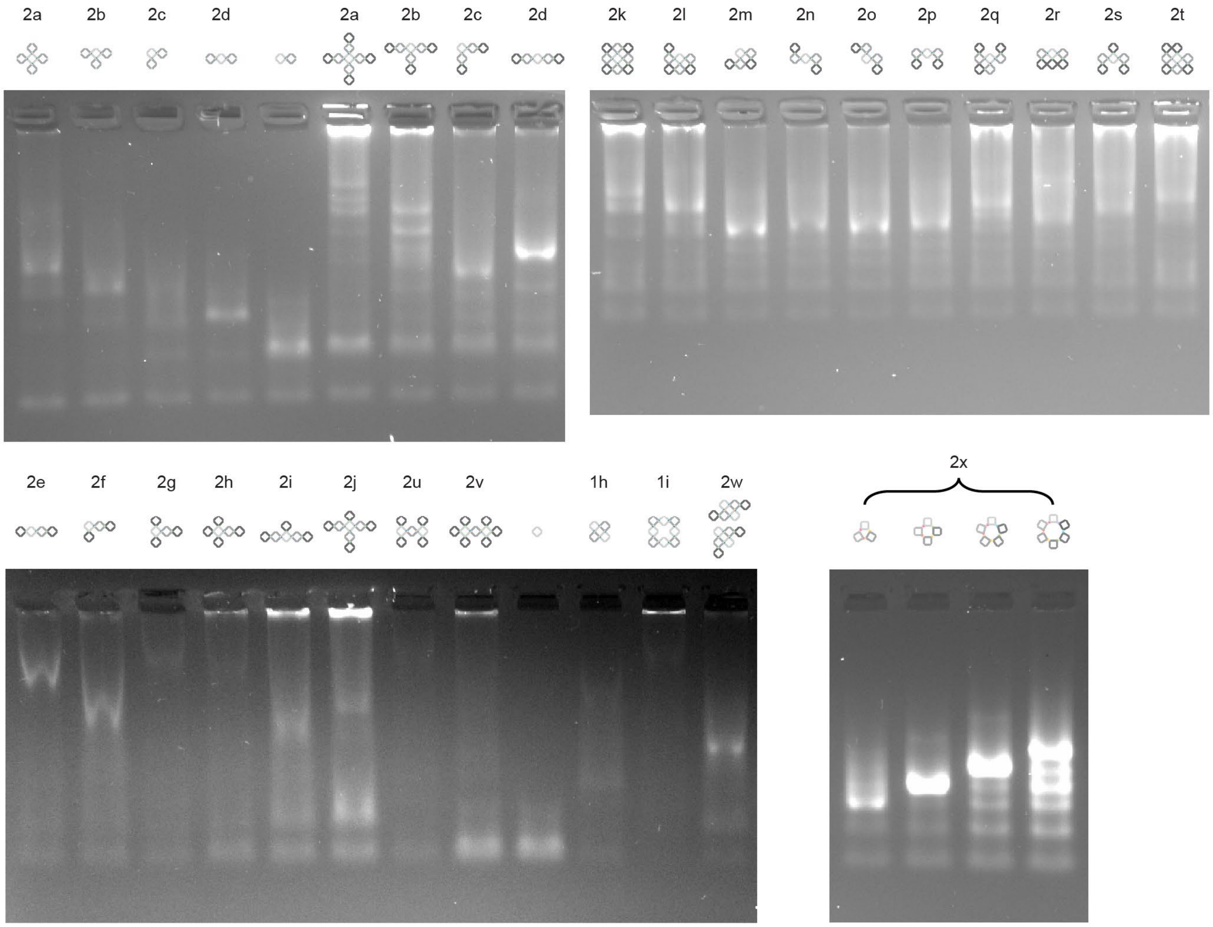

Figure S46. Agarose gel analysis of SDO finite lattices. Each lane corresponds to an SDO oligomer, with its schematic and associated panel labeled above. Assembly yields are quantified in Supplementary Table S2.

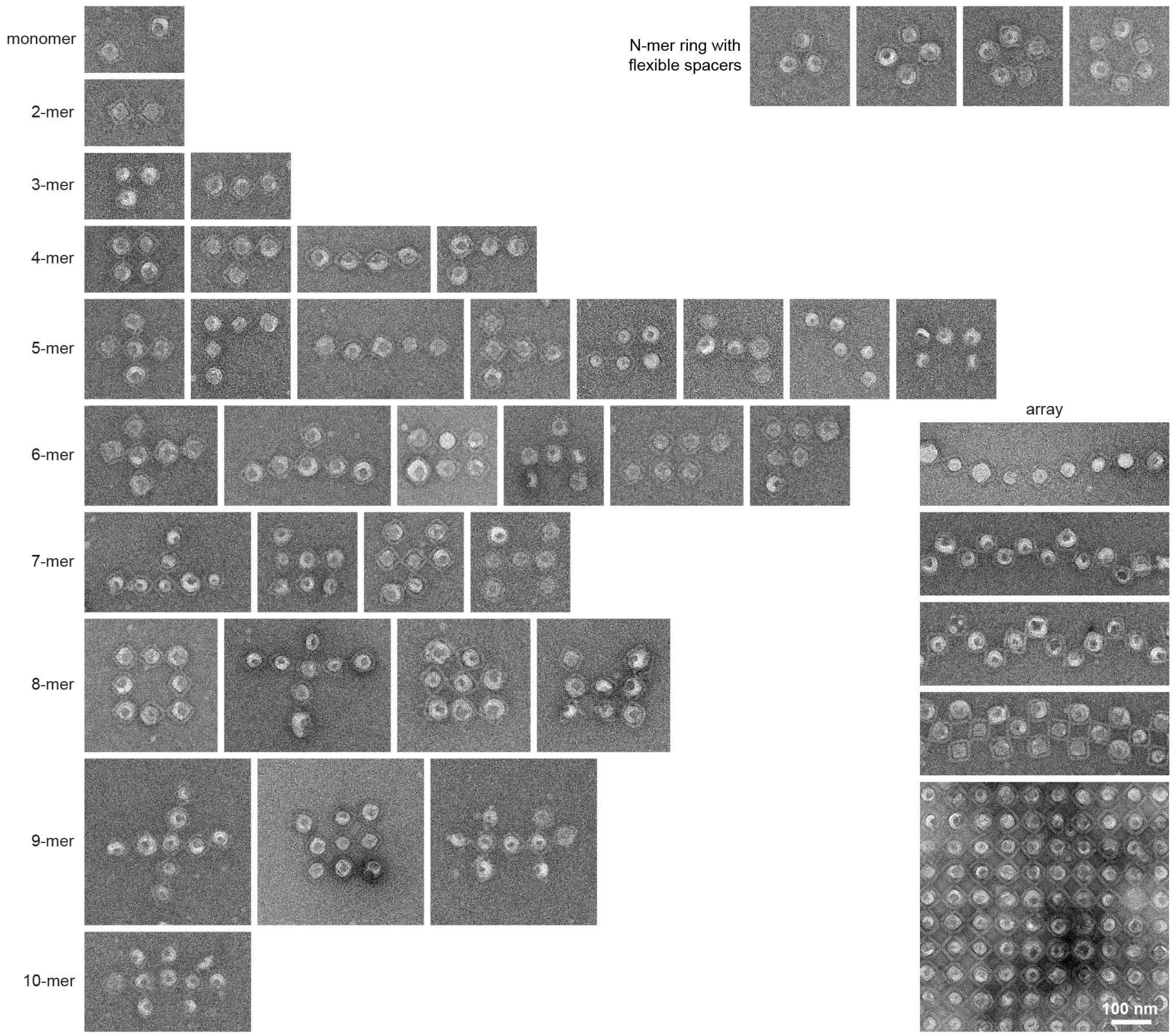

Figure S47. Summary of STL polymers created in this study. Each configuration (finite lattice, rings, or arrays) is represented by a cropped TEM image. The diversity of products highlights the programmability of STL higher-order assembly.

Supplementary Tables

Table S1. Degree of polymerization (DP) of STL arrays.

Construct	Figure (LM1:LM2)	DP (mean ± SD)	DP (median)	DP (Max)	N
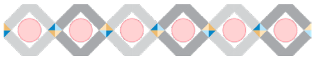	1d (1:1)	7.8±3.3	7	20	39
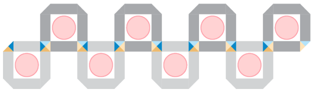	1f (1:1)	7.4±2.7	7	15	60
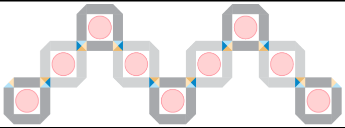	1g (1:1)	8.4±4.0	7	25	48
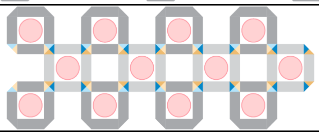	1e (1:2)	14.5±5.2	12	30	27

Quantification was based on wide-field TEM images, counting only monomers within correctly formed polymers containing four or more units.

Table S2. Summary of assembly yields of finite lattices.

Construct	Figure (LM1:LM2:LM3)	SDO polymerization yield quantified by gel	STL polymerization yield quantified by TEM
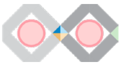	(1:1)	60%	46%
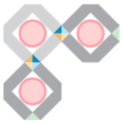	2c<br>(1:2)	23%	22%
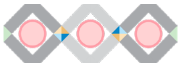	2d<br>(1:2)	47%	42%
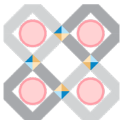	1h<br>(2:2)	36%	38%
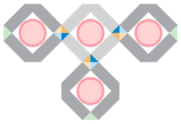	2b<br>(1:3)	41%	26%
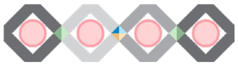	2e<br>(1:1:2)	29%	8%
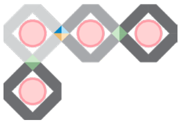	2f<br>(1:1:2)	33%	8%
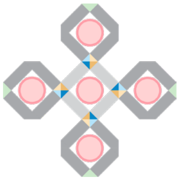	2a<br>(1:4)	25%	17%
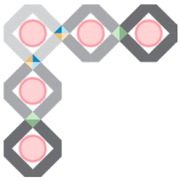	2c<br>(1:2:2)	27%	5%
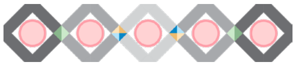	2d<br>(1:2:2)	36%	7%
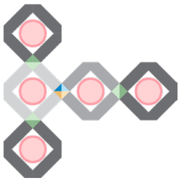	2g<br>(1:1:3)	10%	8%
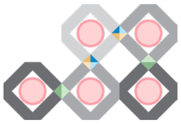	2m<br>(1:2:2)	40%	4%

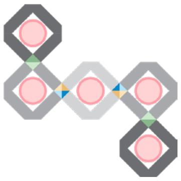	2n<br>(1:2:2)	34%	5%
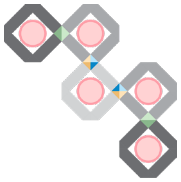	2o<br>(1:2:2)	38%	4%
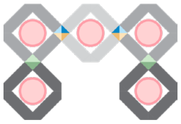	2p<br>(1:2:2)	37%	10%
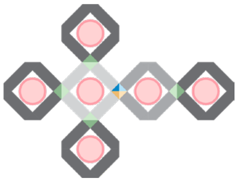	2h<br>(1:1:4)	6%	7%
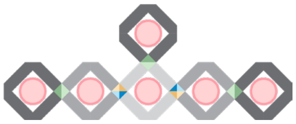	2i<br>(1:2:3)	20%	8%
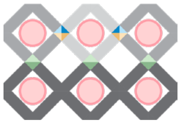	2r<br>(1:2:3)	15%	8%
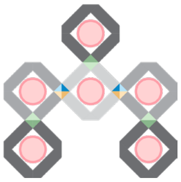	2s<br>(1:2:3)	17%	9%
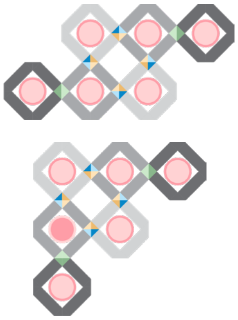	2w<br>(2:2:2) (mixture)	33%	7%+10%

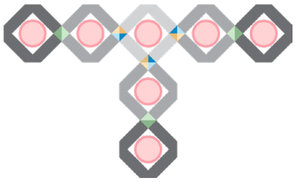	2b (1:3:3)	10%	6%
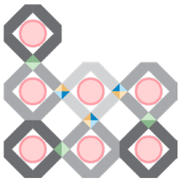	2l (1:3:3)	26%	8%
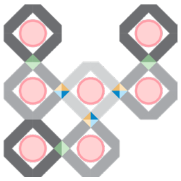	2q (1:3:3)	20%	11%
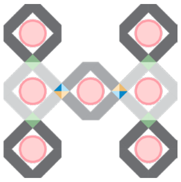	2u (1:2:4)	8%	5%
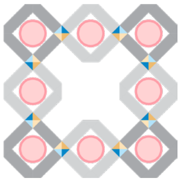	1i (4:4)	13%	23%
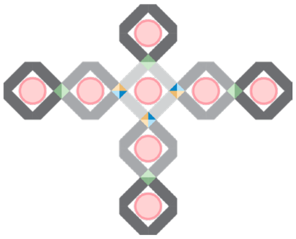	2j (1:3:4)	10%	10%
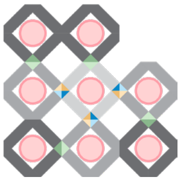	2t (1:3:4)	14%	7%

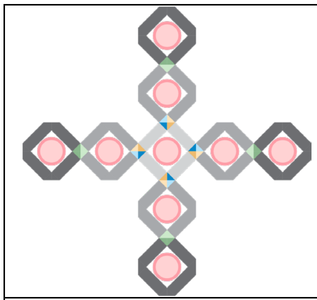	2a (1:4:4)	10%	9%
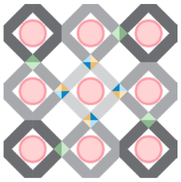	2k (1:4:4)	16%	10%
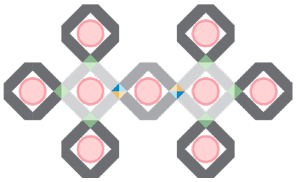	2v (1:2:6)	N/A	5%
Hetero-polymer rings			
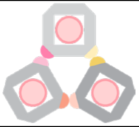	2x (1:1:1)	43%	53%
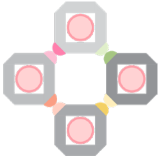	2x (1:1:1:1)	65%	16%
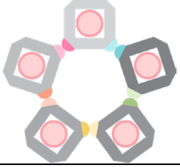	2x (1:1:1:1:1)	46%	13%
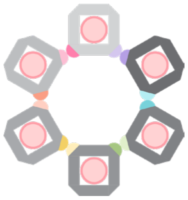	2x (1:1:1:1:1:1)	29%	5%

SDO lattices yields (column 3) were quantified using agarose gel electrophoresis and analyzed with ImageJ. In some lanes, DNA samples were retained in the wells, likely due to the formation of larger structures or aggregation. Yield analysis for the corresponding structures might be confounded by these retained samples, so we marked the values in red.

STL lattices yields (column 4) were quantified from wide-field TEM images. Yield was calculated as the number of monomers within correctly formed polymers divided by the total number of monomers observed in 5-7 images. Yields varied widely across constructs, ranging

from over 50% for the hetero-trimer to as low as 4% for some 5-mer designs. In general, smaller polymers exhibited higher yields than bigger ones. Moreover, constructs composed of equimolar monomer units (e.g., 8-mer v1) typically outperformed those with unequal stoichiometries (e.g., 8-mer v2 and v3). This is partly due to the use of excess peripheral STL monomers to saturate binding sites on core units, leaving surplus monomers unincorporated in solution.

Overall, STL assemblies showed lower yields than SDO lattices. One contributing factor is the greater difficulty in precisely controlling the stoichiometry of STL variants. In our experiments, we assumed equal formation efficiency and recovery yield for all STL variants and did not fine-tune their concentrations before mixing. As a result, the actual stoichiometry in each polymerization reaction may have deviated from the intended values listed in column 2 of the table.

Potential Applications of STL Arrays

In this work, we developed a liposome patterning platform using DNA-templated liposome formation combined with square-shaped DNA origami polymerization. Our method provides the first solution for precisely controlling both the copy number and spatial arrangement of multiple small unilamellar vesicles (SUVs). Organizing liposomes into customizable arrays extends the capabilities of traditional systems based on individual liposomes[1]. Here we highlight several niche applications that exploit the programmability and addressability of such liposome lattices.

1. Addressable (proteo)liposome arrays for electron microscopy (EM) visualization of protein-protein or protein-membrane interactions.

Protein microarrays are powerful tools for studying and detecting proteins, as well as protein-protein interactions[2, 3]. Liposome arrays serve as supportive platforms for membrane proteins[4], which constitute more than 60% of current drug targets. Most existing techniques for patterning lipid bilayers rely on top-down lithographic methods[4]. In contrast, our approach offers a bottom-up alternative with several key advantages: (1) it allows direct structural characterization of protein-protein or protein-lipid interaction using EM; (2) it requires smaller sample quantities and potentially no modification of the target protein, unless the proteins are too small to observe; (3) binding reactions occur in solution rather than on a surface, minimizing contamination and artifacts; (4) it has the potential to integrate with existing lithography methods[5], to enhance information density and sensitivity.

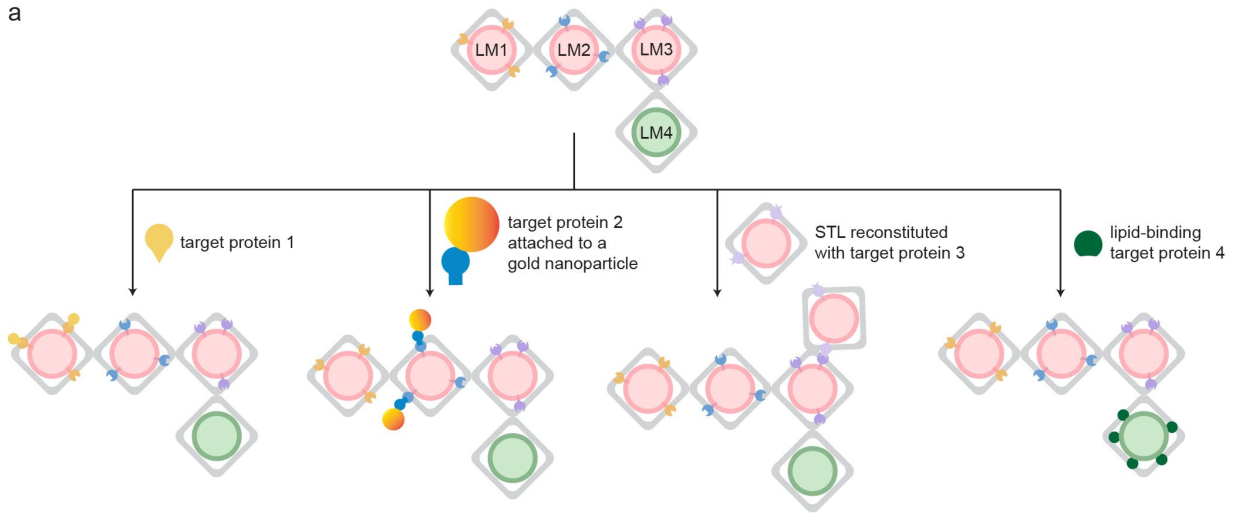

The figure above illustrates how such a 'mini-biochip' can be used for the detection and characterization of protein binding. In this example, an L-shaped hetero-tetramer composed of four liposome monomers (LM1-LM4) is preassembled and functions as an addressable chip. Upon mixing with target proteins, target 1 and target 2 specifically bind to the liposome reconstituted with their corresponding binding partners, which can then be visualized by EM. To aid imaging, tags or nanoparticles may be attached to the target proteins[6]. For interactions

between membrane proteins, target 3 can be reconstituted into a separate STL, whose binding to the array can be clearly identified. Finally, the binding of target 4 to specific lipids on LM4 demonstrates the utility of STL arrays as a versatile platform for investigating protein-lipid interactions[7].

2. Nanoreactor networks for chemical and biochemical synthesis.

The use of liposomes as chemical reaction chambers (or reactors) is appealing for several reasons[8]: (1) their internal environment can be engineered to differ from the external medium, enabling the coupling of enzymatic reactions that would otherwise be incompatible; (2) they operate at extremely low reaction volumes, on the zeptoliter to femtoliter scale, making them ideal for working with low-abundance reagents; (3) they are self-assembled structures, eliminating the need for labor-intensive nanofabrication; (4) their lipid bilayer membranes closely resemble those of biological cells, allowing for the incorporation of functional biomolecular machinery.

Current liposomal reactors are largely limited to giant unilamellar vesicles (GUVs)[9]. Although simple GUV networks or arrays have been demonstrated[10, 11], there is a growing need for high-throughput production, reduced compartment size, and more complex patterning[8]. DNA-origami-templated SUVs offer a compelling alternative: they are self-assembled, size-controllable liposomes with sub-zeptoliter volumes, making them well-suited as nanoreactors. STL lattices introduces both spatial and dynamic control to this system, which is essential for orchestrating multistep cascade reactions[12]. Within such assemblies, the close proximity of substrates and enzymes promotes rapid conversion to products, which in turn serve as substrates for downstream enzymes, reducing the buildup of reactive or toxic intermediates[13, 14].

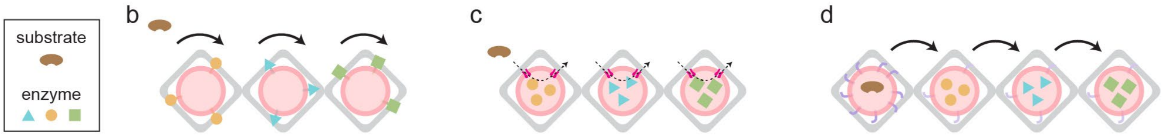

The figure above illustrates three schemes for implementing enzymatic cascade reactions using an STL array. In panel b, three different enzymes are anchored to separate membrane surfaces via transmembrane domains, enabling sequential substrate conversion at the membrane interface[15]. In panel c, the enzymes are encapsulated within individual vesicles, and the substrate enters each liposome through a membrane channel, undergoes a reaction, and exits. These channels are designed to permit substrate passage while preventing enzyme exchange. In panel d, both the substrate and the enzymes are separately enclosed in STL monomers. The substrate-loaded liposome sequentially fuses with enzyme-loaded liposomes through fusogenic mechanisms such as DNA hybridization or SNARE-mediated membrane fusion, allowing the cascade to proceed step-by-step[16]. In all three designs, the spatial organization provided by the STL array governs the order and efficiency of the cascade reaction. Notably, compared to platforms where enzymes or substrates are directly tethered to DNA or

protein nanostructures[17, 18], our method eliminates the need for DNA-protein conjugation and is inherently more compatible with membrane protein integration.

3. In vitro assays to study SNARE-mediated membrane fusion with defined stoichiometry.

The interaction between vesicular (v-) and target membrane (t-) SNARE proteins is a fundamental mechanism driving membrane fusion in eukaryotic cells[19]. This process is commonly studied using in vitro lipid and content mixing assays, where v-SNAREs and t-SNAREs are reconstituted into separate liposomes and mixed to allow fusion in solution[20]. These assays offer precise control over variables such as temperature, buffer conditions, lipid composition, and average protein copy number per vesicle, enabling the investigation of key questions that are difficult to address in the complex cellular environment. However, fusion in vitro typically occurs over several minutes, whereas in vivo, fusion can happen within seconds or even milliseconds. This discrepancy arises because, in vivo (e.g., at the neuronal terminal), (1) vesicles are captured and positioned to the active zone by chaperone proteins like Munc13, RIM, and Rab, (2) t-SNAREs form local clusters that match the v-SNARE copy number[21]. Therefore, precise spatial organization and stoichiometric balance between v- and t-vesicles are key factors for achieving faster fusion rates. STL arrays provide a platform that enables such control.

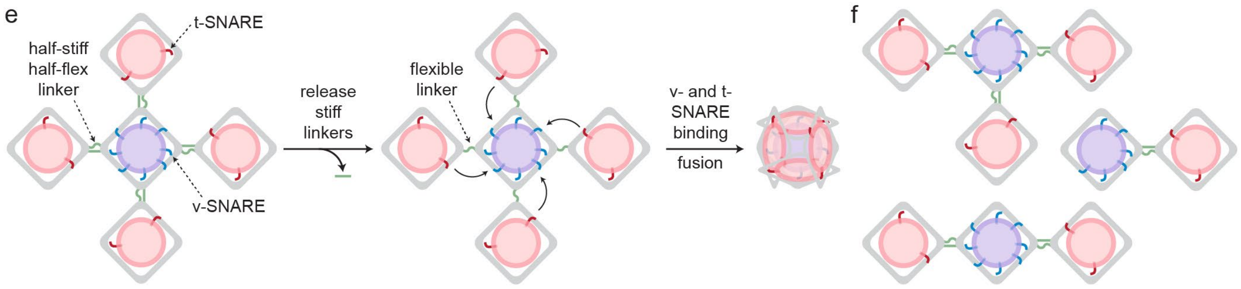

The figure above illustrates an example of an STL assay designed to study SNARE-mediated fusion. In this setup (panel e), an STL hetero-pentamer is assembled by tethering one v-liposome to four t-liposomes. Each linker consists of two sticky ends: one with a T5 spacer and the other with a T20 spacer (see Figure 2x in the main text). The cross-shaped structure of the pentamer is initially stabilized by the stiff T5 sticky ends, which are later released through TMSD. After T5 removal, the longer T20 sticky ends remain, keeping the structure tethered while allowing sufficient flexibility for v- and t-liposomes to interact and fuse. A major advantage of this platform is its ability to precisely control the stoichiometry of interacting vesicles (for instance, v-to-t liposome ratios of 1:3, 1:2, or 1:1 shown in panel f), a level of control not previously achieved. This is particularly valuable for analyzing rounds of fusion and visualizing fusion intermediates using EM. Furthermore, our STL system incorporates three built-in features beneficial for this application: (1) uniform liposome size enforced by the DNA origami scaffold, (2) customizable SNARE copy number on each vesicle via origami-templated assembly, (3) efficient STL array formation at room temperature or 4 °C (see Figure S12). These advantages, combined with stoichiometric control, also make the platform well-suited for investigating lipid transfer proteins[22], where lipid transport from a single donor liposome to varying numbers of acceptor liposomes can

be quantitatively and unambiguously characterized.

4. Bottom-up construction of synapse-like connections and organelle contacts.

Compartmentalization is a defining feature in biology as well as a central design strategy in construction of artificial cell-like systems[23, 24]. Liposome-based systems are particularly attractive due to their biomimetic properties; however, their utility is often limited by a lack of higher-order spatial and temporal control[25, 26], especially when compared to the tightly orchestrated dynamics of living cells[27, 28]. Here, we demonstrate how our STL patterning system can advance the field of bottom-up synthetic biology in two synapse-related directions.

(1) Synapse patterning. Neurons communicate with each other through specialized junctions called synapses. Mapping and manipulating synaptic connectivity are essential for understanding brain function, however, the immense complexity of neural networks presents a significant challenge. In vitro models attempt to replicate key features of in vivo network formation, with microfluidics and synaptic engineering emerging as prominent approaches[29, 30]. STL arrays provide a modular, bottom-up platform that organizes vesicles (as neuron mimics) into defined patterns, thereby prescribing the final connection topology. The figure below (panel g) illustrates examples of distinct synapse-like networks derived from three different STL tetramers. Axon-mimicking structures can be generated by membrane-remodeling proteins[31] , with their interactions mediated by specific binding moieties such as complementary DNA strands[32]. When the resulting membrane tubules also possess fusogenic properties, they can merge to create interconnected membrane networks (panel h), providing a valuable tool for investigating membrane curvature and directed molecular transport[33].

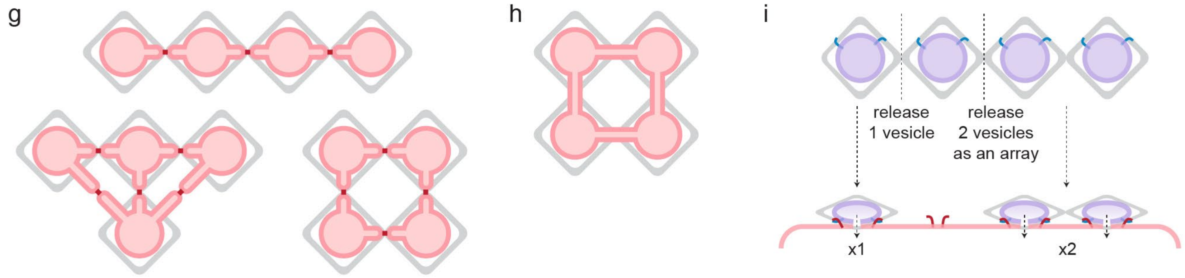

(2) Controlled quantal release. Neurotransmitters are released in the synapse in discrete units called quanta[34]. Each quantum corresponds to the contents of a single synaptic vesicle (SV), which fuses with the presynaptic membrane to release its cargo into the synaptic cleft. This quantization ensures reliable communication across synapses and allows the nervous system to modulate signaling strength by varying the number of vesicles released. Our STL arrays closely emulate the clustered organization of SVs near the active zone[35]. Importantly, we demonstrated that a specific subpopulation of STL can be selectively disassembled from the larger ensemble (see Figure 3b in the main text), effectively mimicking SV release. Moreover, multiple liposomes can be discharged as an array, with the potential to fuse with a target membrane in a concerted manner (panel i in the figure above). This adds a layer of control over “neurotransmission” and may enable mimicry of synaptic plasticity[36], underscoring the versatility of bottom-up synthetic

biology approaches[37, 38].

5. Miscellaneous applications.

Lipid vesicles have been extensively used as model membrane systems, drug delivery vehicles, micro/nano-reactors, and templates for material synthesis[39]. Our STL arrays have the potential to enhance many of the applications by providing a new dimension of control over vesicle copy number and spatial arrangement. Below, we outline three additional promising directions:

(1) Synthesis of inorganic materials. Liposomes offer unique advantages for the synthesis of hard materials, particularly in controlling crystallization dynamics and accessing metastable mineral phases. These advantages stem from their ability to exclude heterogeneous nucleators, confine reaction volumes, and replicate biomimetic environments[40]. STL lattices offer an opportunity to organize liposomes into higher-order structures, enabling spatially controlled and programmable synthesis of complex inorganic nanomaterials.

(2) Advanced drug delivery systems[41]. STL arrays functionalized with different ligands or antibodies, either on the liposome surface or the origami scaffold, enable precise control over ligand density and spatial presentation, potentially improving cell-specific uptake[42]. Moreover, STL array comprising liposomes loaded with distinct therapeutic cargos allow for combinatorial or sequential drug delivery, useful in treating complex diseases like cancer or infections where multiple pathways need to be targeted. On-demand, responsive disassembly of the array can be triggered to release liposomes as needed.

(3) Structural support for Cryo-EM studies. DNA origami superlattices have previously been used to enhance vesicle density and protect delicate samples from the air-water interface during cryo-EM sample preparation[43]. Similarly, STL arrays can provide physical shielding, act as fiducial markers for image alignment, and enhance sample homogeneity[44]. These features are especially valuable for high-resolution cryo-EM studies of membrane proteins reconstituted into liposomes.

Limitations and Future Improvements of STL arrays

In this work, we developed a liposome patterning platform based on DNA-templated liposome formation combined with the polymerization of square-shaped DNA origami. While the method demonstrates versatility and robustness, the inclusion of two additional steps, i.e., DNA origami scaffolding and higher-order assembly, introduced challenges in sample preparation and potential real-world applications. In this section, we discuss the key limitations of the current approach and propose strategies for improvement.

1. Material cost.

Our method organizes vesicles through DNA origami frames, meaning the overall quantity of STL arrays is constrained by the scale of origami production. In a typical reaction, we begin with 1.2 pmol of DNA origami, which yields over 100 µl of ~5 nM STLs (~0.5 pmol). This scale is comparable to what has been effectively used in both bulk and single-molecule assays for in vitro mechanistic studies[45, 46], supporting the feasibility of many of the proposed applications.

However, liposomes produced at the picomole scale may be insufficient for applications requiring larger quantities, such as drug delivery. A major barrier to the industrial adoption of DNA origami is its high cost, primarily due to the need for synthesizing hundreds of short oligonucleotides (i.e., staples) via the phosphoramidite method. For instance, based on current pricing from IDT ($0.84 per nucleotide for 100 nmol oligos), the estimated cost of producing 1 nmol of square-shaped DNA origami is approximately $1,000. This calculation assumes a 6:1 staple-to-scaffold ratio during assembly and a 40% recovery yield after rate-zonal purification. Encouragingly, Dietz et al. have developed a bacteriophage-based approach for large-scale staple production, which may reduce costs by up to three orders of magnitude[47]. With continued innovation in DNA origami manufacturing, we anticipate that STL array production will become increasingly affordable and scalable.

2. Yield of STL arrays.

As summarized in Table S2, the yield of STL polymerization varied significantly across different constructs. In certain cases, the correctly assembled lattices constituted only a small fraction of the total products. While unwanted byproducts can be readily identified and excluded in EM-based applications (e.g., Application 1), their presence may complicate the interpretation of solution-based assays (e.g., Applications 2 and 3). Below, we present three practical optimization strategies that could enhance the yield or purity of STL polymerization.

(1) Sticky ends optimization. Sticky end (SE) hybridization serves as the foundation for origami polymerization in our system. While we have extensively leveraged its programmability, there remains room to further improve higher-order assembly yields for specific configurations. For example, a symmetric 12-nt SE (se3) demonstrated higher efficiency in attaching terminal units with a single connection compared to the standard 8-nt SEs (e.g., se1 and se2) used elsewhere. To maximize the yield of targeted oligomers, SE designs can be customized by

carefully considering factors such as monomer connectivity, melting temperature, and sequence specificity.

(2) STL oligomer separation. Rate-zonal (rz) centrifugation has been previously used to separate DNA-coated liposomes from origami-liposome complexes (Supplementary Figure 16 in ref[48]). This technique separates particles based on their size and shape via a density gradient[49], enabling STL arrays with different unit counts and configurations to sediment at distinct rates. To determine the composition of each fraction, samples can be analyzed by agarose gel electrophoresis containing SDS and 10 mM $MgCl_2$, followed by confirmation through EM. Achieving complete separation may require optimization of the density gradient, extended centrifugation times, and more precise fractionation. Additionally, increasing the initial sample volume is recommended, as rz-centrifugation leads to dilution of the STL arrays.

(3) Stepwise assembly and purification. In our study, we demonstrated that STL oligomers can be assembled stepwise (Figures 3a and S44), providing a strategy to obtain pure complex constructs through multiple rounds of assembly and purification. For instance, a T-shaped 7-mer with only a 6% yield in a one-pot reaction can be enriched by first assembling and rz-purifying a T-shaped tetramer from monomers 1 and 2, which has a 26% yield, then adding the third monomer, potentially in several-fold excess, followed by another purification step. By focusing on a narrow size range at each stage, this method facilitates more efficient separation and simplifies optimization of rz-centrifugation parameters.

3. New designs.

The square-shaped DNA origami (SDO) has proven effective in scaffolding and organizing liposomes. By programming the SEs on each SDO, both open-ended and finite 2D lattices were successfully assembled, yielding STL arrays with unique potentials. While the general method established in this study is suitable for most of the proposed applications, further adaptation or redesign may be required to fully exploit the system's capabilities.

For example, in the context of SNARE-mediated fusion (Application 3), we introduced a new strategy that incorporates both a stiff and a flexible SE at the same vertex, enabling originally fixed connections to become pliable upon triggering. In Application 4, we propose using preassembled liposome arrays as structural platform to build synapse-like networks. Although membrane surfaces remain largely accessible within the SDO belt, a more optimal design might replace SDO with a wireframe cube origami polymerized via face-to-face docking[50], allowing unobstructed space between adjacent vesicles. A further advantage of the cube design lies in its potential to support 3D assembly[51-53], an extension beyond the 2D liposome arrays demonstrated in this work.

References

(1) Bolognesi, G.; Friddin, M. S.; Salehi-Reyhani, A.; Barlow, N. E.; Brooks, N. J.; Ces, O.; Elani, Y. Sculpting and fusing biomimetic vesicle networks using optical tweezers. *Nat. Commun.* **2018**, *9* (1), 1882.

(2) Krizkova, S.; Heger, Z.; Zalewska, M.; Moulick, A.; Adam, V.; Kizek, R. Nanotechnologies in protein microarrays. *Nanomedicine (Lond)* **2015**, *10* (17), 2743-2755.

(3) Syu, G. D.; Dunn, J.; Zhu, H. Developments and Applications of Functional Protein Microarrays. *Mol. Cell. Proteom.* **2020**, *19* (6), 916-927.

(4) Bally, M.; Bailey, K.; Sugihara, K.; Grieshaber, D.; Voros, J.; Stadler, B. Liposome and lipid bilayer arrays towards biosensing applications. *Small* **2010**, *6* (22), 2481-2497.

(5) Martynenko, I. V.; Ruider, V.; Dass, M.; Liedl, T.; Nickels, P. C. DNA Origami Meets Bottom-Up Nanopatterning. *ACS Nano* **2021**, *15* (7), 10769-10774.

(6) Chen, Y.; Huang, Y.; Yang, Y. R. DNA Nanotags for Multiplexed Single-Particle Electron Microscopy and In Situ Electron Cryotomography. *JACS Au* **2025**, *5* (1), 17-27.

(7) Sych, T.; Levental, K. R.; Sezgin, E. Lipid-Protein Interactions in Plasma Membrane Organization and Function. *Annu. Rev. Biophys.* **2022**, *51*, 135-156.

(8) Trantidou, T.; Friddin, M.; Elani, Y.; Brooks, N. J.; Law, R. V.; Seddon, J. M.; Ces, O. Engineering Compartmentalized Biomimetic Micro- and Nanocontainers. *ACS Nano* **2017**, *11* (7), 6549-6565.

(9) Elani, Y.; Law, R. V.; Ces, O. Vesicle-based artificial cells as chemical microreactors with spatially segregated reaction pathways. *Nat. Commun.* **2014**, *5*, 5305.

(10) Karlsson, A.; Karlsson, R.; Karlsson, M.; Cans, A. S.; Stromberg, A.; Ryttsen, F.; Orwar, O. Networks of nanotubes and containers. *Nature* **2001**, *409* (6817), 150-152.

(11) Liu, Z.; Liu, L.; Huang, R.; Xu, F. J.; Zhao, N.; Lin, Y. Engineering Protocell Networks for Prototissue Development. *Adv. Healthc. Mater.* **2025**, e2500376.

(12) Schmidt-Dannert, C.; Lopez-Gallego, F. A roadmap for biocatalysis - functional and spatial orchestration of enzyme cascades. *Microb. Biotechnol.* **2016**, *9* (5), 601-609.

(13) Zecchin, A.; Stapor, P. C.; Goveia, J.; Carmeliet, P. Metabolic pathway compartmentalization: an underappreciated opportunity? *Curr. Opin. Biotechnol.* **2015**, *34*, 73-81.

(14) Conrado, R. J.; Varner, J. D.; DeLisa, M. P. Engineering the spatial organization of metabolic enzymes: mimicking nature's synergy. *Curr. Opin. Biotechnol.* **2008**, *19* (5), 492-499.

(15) Tseng, Y. C.; Song, J.; Zhang, J.; Shandilya, E.; Sen, A. Chemomechanical Communication between Liposomes Based on Enzyme Cascades. *J. Am. Chem. Soc.* **2024**, *146* (23), 16097-16104.

(16) Loffler, P. M. G.; Ries, O.; Rabe, A.; Okholm, A. H.; Thomsen, R. P.; Kjems, J.; Vogel, S. A DNA-Programmed Liposome Fusion Cascade. *Angew. Chem. Int. Ed. Engl.* **2017**, *56* (43), 13228-13231.

(17) Kroll, S.; Niemeyer, C. M. Nucleic Acid-based Enzyme Cascades-Current Trends and Future Perspectives. *Angew. Chem. Int. Ed. Engl.* **2024**, *63* (5), e202314452.

(18) Seo, M.-J.; Schmidt-Dannert, C. Organizing Multi-Enzyme Systems into Programmable Materials for Biocatalysis. *Catalysts* **2021**, *11* (4), 409.

(19) Sutton, R. B.; Fasshauer, D.; Jahn, R.; Brunger, A. T. Crystal structure of a SNARE complex involved in synaptic exocytosis at 2.4 A resolution. *Nature* **1998**, *395* (6700), 347-353.

(20) Weber, T.; Zemelman, B. V.; McNew, J. A.; Westermann, B.; Gmachl, M.; Parlati, F.; Sollner, T. H.; Rothman, J. E. SNAREpins: minimal machinery for membrane fusion. *Cell* **1998**, *92* (6), 759-772.
(21) Mion, D.; Bunel, L.; Heo, P.; Pincet, F. The beginning and the end of SNARE-induced membrane fusion. *FEBS Open Bio* **2022**, *12* (11), 1958-1979.
(22) Bian, X.; Zhang, Z.; Xiong, Q.; De Camilli, P.; Lin, C. A programmable DNA-origami platform for studying lipid transfer between bilayers. *Nat. Chem. Biol.* **2019**, *15* (8), 830-837.
(23) Schmitt, C.; Lippert, A. H.; Bonakdar, N.; Sandoghdar, V.; Voll, L. M. Compartmentalization and Transport in Synthetic Vesicles. *Front. Bioeng. Biotechnol.* **2016**, *4*, 19.
(24) Wang, X.; Qiao, X.; Chen, H.; Wang, L.; Liu, X.; Huang, X. Synthetic-Cell-Based Multi-Compartmentalized Hierarchical Systems. *Small Methods* **2023**, *7* (12), e2201712.
(25) Ivanov, I.; Castellanos, S. L.; Balasbas, S., 3rd; Otrin, L.; Marusic, N.; Vidakovic-Koch, T.; Sundmacher, K. Bottom-Up Synthesis of Artificial Cells: Recent Highlights and Future Challenges. *Annu. Rev. Chem. Biomol. Eng.* **2021**, *12*, 287-308.
(26) Karoui, H.; Patwal, P. S.; Pavan Kumar, B.; Martin, N. Chemical Communication in Artificial Cells: Basic Concepts, Design and Challenges. *Front. Mol. Biosci.* **2022**, *9*, 880525.
(27) Kholodenko, B. N.; Hancock, J. F.; Kolch, W. Signalling ballet in space and time. *Nat. Rev. Mol. Cell Biol.* **2010**, *11* (6), 414-426.
(28) de Brito, O. M.; Scorrano, L. An intimate liaison: spatial organization of the endoplasmic reticulum-mitochondria relationship. *EMBO J.* **2010**, *29* (16), 2715-2723.
(29) Habibey, R.; Rojo Arias, J. E.; Striebel, J.; Busskamp, V. Microfluidics for Neuronal Cell and Circuit Engineering. *Chem. Rev.* **2022**, *122* (18), 14842-14880.
(30) Rabinowitch, I.; Colon-Ramos, D. A.; Krieg, M. Understanding neural circuit function through synaptic engineering. *Nat. Rev. Neurosci.* **2024**, *25* (2), 131-139.
(31) Beales, P. A.; Ciani, B.; Cleasby, A. J. Nature's lessons in design: nanomachines to scaffold, remodel and shape membrane compartments. *Phys. Chem. Chem. Phys.* **2015**, *17* (24), 15489-15507.
(32) Yu, H.; Liang, Z.; Zhu, L.; Zhao, C.; He, X.; Chen, K.; Xu, W. Nucleic Acid-Modified Liposome: Construction Methods and Biological Applications (Adv. Mater. Interfaces 3/2022). *Adv. Mater. Interfaces* **2022**, *9* (3), 2270017.
(33) Wegrzyn, I.; Zhang, H.; Orwar, O.; Jesorka, A. Nanotube-interconnected liposome networks. *Nano Commun. Netw.* **2011**, *2* (1), 4-15.
(34) Edwards, R. H. The neurotransmitter cycle and quantal size. *Neuron* **2007**, *55* (6), 835-858.
(35) Milovanovic, D.; De Camilli, P. Synaptic Vesicle Clusters at Synapses: A Distinct Liquid Phase? *Neuron* **2017**, *93* (5), 995-1002.
(36) Glasgow, S. D.; McPhedrain, R.; Madranges, J. F.; Kennedy, T. E.; Ruthazer, E. S. Approaches and Limitations in the Investigation of Synaptic Transmission and Plasticity. *Front. Synaptic Neurosci.* **2019**, *11*, 20.
(37) Jia, H.; Schwille, P. Bottom-up synthetic biology: reconstitution in space and time. *Curr. Opin. Biotechnol.* **2019**, *60*, 179-187.
(38) Gonzales, D. T.; Zechner, C.; Tang, T. Y. D. Building synthetic multicellular systems using bottom–up approaches. *Curr. Opin. Syst. Biol.* **2020**, *24*, 56-63.
(39) Jesorka, A.; Orwar, O. Liposomes: technologies and analytical applications. *Annu. Rev. Anal. Chem.*

2008, *1*, 801-832.
(40) Dong, R.; Liu, W.; Hao, J. Soft vesicles in the synthesis of hard materials. *Acc. Chem. Res.* **2012**, *45* (4), 504-513.
(41) Hu, Q.; Li, H.; Wang, L.; Gu, H.; Fan, C. DNA Nanotechnology-Enabled Drug Delivery Systems. *Chem. Rev.* **2019**, *119* (10), 6459-6506.
(42) Wang, Z.; Sun, P.; Su, J.; Zhang, N.; Gu, H.; Zhao, Y. DNA nanotechnology-facilitated ligand manipulation for targeted therapeutics and diagnostics. *J. Control. Release* **2021**, *340*, 292-307.
(43) Aissaoui, N.; Mills, A.; Lai-Kee-Him, J.; Triomphe, N.; Cece, Q.; Doucet, C.; Bonhoure, A.; Vidal, M.; Ke, Y.; Bellot, G. Free-Standing DNA Origami Superlattice to Facilitate Cryo-EM Visualization of Membrane Vesicles. *J. Am. Chem. Soc.* **2024**, *146* (19), 12925-12932.
(44) Yang, Y.; Wang, J.; Shigematsu, H.; Xu, W.; Shih, W. M.; Rothman, J. E.; Lin, C. Self-assembly of size-controlled liposomes on DNA nanotemplates. *Nat. Chem.* **2016**, *8* (5), 476-483.
(45) Zimmermann, J. A.; Lucht, K.; Stecher, M.; Badhan, C.; Glaser, K. M.; Epple, M. W.; Koch, L. R.; Deboutte, W.; Manke, T.; Ebnet, K.; et al. Functional multi-organelle units control inflammatory lipid metabolism of macrophages. *Nat. Cell Biol.* **2024**, *26* (8), 1261-1273.
(46) Xu, W.; Nathwani, B.; Lin, C.; Wang, J.; Karatekin, E.; Pincet, F.; Shih, W.; Rothman, J. E. A Programmable DNA Origami Platform to Organize SNAREs for Membrane Fusion. *J. Am. Chem. Soc.* **2016**, *138* (13), 4439-4447.
(47) Praetorius, F.; Kick, B.; Behler, K. L.; Honemann, M. N.; Weuster-Botz, D.; Dietz, H. Biotechnological mass production of DNA origami. *Nature* **2017**, *552* (7683), 84-87.
(48) Zhang, Z.; Feng, Z.; Zhao, X.; Jean, D.; Yu, Z.; Chapman, E. R. Functionalization and higher-order organization of liposomes with DNA nanostructures. *Nat. Commun.* **2023**, *14* (1), 5256.
(49) Lin, C.; Perrault, S. D.; Kwak, M.; Graf, F.; Shih, W. M. Purification of DNA-origami nanostructures by rate-zonal centrifugation. *Nucleic Acids Res.* **2013**, *41* (2), e40.
(50) Bohlin, J.; Turberfield, A. J.; Louis, A. A.; Sulc, P. Designing the Self-Assembly of Arbitrary Shapes Using Minimal Complexity Building Blocks. *ACS Nano* **2023**, *17* (6), 5387-5398.
(51) Zhou, Y.; Dong, J.; Zhou, C.; Wang, Q. Finite Assembly of Three-Dimensional DNA Hierarchical Nanoarchitectures through Orthogonal and Directional Bonding. *Angew. Chem. Int. Ed. Engl.* **2022**, *61* (13), e202116416.
(52) Weck, J. M.; Heuer-Jungemann, A. Fully addressable designer superstructures assembled from one single modular DNA origami. *Nat. Commun.* **2025**, *16* (1), 1556.
(53) Li, X.; Wang, J.; Baptist, A.; Wu, W.; Heuer-Jungemann, A.; Zhang, T. Crystalline Assemblies of DNA Nanostructures and Their Functional Properties. *Angew. Chem. Int. Ed. Engl.* **2025**, *64* (3), e202416948.
